# End-of-Life Decision-Making in Canada: The Report by the Royal Society of Canada Expert Panel on End-of-Life Decision-Making

**DOI:** 10.1111/j.1467-8519.2011.01939.x

**Published:** 2011-11

**Authors:** Udo Schüklenk, Johannes J M Van Delden, Jocelyn Downie, Sheila A M Mclean, Ross Upshur, Daniel Weinstock

**Affiliations:** Department of Philosophy, Queen's UniversityCanada; UMC Utrecht, Julius CenterThe Netherlands; Faculties of Law and Medicine, Dalhousie UniversityCanada; University of GlasgowUK; Department of Family and Community Medicine University of TorontoCanada; Department of Philosophy, Université de MontréalCanada

**Keywords:** Canada, Royal Society report, end-of-life, quality of life, assisted dying, palliative care, death

## Abstract

This report on end-of-life decision-making in Canada was produced by an international expert panel and commissioned by the Royal Society of Canada. It consists of five chapters.

Chapter 1 reviews what is known about end-of-life care and opinions about assisted dying in Canada.

Chapter 2 reviews the legal status quo in Canada with regard to various forms of assisted death.

Chapter 3 reviews ethical issues pertaining to assisted death. The analysis is grounded in core values central to Canada's constitutional order.

Chapter 4 reviews the experiences had in a number of jurisdictions that have decriminalized or recently reviewed assisted dying in some shape or form.

Chapter 5 provides recommendations with regard to the provision of palliative care in Canada, as well as recommendations for reform with respect to the various forms of assisted death covered in this document.

## TABLE OF CONTENTS

### INTRODUCTION

1. Introductory Remarks and Objectives
2. Terminology
3. Outline

### CHAPTER ONE: END-OF-LIFE CARE IN CANADA

1. Introduction
2. Canadian Experience at the End of Life
  - Mortality and Life Expectancy Trends in Canada
  - Location of Death
  - Quality of and Access to Palliative Care
3. Expanding the Range of Palliative Care
  - Dementia
  - Chronic Kidney Disease
  - Congestive Heart Failure
  - Chronic Obstructive Pulmonary Disease
  - Disability
4. Demographic Transition in Canada
  - Aging
  - Diversity
  - First Nations
5. Advance Directives and Substitute Decision-Making
6. Sedation Practices
7. Paediatric End of Life Care
8. Attitudes of Canadians Toward Voluntary Euthanasia and Assisted Suicide
  - General Public
  - Health Care Professionals
  - Patients
9. International Comparisons
10. Conclusions

### CHAPTER TWO: THE LEGAL LANDSCAPE

1. Introduction
2. Withholding and Withdrawal of Potentially Life-sustaining Treatment
  - Relatively Clear and Uncontroversial
  - Less Clear and More Controversial
  - Very Unclear and Very Controversial
3. Potentially Life-shortening Symptom Relief
  - Somewhat Clear and Relatively Uncontroversial
4. Terminal Sedation
  - Very Unclear and Potentially Very Controversial
5. Assisted Suicide
  - Very Clear and Very Controversial
6. Voluntary Euthanasia
  - Very Clear and Very Controversial
7. Conclusions

### CHAPTER THREE: THE ETHICS OF END-OF-LIFE CARE

1. Introduction
2. Core Values
3. Autonomy
4. Moral and Legal Rights
5. Autonomy and Assisted Death
6. Limits to the Right to Medically Assisted Death
  - No Inference from the Right to Refuse Treatment to the Right to Assisted Death
  - A Priori Arguments: Suicide is not Choice-worthy
  - A Priori Arguments: Suicide Offends against Human Dignity
7. Arguments against the *Legal* Right to Assisted Death
  - Medical Professionals
  - Slippery Slopes and the Protection of the Vulnerable
8. Conclusions

### CHAPTER FOUR: INTERNATIONAL EXPERIENCE WITH LAWS ON ASSISTED DYING

1. Introduction
2. Mechanisms for Change to Law and/or Practice
  - Judicial Decisions (Netherlands, Montana)
  - Prosecutorial Charging Guidelines (Netherlands, United Kingdom)
  - New or Revised Laws
    - The Netherlands
    - Belgium
    - Luxemburg
    - Oregon
    - Washington State
  - Evolution of Practice Without Legal Change (Switzerland)
3. Elements of Regulated Permissive Regimes
4. Practical Experience
  - Data on Voluntary Euthanasia and Assisted Suicide
    - Netherlands
    - Belgium
    - Switzerland
    - Oregon
    - Washington State
  - Slippery Slopes?
5. Conclusions

### CHAPTER FIVE: PROPOSALS FOR REFORM

1. Introduction
2. Withholding and Withdrawal of Potentially Life-sustaining Treatment
  - Valid Refusals by Competent Adults (or Legally Authorized Substitute Decision-makers)
  - Mature Minors
  - Unilateral Withholding and Withdrawal
3. Advance Directives
4. Palliative Care
5. Potentially Life-shortening Symptom Relief
6. Terminal Sedation
7. Assisted Suicide and Voluntary Euthanasia
  - Legal Mechanisms
    - Criminal Code Reform
    - Prosecutorial Charging Guidelines
    - Diversion Programs
  - Core Elements
    - Assisted Suicide and/or Voluntary Euthanasia
    - Features of the Person
    - Features of the Decision
    - Features of the Person's Condition
    - Features of the Request for Assistance
    - Features of the Provider
    - Oversight and Control

