# Supplementary material for: End-of-Life Decision-Making in Canada: The Report by the Royal Society of Canada Expert Panel on End-of-Life Decision-Making
Source: Bioethics. 2011 Nov;25(Suppl 1):1–4. doi: 10.1111/j.1467-8519.2011.01939.x (PMC3265521; doi:10.1111/j.1467-8519.2011.01939.x)
Supplement: Related texts

# Sub article: INTRODUCTION

**Published:** 2011-11

## 1. Introductory Remarks and Objectives

The last comprehensive federal public policy report on assisted death in Canada was published 15 years ago.^1^ Since then much has taken place in this area. Advance directives legislation has been introduced and reformed in a number of provinces and territories.^2^ A substantial number of court cases have involved various aspects of assisted death. A number of these high-profile cases have captured public attention and been reported extensively in the national press; the names of Nancy Morrison, Samuel Golubchuk, Robert Latimer and Evelyn Martens are but a few examples known to many Canadians because of their connections to these widely reported, high-profile end-of-life cases.^3^ Beyond our borders, assisted suicide or voluntary euthanasia is now legal in at least seven jurisdictions. They are the Netherlands, Belgium, Luxemburg, Switzerland, Oregon, Washington State, and Montana.^4^ In addition, prosecution policy in England and Wales has been clarified to make it clear that not all instances of assisted suicide will result in prosecution.^5^

Despite all this activity, three very important features of the landscape have not changed. First, public support for the decriminalization of assisted suicide and voluntary euthanasia remains high (a substantial majority of Canadians support the decriminalization of assisted dying). Second, the issue of decriminalization remains very contentious and polarized. Third, regardless of this support and no doubt in part because of this contentiousness, assisted suicide and euthanasia remain prohibited activities under the *Criminal Code of Canada*.^6^

It is, therefore, an apt time to revisit the public policy questions surrounding assisted death, in the light of new evidence and arguments. In 1995, a majority of the Special Senate Committee on Euthanasia and Assisted Suicide recommended continuing to treat euthanasia as murder (albeit with a lesser penalty), and the assisted suicide provision in the *Criminal Code*.^7^ Would a careful consideration of these issues in 2010 come to the same conclusions?

In order to address this question, and to both catalyze and contribute to a process of public reflection on these critically important public policy issues, the Royal Society of Canada established the Expert Panel on End of Life Decision-Making (RSC EOL Panel) with the following objectives:

1. There is a large body of medical science evidence that, if summarized for the public, would be helpful to their consideration of the issue.
2. The public could also benefit from a presentation of evidence about actual experience from the various jurisdictions that permit physician-assisted death.
3. The public would also benefit greatly from having a careful, balanced review of various pros and cons of decriminalization of physician-assisted death from well-reasoned ethical and legal standpoints.
4. Many medical personnel would also benefit from having all the issues laid out in a comprehensive and sensitive way.
5. The Panel should consider proposing policy recommendations for public consideration that are the results of its review.

The members of the RSC EOL Panel were experts in the following areas relevant to the issues the Panel was tasked to address: bioethics, clinical medicine, health law and policy, and philosophy.

The members of the Panel met in person and conducted business via e-mail and phone. Panel members brought their own expertise and experience to the project and additional research was conducted as required. Drafts were circulated and edited through a collaborative and iterative process.

This document presents the unanimous Final Report of the RSC EOL Panel. The Panel trusts that it will serve as a marker for the beginning of a new conversation about end-of-life law, policy, and practice in Canada. The Panel notes that the conversation will require mutual attention and respect and acknowledges the many important interests at stake and values in play. Passions run deep in discussions about end-of-life matters. However, even in the face of profound disagreements (about, for example, the values of autonomy and life), it is possible – and indeed necessary – for those involved in the conversation to listen carefully to all positions presented and to work together to find a policy position consistent with the core features of Canada's parliamentary democracy and our *Charter of Rights and Freedoms*.^8^ The Panel hopes that, through this conversation, all stakeholders will find common ground to respond better to the wishes and needs of Canadians at the end of their lives.

## 2. Terminology^9^

It is particularly important to define the terms employed in discussions about assisted death. Frequently people discuss these issues at cross-purposes, using the same term to describe different practices or using different terms to describe the same practice. This often leads to unnecessary and unproductive confusion and conflict. As there are, by necessity, no objectively true definitions of the terms needed to discuss assisted death, the Panel stipulates the following definitions for the purposes of this Report:

- *‘Withholding of potentially life-sustaining treatment’* is the failure to start treatment that has the potential to sustain a person's life. An example is not providing cardiopulmonary resuscitation to a person having a cardiac arrest.
- *‘Withdrawal of potentially life-sustaining treatment’* is stopping treatment that has the potential to sustain a person's life. An example is the removal of a ventilator from a patient with a devastatingly severe head injury after a motorcycle accident with no prospect of improvement.
- *‘Advance directives’* are directions given by a competent individual concerning what and/or how and/or by whom decisions should be made in the event that, at some time in the future, the individual becomes incompetent to make healthcare decisions. An example is a woman who has signed a document that states that, should she fall into a persistent vegetative state, she does not wish to receive artificial hydration or nutrition. Or, as another example, a man who has signed a document that states that, when he is incompetent, he wishes his wife to make all healthcare decisions on his behalf. There are two kinds of advance directives: *instruction directives*, which establish what and/or how healthcare decisions are to be made; and *proxy directives*, which establish who is to make healthcare decisions.
- *‘Potentially life-shortening symptom relief’* is suffering-control medication given in amounts that may – but are not certain to – shorten a person's life. An example is giving ever-increasing levels of morphine necessary to control an individual's suffering from terminal cancer when the morphine is known to potentially depress respiration even to the point of causing death (but it is not known precisely how much is too much as the levels are slowly increased).
- *‘Palliative sedation’* is an umbrella term used to explain intermittent and continuous as well as superficial and deep sedation. The most contested subtype of *palliative sedation* is known as *‘terminal sedation*’.
- *‘Terminal sedation’* is potentially life-shortening deep and continuous sedation intentionally combined with the cessation of nutrition and hydration.
- *‘Assisted suicide’* is the act of intentionally killing oneself with the assistance of another. An example is a woman with advanced ALS who gets a prescription from her physician for barbiturates and uses the drugs to kill herself.
- *‘Voluntary Euthanasia’* is an act undertaken by one person to kill another person whose life is no longer worth living to them in accordance with the wishes of that person. An example is a man bedridden with many of the consequences of a massive stroke whose physician, at his request, gives him a lethal injection of barbiturates and muscle relaxants.
- *‘Unilateral’* means without the knowledge of or – less commonly – against the wishes of the patient or patient's substitute decision-maker. An example is a physician who writes a Do Not Resuscitate order on a patient's chart without consulting the patient or the patient's substitute decision-maker.
- *‘Competent’* means capable of understanding and appreciating the relevant information and the nature and consequences of the decision to be made. It is important to note that competence is decision-, time-, and place-specific and that individuals may be competent for one decision (such as what to eat and drink) and not another (such as whether to refuse surgery) and may be competent one day and not the next.
- *‘Voluntary’* means in accordance with the wishes expressed by a competent person or through a valid advance directive.
- *‘Non-voluntary’* means without the knowledge of the wishes expressed by a competent person or through a valid advance directive.
- *‘Involuntary’* means against the wishes expressed by a competent person or through a valid advance directive.
- *‘Assisted dying’* is an umbrella term used to describe the full spectrum of conduct defined above that contributes to the death of an individual.

## 3. Outline

This Report proceeds as follows:

- First, the Panel describes what is known about social attitudes and practices with respect to the full spectrum of end-of-life care in Canada. The Panel considers: the Canadian experience at the end of life with respect to mortality and life expectancy, location of death, and quality of and access to palliative care; expanding the range of palliative care beyond cancer; demographic transition in Canada with respect to aging and cultural and ethnic diversity; sedation practices; and paediatric end-of-life care. The Panel also considers practices with respect to substitute decision-making for those who have never been or are no longer capable of making their own health care decisions. This section ends with a review of survey results of the attitudes to end-of-life issues among health professionals and the public in Canada and abroad.
- Second, the Panel presents an overview of the legal status of all of the various forms of assisted death in Canada. The law is relatively clear and uncontroversial with respect to the withholding and withdrawal of potentially life-sustaining treatment in some circumstances (for example, competent adults), but unclear and controversial in others (such as withholding or withdrawal from mature minors and unilateral withholding and withdrawal). The law is insufficiently clear, but relatively uncontroversial, with respect to potentially life-shortening symptom relief. It is unclear and controversial with respect to terminal sedation. And, finally, the law is clear and very controversial with respect to assisted suicide and euthanasia.
- Third, the Panel turns its attention to the ethics of assisted death. It grounds the subsequent analysis in core values central to Canada's constitutional order, explores ways in which legal rights can be argued for, and shows how autonomy (the principal core value) can best be protected through legal rights. Applying this to the issues of assisted suicide and euthanasia, it concludes that there is a strong argument for a moral right to choose euthanasia and assisted suicide and that the arguments others have proposed to support limiting these rights are flawed. The Panel considers arguments concerning autonomy and dignity. The Panel also engages with a number of arguments that have been particularly enduring in this area: for example, the distinction between deliberately killing and letting die; the doctrine of double-effect (also known as the intention-foresight distinction); and arguments about slippery slopes. A number of myths and logical errors commonly found in the literature and public debate on these topics are exposed in this chapter.
- Fourth, having concluded that, on ethical grounds, Canada should have a permissive yet carefully regulated and monitored system with respect to assisted death, the Panel turns its attention to the question of how to achieve such a system. To assist with answering that question, the Panel describes the regulation of assisted death in those jurisdictions in the world where assisted suicide and/or voluntary euthanasia have, to some extent and by various means, become more permissible (whether by legislative changes such as in Oregon, or changes in prosecution policy such as in England and Wales). The experiences in the Netherlands, Belgium, Luxemburg, Switzerland, Oregon, Washington State, and Montana, are reviewed with a particular focus on how permissive systems have been designed – what legal mechanisms have been used and what positions have been taken on key decision points within permissive regimes – and what has happened following the introduction of a permissive system.
- Fifth and finally, the Panel provides recommendations with regard to the provision of palliative care in Canada, as well as recommendations for reform with respect to the various forms of assisted death canvassed in the Report. These recommendations are based on the critical foundation laid in the preceding four chapters. The recommendations, of necessity, are directed at a range of agents, since the jurisdiction over these activities is dispersed among different levels of government and sectors.

A review of the national press over the past eighteen months reveals how topical the issue of assisted death is in Canadian culture. In the Spring of 2011, three cases were filed in British Columbia each, in effect albeit via different paths, challenging the *Criminal Code* prohibitions against assisted suicide and euthanasia.^10^ On June 8, 2010, a major report was issued by Senator Sharon Carstairs revealing ongoing problems with access to quality palliative care for Canadians.^11^ On April 21, 2010, the latest in a long string of private members' bills to decriminalize assisted suicide and euthanasia was defeated in the House of Commons,^12^ despite a 2010 poll indicating that a majority of Canadians support the legalization of euthanasia.^13^ In the Spring of 2010, a non-partisan Committee of the Quebec National Assembly studied the issues and launched a public consultation process.^14^ These are strong indications that Canadians are, and should be, engaged in a process of deliberation over the legal status of assisted death in Canada. The Panel offers this Report as its contribution to this important public policy debate.

# Sub article: CHAPTER ONE: END-OF-LIFE CARE IN CANADA

**Published:** 2011-11

## 1. INTRODUCTION

The spectrum of issues associated with end of life – such as advance-care planning, assisted suicide and euthanasia – are matters of great public interest and concern. Determining the relative merits of various policy and legislative options depends as much upon a detailed understanding of current social attitudes and contemporary realities of death and dying in Canada as it depends upon legal and philosophical analysis. For an informed discussion of assisted suicide and euthanasia as last-resort interventions, it is important to understand current epidemiological, clinical and policy forces that can influence the need or desire to access these modalities at the end of life.

End of life can be understood as a continuum of events starting with the diagnosis of one or more serious illnesses or injury. Each of these conditions has a trajectory, some more predictable than others. The range of illnesses relevant to end-of-life decision-making is broad; this range encompasses the leading causes of death in the population such as cardiovascular disease, pulmonary disease and cancer. There are many transitions in health status on the path from diagnosis to treatment and, finally, death. Understanding how this process works and how well it is managed in Canada will help to set the context for the consideration of the legal and ethical issues that attend decisions at the end of life. Facts inform the law and ethics, but they do not determine them.

There is good reason to be concerned with the state of end-of-life care in Canada. Canada ranked in the top 10 in a recent report from the Economist Intelligence Unit comparing the quality of death in 40 countries. Canada scored well for quality of end-of-life care and access to opiates for pain control. However, it was in the middle of the pack in terms of public awareness of end-of-life care and scored poorly in terms of costs. The report states that the ‘medicalization of death in Canada has engendered a culture where many people are afraid to raise the topic of death.’^15^

This chapter comprises several sections, including an examination of how and where Canadians are dying, as well as the changing demographic landscape (specifically an aging and increasingly diverse population and exponential growth in chronic diseases relevant to end-of-life discussions). This chapter includes a survey of research and policy literature to identify important considerations in the provision of quality end-of-life care. Several elements of quality end-of-life care are important here, including both access to palliative care services to mitigate/ manage symptoms and provide comfort to the dying, and the use of advance directives to ensure that treatment wishes at the end of life are respected when an individual is no longer competent to make decisions, or is incapable of expressing wishes. In this chapter, Canadian attitudes towards assisted suicide and euthanasia are examined and compared with other nations.

The research in this section is drawn from the academic literature and relevant non-academic sources such as government reports. Preference is given to Canadian reports, data sources and published studies. Systematic reviews of both relevant literature and recently published international studies are also included.

## 2. CANADIAN EXPERIENCE AT THE END OF LIFE

### a. Mortality and Life Expectancy Trends in Canada

The latest data for deaths in Canada is from the Statistics Canada report *Deaths 2007* released on 23 February 2010. This report highlights significant changes in life expectancy in Canada. Essentially, the most important gain in the decade from 1995–97 to 2005–07 is an increase of 2.3 years in life expectancy at birth. This gain was higher among men; male life expectancy at birth rose by 2.9 years in this time frame; for women it increased by only 1.8 years. Most significantly, life expectancy at age 65 is 19.8 years – meaning that a person at the age of 65 in 2007 has a life expectancy of about age 85. This is an indication that Canadians are living longer, as increased life expectancy of those at age 65 accounts for 70% of the total increase in life expectancy at birth.

In 2007 there were 235,217 deaths, representing a 3.1% increase from the previous year. However the age-standardized death rate of seven deaths per 1,000 members of the population remained stable. In general, the mortality rate is higher in men than women. The mortality rate per 1,000 members of the population has a J-shaped curve. The death rate per 1,000 members of the population is 5.1 per 1,000 people under one year of age. However, for those aged between one to four, and 35 to 39, the death rate per 1,000 members of the population is under one per 1,000. The death rate generally tends to increase dramatically after age 60. The mortality rates per 1,000 members of the population in 2007 were: 8.2 per 1,000 of those aged 60–64; 13.3 for those aged 65–69; 21.4 for those aged 70–74; 34.9 for those aged 75–79; 58.4 for those aged 80–84; 100.6 for those aged 85–89; and 196.5 for those 90 years and over. This indicates clearly that the vast majority of deaths occur in advanced age groups.

Among the top 10 leading causes of death in 2007, chronic diseases predominate. Cancer, cardio/cerebro-vascular diseases, and chronic lower respiratory diseases account for 62% of deaths in Canada. Alzheimer's disease and kidney disease are also important causes of death.

### b. Location of Death

Research suggests that most Canadians desire to die at home. Studies conducted on locations of death over the past decade have shown some trends in that direction; however, the vast proportion of deaths – particularly those associated with chronic diseases – occur in institutional settings. Statistics Canada data indicate that 68.6% of Canadians die in a hospital and 31.4% die elsewhere. There is, however, considerable variance across Canada. Quebec and Manitoba have the highest rates of death in hospital at 86% and British Columbia has the lowest at 49.5%.

Wilson and colleagues studied 1,806,318 deaths from 1994–2004 across Canada (excluding Quebec).^16^ A decline in hospitalized deaths was found (77.7% dropped to 60.6%). These authors noted that this decline did not vary by age, gender, marital status, or whether the deceased lived in an urban or rural locale. Heyland and colleagues note that the majority of deaths in Canada occur in hospitals with a substantial proportion occurring in special care units. They argue that this phenomenon raises questions about the appropriateness and quality of current end-of-life care practices in Canada.^17^

### c. Quality of and Access to Palliative Care

The Panel's review of the literature indicates that most Canadians die principally of old age and progressive ill health. It is estimated that 95% of deaths would benefit from palliative care, yet as many as 70% of Canadians lack access because hospice and palliative care programs are unevenly distributed across Canada. Thus, concerns are expressed about uncoordinated and poor quality end-of-life care, a point that recurs in the literature over the past decade.^18^

As Chochinov and Kristjanson note, there will be an approximate doubling in the number of seniors in the next 20 years, largely driven by the baby boom, which exacerbates the need for discussion of end of life issues.^19^ They conclude that costs at the end of life are considerable and that costs of care are highest the greater the distance from the home setting. It should be noted that family costs at the end of life are substantial and are often not factored into cost analyses. Also, some treatments given to dying patients are costly while yielding little benefit. There are few financial supports for families to take leave from work to assist in end-of-life care. Indeed the Carstairs report identified several mechanisms to reduce the financial burden of end-of-life care and facilitate family involvement in that care. Teno and colleagues, in a study published in the *Journal of the American Medicine Association* in 2004, indicate that there are considerable unmet needs for symptom amelioration, physician communication, and psychosocial support for dying individuals and their family members.^20^ Those who receive palliative care services at home are more likely to report a favourable dying experience.

In 2000, the Quality End-of-Life Care Coalition of Canada released an action blueprint to ensure access to high-quality palliative care for all Canadians. However, in a ten-year report on the progress towards reaching their goals, they note that, despite some success, most Canadians still do not have access to palliative care services. They state:

In 2007, the federal government disbanded the End-of-Life Care Secretariat and stopped work on the national palliative and end-of-life care strategy. Although palliative and end-of-life care have been included in other national strategies, such as the cancer, heart health and HIV/AIDS strategies, few specific steps have been taken to enhance services for people who are dying or to support their caregivers.^21^

## 3. EXPANDING THE RANGE OF PALLIATIVE CARE

Historically, end-of-life decision-making and considerations of palliative care centred on the management of end-stage cancer and the treatment of associated pain. An aging population and the increase of chronic diseases have expanded the need for end-of-life planning and palliative care for a broader range of conditions. Four chronic diseases pose particular challenges for end-of-life care. They are dementia, kidney disease, heart disease (particularly chronic congestive heart failure), and chronic obstructive pulmonary disease. Recent Canadian studies have noted significant unmet needs in patients with these diseases and identified opportunities for improving care, as well as noting the need for further research.

### a. Dementia

Dementia and cognitive impairment raise particular challenges for end-of-life care. Canadians are developing dementia at such a rapid rate that dealing with the problem will cost a total of more than C$870 billion ($835 billion) over the next 30 years. The Alzheimer Society of Canada states that more than 103,700 people developed dementia in 2008 in Canada, a country of around 33 million.^22^ By 2038, 257,800 new cases per year are expected.

Dementia poses unique ethical challenges at the end of life, as cognitive impairment often negates the capacity of individuals to make and express autonomous choices. Preferences expressed when competent may change as cognition declines, giving rise to complexities with regards to ascertaining what the true preference for care may be. The UK based Nuffield Council on Bioethics produced a comprehensive report on ethical issues surrounding dementia.^23^ The report, based on extensive public consultation, provides a comprehensive overview of the many ethical issues raised by the provision of dementia care including a comprehensive discussion of palliative care and end-of-life decision-making. The Council notes that patients with dementia are less likely to be offered palliative care services compared to those without dementia.

In comparison to the UK, there is a relative lack of research on the perspectives of Canadian patients with dementia toward end-of-life care. This is a concern, given the rapid rise of this disease in the aging population and the corresponding need to equip healthcare practitioners to manage these patients' unique and complex needs as the disease progresses to terminal status. A lack of adequate training and education in palliative care (in general and specifically related to patients with dementia) across various healthcare disciplines is a constant theme in the palliative care literature.^24^ Research on caregivers of dementia patients is more widely available, and focuses especially on the burdens – emotional, physical, psychological, and financial –associated with caring for these patients. In a recent survey of Canadian caregivers of patients with Alzheimer's disease and other dementia-related diseases, respondents reported negative effects on emotional health including: increased depression; stress and fatigue; stress on finances; and stress on their work situation, including being forced to retire early or reduce work hours.^25^ This survey also reports that the greatest burden is placed on live-in caregivers versus those who do not live with the patients.^26^ Canadian nurses in a long-term care setting have reported other factors in caring for dying patients with dementia that complicate care management. For example, the patient's inability to recognize his or her own terminal decline, the difficulty in predicting the disease trajectory, and dealing with the ‘responsive, self-protective behaviour’ of the patients while trying to comfort them.^27^ These findings suggest the need for interventions to help address the pressures on caregivers of patients with dementia and the challenges in managing their care.

### b. Chronic Kidney Disease

Chronic kidney disease is another key cause of mortality in Canada. Davison evaluated the preferences of patients with chronic kidney disease (CKD) related to end-of-life care.^28^ She notes that little research has been conducted to assess patients' preferences, in spite of the high mortality rate in this patient population. Her survey of 584 CKD cases shows that participants rely on the nephrology staff for extensive end-of-life care needs not currently systematically integrated into their renal care, such as pain and symptom management, advanced-care planning, and psychosocial and spiritual support. Additionally, patients report poor knowledge of their options with respect to palliative care as well as their illness trajectory. Only a small minority of patients (10%) said they had had a discussion about end-of-life care with their nephrologist during the past 12 months. Significantly, 61% of patients say they regretted their decision to start dialysis. Davison concludes that the needs of patients with CKD are not met by existing end-of-life practices.

### c. Congestive Heart Failure

A substantial number of deaths occur each year from cardiovascular disease (CVD). In their recent study, Howlett et al. note that the current model of care focuses on acute exacerbations; a comprehensive approach to managing the inevitable death that ensues from cardiovascular disease does not exist.^29^ They advocate the need for a comprehensive strategy which would incorporate end-of-life planning and care; this would foster earlier and more integrated comprehensive care. The key elements of this strategy involve the provision of advance-care planning, palliative care, hospice care and advance directives, with a focus on decision making and planning. Howlett and colleagues argue that end-of-life planning and care should be a routine part of the assessment of any patient with CVD, and should be reassessed whenever important clinical changes occur. They note the need for further research into effective end-of-life planning and care and recommend that heart failure be considered an ideal condition for implementing and testing of interventions to improve end-of-life planning and care.

Strachan and colleagues surveyed 106 patients with advanced heart failure in five Canadian tertiary care centres to elicit their perspectives about end-of-life care, including their preferences and level of satisfaction with different aspects of end-of-life care.^30^ The goal was to identify key opportunities from the patients' perspectives for improving end-of-life care for patients with advanced heart failure. A significant opportunity for improvement is indicated by feedback on ‘patient fears about burdening their family with their increasing physical or emotional needs’.^31^ Another significant opportunity for improvement arises from patient feedback about ‘the lack of a clear plan of care and health services available at the time of hospital discharge’.^32^ Here, the opportunity is to develop an adequate, individualized plan of care following hospital discharge. Further opportunities for improvement pointed out by the study are to provide effective pain and symptom relief as well as to promote open and honest communication among patients, families, and care providers (for example, regarding the trajectory of the disease, risks and benefits of treatment options, etc.). The three aspects ranked most important by patients are: to avoid life support when there is no hope of a meaningful recovery; to have doctors communicate information; and to avoid being a burden on their family.

### d. Chronic Obstructive Pulmonary Disease

Rocker and colleagues surveyed 118 Canadian patients with advanced chronic obstructive pulmonary disease (COPD) to elicit their perspectives about end-of-life care.^33^ They point out that little is known about what quality end-of-life care entails from the perspectives of patients with COPD.

The results show that patients with COPD consider these elements most important:

- Not to be kept on life support when there is little hope for a meaningful recovery (54.9%).
- To have relief of symptoms (46.6%).
- To have an adequate plan of care and health services available upon discharge from the hospital (40.0%).
- To have trust and confidence in physicians (39.7%).
- Not to be a physical or emotional burden on their family (39.6%).

With respect to patients' current care, an important finding is that less than one-third of patients: were completely satisfied with the adequacy of information they received about their disease (including the risks and benefits of treatment options); had confidence in their physicians; and knew which physician was in charge of their care. This finding strongly indicates areas for improvement.

A qualitative study of Saskatchewan intensive care unit (ICU) clinicians involved in end-stage COPD management sought perspectives on obstacles to providing quality care for individuals with COPD who die within the critical-care environment.^34^ Difficulties in palliating dyspnea (breathlessness) and anxiety were associated with caregiver feelings of helplessness, empathy and fears about ‘killing the patient’. A sense of futility, concerns about ‘torturing the patient’ and questions about the patient/family's understanding of treatment pervade much of the discourse about caring for people with advanced COPD in the ICU. The need to prioritize care to the most unstable ICU patients meant that patients with COPD discussed in the study did not always receive the attention that clinicians thought they should ideally have.

### e. Disability

There is a dearth of empirical literature describing end of life care, palliative care and attitudes towards assisted suicide and euthanasia concerning disabled populations in Canada. It is fair to say that there is no consensus among this group. Some disability activists have raised concerns that more permissive legislation will have a negative impact on such groups, many of whom have suffered from stigma, bias and marginalization. Furthermore, prevailing attitudes towards disability engrain beliefs that consider such lives undesirable and erode sufficient resistance to public policies that could hasten death.^35^ Others, however, argue that persons living with disabilities should have their autonomy respected (historically, there have been significant violations of their autonomy) and that such respect includes respecting their wishes in regard to assisted suicide and euthanasia. Arguments against a permissive regime, they argue, disrespect their capacity for self-determination.

Stienstra and Chochinov note the comparative neglect of the special considerations raised by disability in the field of palliative care and propose a vulnerability model of palliative care to incorporate the unique features of disability in palliative care.^36^

## 4. DEMOGRAPHIC TRANSITION IN CANADA

### a. Aging

With the current demographic transition and increasingly effective medical therapy, individuals are living longer, but are also accumulating more chronic diseases. This is particularly true for people over the age of 65. This group constitutes the fastest growing segment of the Canadian population, and consumes the largest amount of healthcare resources, specifically in terms of physician visits, home care resources, and pharmacotherapy. Chronic disease management itself has been identified as an important emerging issue in health care;^37^ 81 per cent of community-dwelling seniors report having a chronic health condition.^38^

Policy documents project that the peak of the so-called Baby Boom generation will surpass age 65 sometime between 2015 and 2035, and this population-aging effect – resulting from increases in life expectancy and past cycles in fertility – will place pressure on the Canadian healthcare system. The pressure on the health care system stems from the substantial increase in healthcare needs and expenditures once individuals reach age 65.^39^ Seniors (people aged 65 and older) represent 12.7% of the population and account for roughly 30% ($36.3 billion) of the total economic health care burden. This population has the highest hospital care expenditures at $10.2 billion. Seniors account for 22.8% ($2.7 billion) of physician care expenditures, 20.2% ($2.5 billion) of drug expenditures, and 33% ($11.0 billion) of mortality costs.

Greater reporting of long-term disability costs and the greater number of elderly people with chronic conditions account for the large difference between long-term and short-term disability costs. Leading factors responsible for long-term disability are musculoskeletal conditions, followed by cardiovascular conditions, nervous system conditions, and injuries. Seniors alone account for over 33% of the long-term disability for cardiovascular diseases. With respect to prescription drug expenditures, seniors account for 54.3% ($963 million) of expenditures for cardiovascular diseases, followed by endocrine and related diseases (34.8%), musculoskeletal diseases (25.8%), digestive diseases (25.4%), respiratory diseases (15.4%), and mental disorders (13.4%). Seniors account for almost 50% ($413 million) of Canadian prescription drug expenditures for hypertension and arthritis ($129 million), and for nearly 66% for ischemic heart disease ($331 million).^40^

These dramatic trends have also exposed limitations in informal care and community care models and – with attendant wait times in the health care system and the decreasing availability of primary care physicians – have led some to question who will care for the oldest Canadians.^41^ There are substantial issues regarding a profound lack of understanding of the death and dying experiences of older individuals. However, health status becomes more heterogeneous in late-life. As Hallberg notes in a critical literature review, empirical studies on the perspectives of older people – particularly the oldest age groups – are few.^42^ A wide range of common themes emerged in this review including readiness to talk about death and dying, conceptions of death, after-death and dying, and the impact on (and of) those close by. The latter topic had both negative and positive connotations for respondents, especially related to balancing closeness with being a burden and with dependency. Other topics included death anxiety and its possible antecedents, the fine line between natural sadness and suffering from depression, and worry about the end-of-life phase.

### b. Diversity

As well as an aging population, Canada is becoming a more culturally diverse nation with increasing heterogeneity with respect to ethnic origin, languages, health practices, and core beliefs. A March 2010 Statistics Canada report indicated that by 2031, between 25% and 28% of the population could be foreign-born; 55% of this population is expected to be born in Asia; and between 29% and 32% of the population could belong to a visible minority group.^43^ Such diversity reinforces the need for culturally sensitive end-of-life care and, ultimately, a deeper understanding of the underlying values and preferences of minority groups related to death and dying. For example, research has shown that some Chinese Canadians – a large and diverse minority group whose perspectives on health are often influenced by Confucianism, Buddhism and Taoism – hold negative feelings toward advance directives, believing that it is unrealistic or even unwise to make predictions about one's future health when not currently facing health issues.^44^ It is commonly assumed that in Chinese culture the topic of death and dying is largely avoided, or that Chinese families prefer to conceal the prognosis from a dying relative to avoid causing him or her harm (or vice versa).^45^ By contrast, Feser and Bon Bernard studied a group of Chinese elders in Calgary and found that, contrary to cultural stereotypes, many Chinese respondents who were educated about palliative care want to be informed about illness.^46^

The Canadian South Asian population is another large and growing minority group whose beliefs about death and dying – as well as preferences about end-of-life care – stem from different religious faiths including Islam, Hinduism and Sikhism. In a Health Canada-sponsored study on advance-care planning led by Con, respondents from South Asia viewed terminal illness as ‘God's wish’, and so they did not want to discuss or plan for their death.^47^ They also believed that attempting to predict one's future and end of life would ultimately draw it nearer, which Con suggests points to the need to ask culturally sensitive questions about end of life without explicitly revealing that their purpose is that of advance-care planning.^48^ Further research is needed on cross-cultural perspectives to gain a clearer and more in-depth understanding of how health practitioners can provide end-of-life care that is culturally appropriate and does not inadvertently impose dominant Western values regarding death and dying.^49^

### c. First Nations

First Nations' perspectives on end-of-life care and related policies require special consideration in light of historical disadvantage and injustice. Kelly and Minty conducted a literature review of end-of-life issues in the care of Aboriginal people.^50^ They highlighted the sparseness of rigorous studies in this field and noted that many traditional Aboriginal perspectives differ from the viewpoints of other Canadians. Some elderly Aboriginals, for example, believe that truth-telling about diagnosis may be dangerous to one's health, which contrasts with mainstream Canadian values, and further creates a challenging communication barrier at the end of life.^51^ There is also a diversity of beliefs between and within Aboriginal communities, owing to differences of ‘traditional, acculturated or religious perspectives’.^52^

Currently, many Aboriginal people living in remote communities are transported to large urban centres to die, despite a study showing that the majority of Aboriginals interviewed would prefer to die at home, in the company of friends, family and their culture.^53^ It is crucial that non-Aboriginal Canadians seek to understand better the unique and diverse preferences and values of Aboriginal people toward the end of life so that their interests are better served. Ellerby and others outlined important guidelines for caring for Aboriginal people, including: respecting the individual; practising conscious communication; using interpreters; involving the family; recognizing alternatives to truth-telling; practising non-interference; and allowing for Aboriginal medicine.^54^

## 5. ADVANCE DIRECTIVES AND SUBSTITUTE DECISION-MAKING

Advance directives are one form of advance care planning. They permit competent individuals to state their preferences for end-of-life care at a time in the future when they are no longer competent or able to speak for themselves. Advance directives take many forms. They can be instruction directives or proxy directives. *Instruction directives* contain information about what decisions should be made or how (e.g. about values, goals of therapy and preferences for care in a variety of clinical scenarios), whereas *proxy directives* indicate the individual(s) who will make decisions on behalf of the patient should the patient lose competence. Advance directives statutes are found in almost all jurisdictions in Canada, though there are variations between provinces and territories with respect to the processes to be followed. Details on the legal status of advance directives and substitute decision-making are presented in Chapter Two.

Studies consistently show that, although competent adults wish to be involved in making decisions about their health care, so that their preferences can be respected, and although they trust family members and others to be substitute decision-makers, they fail to complete advance directives or communicate their preferences for end-of-life care to family members or significant others.

In its 2010 draft framework for advance care planning,^55^ the Canadian Hospice and Palliative Care Association noted that the Canadian public supports advance care planning but a relatively small number of Canadians actually engage in it. A poll conducted by the Association in 2004 found:

- Eight in ten Canadians agree that people should start planning for end of life when they are healthy.
- 70% of Canadians surveyed have not prepared a living will.
- 47% of Canadians have not designated a substitute decision-maker to make healthcare decisions for them if they are unable.
- Fewer than 44% Canadians have discussed end-of-life care with a family member.
- Although Canadians feel that end-of life care is an important discussion to have with a physician, only 9% have done so.

Martin, Emanuel, and Singer, in the November 2000 volume of the medical journal *The Lancet,* describe a patient-centred approach to advance-care planning.^56^ This includes specification of the goal of advance-care planning and the role of advance directives as an assisting tool (and not the central defining feature of advance-care planning). The best form of advance directive contains both instruction and proxy directives and ensures that the people who should be involved in care planning should include not only loved ones but healthcare professionals who are involved in the care of the patient. Successful advance-care planning should be evaluated not simply in terms of completion of the paper form, but also in terms of the extent to which patients feel in control of their destinies and family members feel relief from the burdens of decision-making at the end of life.

Research also indicates that advance directives, even if completed, are often not followed. For example, a study examining nurses by Levoix, Blondeau, and Godin shows that the knowledge of the patient's wishes has a significant effect on nurses' choice of level of care in the absence of a living will.^57^ 59% of nurses would choose the more intensive level of care that would result in potentially over-aggressive therapy, whereas, in the presence of a living will, only 31% would choose this level of aggressive care. However, it is important to note that 31% would still choose the more aggressive course even in the presence of an advance directive instructing a contrary course.

Many studies have been done to improve the implementation and uptake of advance directives. Two systematic reviews have been completed. Bravo, Dubois and Wagneur reviewed evidence regarding the effectiveness of interventions in promoting advance directives for health care as well as research.^58^ This review of 55 studies was conducted because of the uncertainty around what works best and in what patient populations. Most groups of subjects were educated in a single session led by one health care professional. The largest set of single-arm studies revealed an overall advance directive completion rate of 45.6%. Multi-variable analyses identified that providing oral information over multiple sessions is the most successful intervention; this indicates the importance of educational interventions in increasing the use of advance directives.

Patel, Sinuff and Cook conducted a systematic review of educational advance-care planning interventions directed at patients without terminal illness to determine their influence on the completion rate of advance directives.^59^ The review was based on nine randomized controlled trials, involving 3026 patients, and tested a variety of interventions delivered by healthcare professionals, designed to educate patients about advance directives. The effectiveness of these interventions is both clinically important and statistically significant, as reflected by the 3.71 (1.46; 9.40) overall odds ratio for the completion of an advance directive. The authors of the study conclude that advance directive completion rates – documenting patient preferences for end-of-life care – may be increased by simple patient-directed educational interventions.

Molloy and colleagues, in a randomized controlled trial on implementing an advance directive, demonstrated an increase in completed advance directives by participants when they are educated about them.^60^ They also demonstrated statistically significant reductions in health costs with fewer hospitalizations per resident and less resource-use per patient in the intervention than in the control group. They also documented that completion of an advance directive is not associated with increased mortality.

Health care providers in Canada are generally supportive of the use of advance directives. Kelner and colleagues interviewed 20 physicians and 20 nurses at a major Canadian teaching hospital to elicit their views and experiences related to the use of advance directives in clinical care.^61^ All but one of the physicians and all of the nurses supported the use of advance directives – both instruction and proxy directives. The participants said that advance directives are helpful for: resolving disagreements between patients and their families about treatment options; making patients more physically and psychologically comfortable during the process of dying; and opening up communication and trust among patients, their families and health care professionals. The participants, however, raised the following concerns about the use of advance directives: the lack of clarity in some patients' instructions (such as when the advance directive is not clearly worded or is too vague); the possible interference with a practitioner's clinical judgement (such as when there is a conflict between a patient's instructions and the practitioner's clinical judgement); the adequacy and appropriateness of patients' information about their circumstances (such as when it appears that the patient did not have sufficient information about the clinical situation and options to make an informed decision about future treatment); and the type of intervention requested by patients (for example, if the patient indicates that active measures be taken to end his/her life, the participants felt that such a request could not be honoured).

Hughes and colleagues explored Ontario family physicians' attitudes, experience, and knowledge related to advance directives in a 1992 study.^62^ The results show that 86% of the physicians surveyed support the use of advance directives, but only 19% had ever discussed them with more than 10 patients. 80% of physicians surveyed had never used advance directives in managing an incompetent patient, and of the physicians who had done so, more than half report that they had not always followed the directions contained in the directive.

Blondeau and colleagues assessed the beliefs that influence nurses' intention to respect or not respect an advance directive document.^63^ 306 nurses who work either in a long-term care centre or in a hospital centre offering general and specialized care completed a multi-statement questionnaire. The results show that nurses have a strong intention to comply with advance directives written by patients. Blondeau and colleagues also studied the concordance between health care providers and patients with respect to advance directives.^64^ In a survey of 921 participants (123 patients, 167 physicians, 340 nurses and 291 administrators of health care institutions) they noted that the general attitude of each population is favourable to the use of advance directives. However, physicians' attitudes towards advance directives are shown to be less positive than patients' attitudes.

A recent US American study examined the prevalence of advance directives and assessed the concordance between patient preferences as stated in the directive and the actual care the patient received.^65^ Patients who had advance directives were more likely to want limited health care intervention (92.7%) or palliative care (96.2%). The study found that 83.2% of subjects who requested limited care and 97.1% of subjects who requested comfort care received care consistent with their preferences. As noted above, such data is not available for Canada, but this study does suggest, contrary to recent scepticism, that the use of advance directives may be an effective means of assuring that patient preferences are met.

## 6. SEDATION PRACTICES

The use of sedation at the end of life has recently come under considerable scrutiny. A number of terms are used in the literature –*palliative sedation*, *terminal sedation*, *deep continuous sedation*– to describe the varied practices. While this report provides precise definitions for palliative and terminal sedation in the terminology section above, there is inconsistent use of descriptors of sedation practices in the literature. Therefore, in this section they are referred to generically as sedation practices.

There is scant empirical research on sedation practices in Canada. In one study, Blondeau and colleagues evaluated the influence of prognosis and suffering on clinicians' attitudes to the use of sedation.^66^ The results of this survey – 124 clinicians working in palliative care environments in Quebec were included – demonstrate that ‘the type of suffering influences a subject's attitude to end-of-life sedation.’ Physical suffering was associated with respondents being in favour of the use of sedation. However, clinicians were not in favour of the use of sedation for existential suffering. The authors note ‘that health professionals are uncomfortable when confronted with their patients' existential suffering.’^67^

Blondeau and colleagues, in another study, assessed the attitudes of a small cohort of Quebec palliative care physicians.^68^ The authors noted that there are few guidelines in Quebec on end-of-life sedation. Furthermore, the normative justification for using sedation is not explicit. They found that Quebec physicians undertand sedation as a measure to relieve the patient's suffering; not to hasten death. Most physicians viewed euthanasia and sedation as distinctly different practices. The motivation to proceed with sedation was strongest when treating refractory physical symptoms. Physicians were divided on whether emotional or existential suffering were adequate reasons to proceed with sedation, and some were strongly opposed to proceeding on such a basis. The authors suggest the need for research aimed at developing a clear definition of existential suffering and the ethical basis of using sedation to treat it.

A small study of Manitoba palliative care nurses uses the metaphor of a quagmire to describe the decision-making landscape; it indicates that sedation raises complex and difficult moral challenges.^69^ Hawyrluk and colleagues offer a set of guidelines for the use of analgesia and sedation on dying ICU patients.^70^ The guidelines were developed to help distinguish palliative care from euthanasia as well as to reduce the risks of under-treatment, such as: pain and suffering; the risk of over-treatment; and prosecution for euthanasia. Their guidelines indicate a general consensus among those surveyed with regards to:

- The role of palliative care in the ICU (for example, to relieve pain and suffering of each patient, not just dying patients).
- The management of pain and suffering (for example, when to use pharmacological versus non-pharmacological methods).
- Ways to improve palliative care in the ICU (for example, a major problem is the difficulty in assessing the patient's pain and suffering, so open discussions with all members of the health care team and family, improvements in education and training, and research are needed).

Berger has recently shown that there is a lack of consensus in published guidelines on the use of sedation at the end of life.^71^ While all guidelines agree that sedation is permitted in the context of a terminal condition with refractory and intolerable symptoms, they differ with respect to the length of life-expectancy and the acceptability of existential suffering as a basis for provision of sedation. He argues for greater clarity and consistency in guidance documents on the use of sedation.

## 7. PAEDIATRIC END OF LIFE CARE

Paediatric palliative care needs remain relatively understudied in a Canadian context. Widger and colleagues conducted a multi-centre study to obtain knowledge about Canadian children who received care from theeight dedicated paediatric palliative care programs in Canada during 2002.^72^ The results showed:

- 48.6% of the patients were younger than five years old, and about half of these were younger than one year old.
- Diagnoses were wide-ranging, but the most common included disorders of the nervous system (39.1%), malignancies (22.1%), and conditions arising in the peri-natal period just before and after birth or congenital anomalies (22.1%).
- In terms of the location of death, a large number of children (43.9%) died at home. Centres with more comprehensive home care services reported higher percentages of home deaths.
- The most significant finding was the small percentage of children who receive care from a paediatric palliative care program in Canada. Using various methods, the authors estimated that between only 5% and 12% of the children who might benefit from the services of a paediatric palliative care program actually received these services.

The authors conclude that their study ‘reveals that most Canadian children who may benefit are not referred to existing paediatric palliative care services or do not have access to services because of geographic availability.’ They rightly note that the need for further research in this area is a high priority.

Steele and colleagues, in a Canadian study, identified research priorities considered highly important for advancing knowledge in paediatric end-of-life care among researchers and frontline palliative care clinicians.^73^ The four most significant research questions identified were the following:

- What matters most for patients and parents receiving paediatric palliative services?
- What are the best practice standards in pain and symptom management?
- What bereavement needs do the families in paediatric palliative care have?
- What are effective strategies to alleviate suffering at the end of life?

A recent US American study surveying 141 parents of children who died of cancer estimates the frequency of discussions about hastening death.^74^ The authors sought data both to describe the level of parental support for hastening death and intensive symptom management, and to determine whether such discussions and support were influenced by the level of children's pain. The results showed that:

- 13% of parents considered requesting hastened death for their child at his or her end of life and 9% actually discussed hastening death.
- Consideration of hastening death generally increased as the child's suffering increased.
- 34% of parents reported that they would have considered hastening their child's death if the child had been in uncontrollable pain, while 15% or less would consider hastening death for non-physical suffering.
- The results also showed that, in response to vignettes portraying children with end-stage cancer, 50% of parents supported hastening death, while 94% supported intensive pain management.

The study concludes that a child's level of pain is a major factor in parents' consideration of hastening death. The authors suggest that to ease parents' consideration of hastening death, it is important to point out the sources of suffering, and to explain the effectiveness of intensive symptom management as an alternative approach.

## 8. ATTITUDES OF CANADIANS TOWARD VOLUNTARY EUTHANASIA AND ASSISTED SUICIDE

### a. General Public

Recent surveys show significant public support for both voluntary euthanasia and assisted suicide. In a 2010 Angus Reid survey of a national sample of 1,003 Canadians, a large percentage (67%) of respondents was in favour of legalizing voluntary euthanasia.^75^ In this survey, more than 85% of Canadians said they believe legalizing voluntary euthanasia can provide people who are suffering with a chance to ease their pain, and 76% said they believe doing so would establish clearer guidelines for physicians dealing with end-of-life decisions. Additionally, about two-thirds (66%) of Canadians thought legalizing voluntary euthanasia would not send the message that the lives of sick or disabled people are less valuable. Overall, roughly half (41%) of respondents said they believe that people who assist a person to commit suicide should be free from prosecution.

Strong support for legalizing voluntary euthanasia was also found in a 2009 Angus Reid national survey of 1,006 Canadians; nearly three-quarters (71%) of respondents favoured such legalization.^76^ Similar results were obtained by a 2007 national survey by Ipsos Reid of 1,005 Canadians; this survey found that 76% of respondents support the right to die for patients suffering from an incurable disease.^77^ People in Quebec showed the strongest support (87%), while people in Alberta showed the least support (66%).

The high level of public support for legalizing voluntary euthanasia and assisted suicide under certain circumstances has scarcely changed over the past decade and a half. In a 1995 cross-sectional survey of 2,019 Canadians, Singer and colleagues found that a majority of respondents support legalizing voluntary euthanasia (66%) and assisted suicide (58%) if the person is competent and unlikely to recover from his or her illness.^78^ Support is only slightly less (58%) for legalizing voluntary euthanasia if the family of an incompetent person who is unlikely to recover (but whose wishes about end of life are unknown) request euthanasia for that person. By contrast, most respondents disapprove of a law allowing voluntary euthanasia (78%) or assisted suicide (79%) if the person is incompetent but likely to recover.

These results can be compared to those of a 1994 survey by Genuis et al. of 356 people in Edmonton, which revealed a high degree of public support (65%) for voluntary euthanasia for elderly, terminally ill people in severe pain, but significant opposition to this practice for people in other circumstances.^79^ That is, 65% oppose voluntary euthanasia for elderly disabled people who say they feel like a burden on their family, 83% oppose voluntary euthanasia for elderly disabled people who say they feel lonely and have only minor physical ailments, and 75% oppose voluntary euthanasia for people with chronic depression resistant to treatment. In this survey, although the public was generally supportive of voluntary euthanasia for terminally ill patients, a roughly equal number of respondents (63%) said they believe that legalizing this practice for such patients would lead to euthanasia for several other, unsupported reasons.

It can be inferred, based on the different surveys described above, that the majority of the Canadian public would support legislation permitting voluntary euthanasia and assisted suicide for people suffering from an incurable physical illness.

### b. Health Care Professionals

Recent data on the attitudes of Canadian health care professionals toward euthanasia and assisted suicide are lacking, but opinion surveys published in the 1990s, of which there were only a few, are nonetheless informative. In a 1996 survey of more than 1700 physicians from across Canada, roughly a quarter of respondents indicated their willingness to practise voluntary euthanasia (24%) or assisted suicide (23%) if it were legal to do so, while the majority (55%) said they would not practise either.^80^ Overall, these physicians were more willing to refer patients to a colleague for voluntary euthanasia (44%) and assisted suicide (41%) than to carry out such acts personally.

Suarez-Almazor and colleagues obtained similar results in a 1997 survey in which, of 179 physicians surveyed in Edmonton, only a small minority of respondents stated that they would be willing to perform voluntary euthanasia or assisted suicide (14% to 27% depending on the end-of-life circumstances) if such practices were allowed by law, while most indicated that they would be unwilling to do so (68% to 75% depending on the end-of life circumstances).^81^ In this survey, roughly 60% to 80% of all physicians were opposed to legalizing voluntary euthanasia or assisted suicide.

In contrast to physicians, nurses and social workers are significantly more supportive of voluntary euthanasia and assisted suicide, according to two surveys. In 1998, Young and Ogden surveyed 160 nurses and found that nearly three-quarters (73%) of respondents said they believe the law should be amended to permit physicians to practice voluntary euthanasia and assisted suicide.^82^ As well, more than half (53%) thought that nurses, too, should be allowed to practice such acts. In the same year, these authors surveyed 527 social workers in British Columbia and found that most respondents believed voluntary euthanasia (75.9%) and assisted suicide (78.2%) should be legal under certain conditions.^83^ 21% of the social workers had been consulted by a patient about these acts; six social workers reported assisting in the death of a patient through voluntary euthanasia.

A 2009 poll of 2,025 medical specialists in the Quebec found that 75% said they were ‘certainly’ or ‘probably’ in favour of legalizing euthanasia, as long as the practice were regulated.^84^

In October 2009, the College of Physicians in Quebec released a report entitled *Physicians, Appropriate Care and the Debate on Euthanasia*, calling for an open discussion on the question of euthanasia in the context of end-of-life care. The report states that if euthanasia is to be permitted it should be conducted in the context of care and considered a medical act.^85^

### c. Patients

Patients are those most directly affected by laws governing voluntary euthanasia and assisted suicide, so their attitudes toward these practices warrant careful consideration. In 2007, Wilson and others conducted a study of 238 terminally ill cancer patients in palliative care and found that a majority (62.8%) of such patients support legalizing voluntary euthanasia and assisted suicide.^86^ Further, about 40% of patients could foresee possibly making a request for physician-assisted suicide in the future. Physical and psychological suffering, extreme loss of functions, dependence on others, hopelessness, or being a burden on family members were cited by patients as factors that might motivate them to make such a request. As well, about 6% of patients reported that they would actually request physician-assisted suicide in their present circumstances if it were legally accessible. The study's authors determined that these patients based their desire to end their lives on a combination of factors ranging from a feeling of futility about their deteriorating and functionally-limited state of health, to physical and psychological suffering, to feeling like a burden on others or a drain on health care resources.

This study's findings match those of an earlier study, conducted by Wilson and colleagues in 2000, of 70 terminally ill cancer patients in which almost two-thirds (64%) of respondents said they thought both voluntary euthanasia and physician-assisted suicide are acceptable and should be legalized.^87^ Whereas these patients cited pain (43%) and a person's right to choose (43%) as the principal reasons for legalizing such practices, those patients who were against legalization (21%) cited religious beliefs (50%) and moral objections (38%) as their chief concerns. In this study, over half of respondents (58%) indicated that, if legally available, they might request euthanasia or assisted suicide in the future, citing possible uncontrollable pain (47%) and other physical symptoms (34%) as the main motivating factors. Overall, 8 of the 70 patients said they would actually have requested a physician-hastened death at the time the study was done had it been legal. Perhaps unexpectedly, Wilson and others found that pain was cited by only one of these eight patients as a contributing reason; instead, half or more of these patients based their desire to end their lives on factors such as their recognition and acceptance of their terminal illness, having a diminished quality of life, their right to exercise control over their deaths, or their belief that voluntary euthanasia or assisted suicide would be an easier way to die than what they were currently facing.

The findings of the two studies described above are similar to those obtained in Achille and Ogloff's 2003 study of 44 terminally ill patients with Amyotrophic Lateral Sclerosis (ALS).^88^ Of such patients 70% thought that assisted suicide was morally acceptable and 60% favoured its legalization. Those who supported its legalization emphasized a person's right to self-determination, the preservation of one's dignity, and freedom from dependence or being a burden on others. Those who opposed its legalization feared it might lead to involuntary euthanasia, said they believed that death should occur naturally, and were also more likely to be heavily involved in religious practice. Additionally, the majority (60%) of patients could foresee circumstances that would make them consider assisted suicide in the future if it were legally available, and three of the 44 patients indicated that since their diagnosis, they would already have asked for assisted suicide if it had been legal.

Finally, Lavery and colleagues conducted a study in 2001 of 32 patients with HIV or AIDS and, using a more interpretive approach to analysis, determined that three main factors led to many of the respondents' desire for voluntary euthanasia or assisted suicide.^89^ The first was *disintegration*, a process during which the patients experience a loss of functions and increased symptoms associated with their disease. The second was *loss of community*, a process by which the patients' loss of mobility and exclusion or alienation by others results in difficulty maintaining, or the erosion of, close personal relationships. These two factors together led to the third factor, the patients' *loss of self*, that is, the feeling that their fundamental nature had been or is at risk of being completely worn away.

## 9. INTERNATIONAL COMPARISONS

In Canada, the level of public support for legalizing voluntary euthanasia and assisted suicide is comparable to that in the United Kingdom, but markedly higher than that in the United States, according to a 2009 Angus Reid survey of national samples.^90^ In this survey, Canadians demonstrated slightly less support (71%) than that of Britons (77%) and nearly twice that of US Americans (45%). By a measure of public support, Canada appears to be roughly equal to The Netherlands,^91^ where both voluntary euthanasia and physician-assisted suicide are carried out legally. Further, in a 2006 study, Rietjens and others found a clear link between the Dutch public's support for voluntary euthanasia and a number of features it considers important for a ‘good death’.^92^ These include an influence on the dying process through personal decisions about treatment and the time of death, avoiding being a burden on relatives, and preventing severe suffering and loss of dignity.

When compared with the general public, physicians not only in Canada (as indicated above), but also in the United States^93^ and the United Kingdom,^94^,^95^ are significantly less supportive of legalizing voluntary euthanasia or assisted suicide, and many are opposed. Although the reasons for such opposition have not been sufficiently explored among Canadian physicians, studies of American physicians^96^ and British physicians^97^,^98^ suggest a strong association between opposition to legalizing physician-assisted suicide and voluntary euthanasia and religious belief. Further, in surveys of physicians in the United Kingdom, opponents were also more likely to be palliative care specialists,^99^ or those caring for the dying.^100^ The views of physicians in The Netherlands contrast with those of physicians in Canada, the United States, and the United Kingdom, in that a large majority – 84% – of Dutch physicians support physician-assisted suicide and/or voluntary euthanasia.^101^

The attitudes of patients in Canada toward voluntary euthanasia and assisted suicide (as described above) are comparable to those in the United States, the United Kingdom, and The Netherlands. Most notable is that patients in all of these countries cite similar reasons for considering or asking for physician-assisted suicide and voluntary euthanasia. In 2009, Ganzani and colleagues studied 56 patients from the state of Oregon (where eligible patients receive help legally from physicians to commit suicide), who had requested physician-assisted suicide or had contacted a physician-assisted suicide advocacy organization.^102^ The authors found that the main reasons for such requests were the patients' desire to influence the circumstances of their death, loss of independence, worries about future pain, poor quality of life, and inability to care for themselves. Similarly, in 2006, Chapple and others interviewed 18 terminally ill patients in the United Kingdom, and found that those who support legalizing voluntary euthanasia or assisted suicide emphasized concerns about future pain, fear of indignity, loss of control, and cognitive impairment.^103^ Finally, in a 2009 study by Pasman and colleagues, Dutch patients who had formally requested aid in dying said that their ‘unbearable suffering’ (which is one of several conditions for receiving euthanasia in The Netherlands) consisted of physical elements, including pain, but, more often, non-physical elements, including dependence, an inability to lead a normal daily life, and mental suffering over steady deterioration.^104^

The results of these studies suggest that these patients, generally, like Canadian patients, are interested in or request euthanasia or assisted suicide not because of any singular reason; instead, their motivation arises from a complex combination of physical, psychosocial, and existential suffering – importantly, this is a type of suffering that has objective as well as subjective elements.

## 10. CONCLUSIONS

In this review of the literature, the Panel identifies the following key features:

1. The vast majority of Canadians die in institutions in their old age.
2. The Canadian population is rapidly changing – it is becoming increasingly aged, but also more diverse.
3. Literature suggests that the attitudes and perspectives of the very old toward assisted suicide and euthanasia have not been ascertained, nor is the literature well-attuned to First Nations and the ethnically and culturally diverse populations now found in Canada. Hearing these voices is integral to an informed debate on end-of-life care.
4. Advance-care planning still remains a topic not sufficiently discussed by individuals, their families and their health care providers, with the vast majority of Canadians having neither proxy nor instruction directives. The absence of explicit dialogue between patients and health care providers is of concern to the Panel.
5. The use of sedation as a modality of care at the end of life appears to be increasing without concurrent increasing clarity on the appropriateness of various kinds of sedation in various circumstances. There is a pressing need for a set of national consensus guidelines.
6. A significant majority of the Canadian population appears to support a more permissive legislative framework for voluntary euthanasia and assisted suicide.

One final note must be made with respect to this literature review. The literature seems to cluster in the mid-1990s to early 2000s, around the time of the last Senate Sub-Committee on Euthanasia and End of Life. The Panel sees the need for much of this research to be updated with a greater focus on public engagement strategies that would provide the opportunity for deeper deliberations and more nuanced discussion than can be found in many of the studies conducted to date.

# Sub article: CHAPTER TWO: THE LEGAL LANDSCAPE

**Published:** 2011-11

## 1. INTRODUCTION

This chapter provides an overview of end-of-life law in Canada. As with the previous chapter and, given the interplay between the categories of assisted dying, the Panel recognizes the need to review the full spectrum of end of life care. In this chapter, the Panel describes the legal status of the withholding and withdrawal of potentially life-sustaining treatment, advance directives, the provision of potentially life-shortening symptom relief, terminal sedation, assisted suicide and voluntary euthanasia. The focus here is not on whether the law is defensible (that will come later), but rather on what the law is with all its inherent clarity, confusion, and controversy.

## 2. WITHHOLDING AND WITHDRAWAL OF POTENTIALLY LIFE-SUSTAINING TREATMENT

### a. Relatively Clear and Uncontroversial

A 78-year-old man is admitted to hospital after a fall at home. Following x-rays to identify the nature of the injuries caused by his fall, his physicians diagnose advanced lung cancer. They discuss the various treatment options with him including chemotherapy followed by radiation therapy, surgery to remove some of the tumours and relieve pressure, and doing nothing apart from managing pain and any other discomfort that might arise. He understands that the chemotherapy has a 70% chance of extending his life expectancy by two years beyond that which would be expected without any treatment. He refuses treatment (in particular citing the physical burden of chemotherapy) and indicates that he wants to go home. Is it legal for the physicians to respect this refusal, knowing that treatment could extend his life?

At first glance it might appear that refusals of treatment are not permissible under Canadian law. Section 215 of the *Criminal Code of Canada* establishes a duty to provide the necessaries of life; these have been found to include medical treatment.^105^ Section 215(2) establishes that it is an offence to fail to meet the duty to provide necessaries of life ‘without lawful excuse’ if such failure ‘endangers the life of the person to whom the duty is owed or causes or is likely to cause the health of that person to be injured permanently.^106^ Section 217 establishes a duty to continue an undertaken act.^107^ Section 219 establishes that ‘(1) [E]very one is criminally negligent who (a) in doing anything, or (b) in omitting to do anything that it is his duty to do, shows wanton or reckless disregard for the lives or safety of other persons.’^108^ Withholding or withdrawal of potentially life-sustaining treatment while showing wanton or reckless disregard for the life or safety of the person from whom treatment is being withheld or withdrawn could, therefore, constitute criminal negligence.

However, these sections of the *Criminal Code* must be read in light of the Supreme Court of Canada's statements about a common law right to refuse treatment. For example:

Canadian courts have recognized a common law right of patients to refuse consent to medical treatment, or to demand that treatment, once commenced, be withdrawn or discontinued. This right has been specifically recognized to exist even if the withdrawal from or refusal of treatment may result in death.^109^

The Supreme Court of Canada's position is grounded in a deep commitment to the value of autonomy and the consequential belief that competent individuals should be free to chart their own course and their wishes should be respected with a few exceptions (such as where this would cause harm to others).

Given that conduct consistent with the common law right set out above would probably not be found to constitute wanton or reckless disregard for the lives or safety of others (assuming the conduct is not negligent in some other way – and might be found to constitute ‘lawful excuse’), it can be concluded that, under Canadian law, health care professionals must respect refusals of treatment from competent adults.

A 4-year-old girl is taken to hospital following a car accident. She has massive internal bleeding and has sustained severe head trauma. The health care team stabilizes her and she is admitted to the ICU on a ventilator. She is treated for a period of time but ultimately diagnosed as being in a persistent vegetative state. While she is no longer on a ventilator, she requires artificial hydration and nutrition. Her parents come to the healthcare team and ask that all treatment (including the hydration and nutrition) be stopped. Is it legal for the team to do so?

Canadian courts and statutes have also clarified that health care professionals must respect refusals made on behalf of incompetent persons, without valid instruction directives, by their legally authorized substitute decision-makers.^110^ Substitute decision-makers are charged with making decisions according to prior competent wishes or, where applicable wishes are or could not be known, according to what the substitute decision-maker believes to be in the person's best interests. Respecting refusals based on the former standard is grounded in the commitment to the value of autonomy (understood as including bodily integrity)^111^ described above. Respecting refusals based on the latter standard is grounded in the belief that it can be in a person's best interests to be allowed to die.^112^

It must also be noted here that the law draws no distinction between withholding and withdrawal.^113^ Nor does it treat artificial hydration and nutrition any differently than other technological or pharmaceutical interventions (for example, mechanical ventilation or antibiotics).^114^ It does not restrict refusals to situations involving terminal illness or imminent death.^115^

It can therefore be concluded that there is considerable clarity and very little controversy concerning the law as it relates to withholding and withdrawal of potentially life-sustaining treatment from competent adults, or on behalf of incompetent persons without valid advance directives by their legally authorized substitute-decision-makers. Health care providers must respect valid refusals. Despite this, there remains some confusion. Not all health care providers or members of the public understand their legal rights and responsibilities.^116^ As a result, as mentioned in the preceding chapter, it is possible that some legally valid refusals of treatment are not being respected and that potentially scarce medical resources are being wasted on care not consented to by patients or their legally authorized substitute decision-makers.

A 64-year old woman goes to her family doctor and presents him with a carefully drafted document setting out which treatments she would like to receive or not at a point in the future when she is no longer competent to make decisions on her own behalf. For example, she states that if she were to be in a persistent vegetative state, she would not want antibiotics for infection or artificial hydration or nutrition. She asks him whether he will be willing and legally able to respect her wishes.

Some courts embraced advance directives even before advance directives legislation was passed. For example, the Ontario Court of Appeal has held that:

A patient, in anticipation of circumstances wherein he or she may be unconscious or otherwise incapacitated and thus unable to contemporaneously express his or her wishes about a particular form of medical treatment, may specify in advance his or her refusal to consent to the proposed treatment. A doctor is not free to disregard such advance instructions, even in an emergency. The patient's right to forgo treatment, in the absence of some overriding societal interest, is paramount to the doctor's obligation to provide medical care. This right must be honoured, even though the treatment may be beneficial or necessary to preserve the patient's life or health, and regardless of how ill-advised the patient's decision may appear to others.^117^

Furthermore, statutes require respect for one or both of instruction and proxy directives in almost all jurisdictions.^118^ There is, however, some variation across jurisdictions. For example, some jurisdictions do not allow minors to complete advance directives while others do.^119^ Nonetheless, the core principle that the prior-expressed wishes of previously competent adults should be respected when it comes to end of life decision-making is consistent across the jurisdictions. Thus it can be concluded that the law here is relatively clear.

That said, there have certainly been difficulties with implementation. Many people have not completed advance directives, have completed directives that do not provide clear direction, or have appointed proxies who are unlikely to actually know their past wishes.^120^ These issues have been canvassed in Chapter One and so will not be repeated here. It is worth noting that there has been debate, particularly in the philosophical literature, about the legitimacy of the core principle behind requiring respect for advance directives. There has been some rejection of the notion that an individual should be able to direct what happens to them once they become incompetent.^121^ However, the legal status of advance directives has not been the subject of significant calls for reform so the Panel has determined the legal status of advance directives to be relatively clear and uncontroversial.

### b. Less Clear and More Controversial

A 15-year-old girl is dying of leukaemia. She refuses further blood transfusions after three unsuccessful and gruelling rounds of chemotherapy. If she is able to understand and appreciate the nature and consequences of her decision (as well as the alternatives available to her), is it legal for her refusal to be respected? Must her decision be respected?

A 15-year-old girl who has been quadriplegic for five years refuses antibiotics for a simple pneumonia. If she is able to understand and appreciate the nature and consequences of the decision she is making and the alternatives available to her, is it legal to respect her refusal? Must her refusal be respected?

There remains uncertainty and controversy within Canadian law regarding withholding and withdrawal of potentially life-sustaining treatment from mature minors. Mature minors are those who, while under the age of majority,^122^ are able to understand and appreciate the nature and consequences of a decision to refuse potentially life-sustaining treatment. If such a minor refuses such treatment, must the patient's refusal be respected or only when others see his or her decision as being in his or her best interests?

To answer these questions, a number of sources must be consulted. These include the common law mature minor rule,^123^ the courts' overall jurisdiction to protect the vulnerable,^124^ provincial/territorial child and family services legislation, provincial/territorial consent legislation, and the Canadian *Charter of Rights and Freedoms*. There are also prior cases interpreting these various sources of legal authority. A review of these authorities suggests that it is legally permissible (if not required) to respect the refusal in the first case. This position, like that of withholding or withdrawal from adults, is grounded in a commitment to autonomy and the view that autonomy does not directly correlate with age and, for some, that this decision is in her best interests. In contrast, whether it is legally permissible or required to respect the refusal in the second case is much less clear. The level of confusion depends, in part, on the province or territory, as some have legislation and case law and some do not.^125^

A recent Supreme Court of Canada decision on mature minors^126^ has relieved some of the confusion resulting from conflicting decisions by provincial courts of appeal.^127^ It is clear now that, in some circumstances, mature minors' refusals may be overridden. For example, the court may override if it determines the medical decisions being made by the minor are not in the minor's best interests (with the level of maturity taken into account in the court's assessment of the minor's best interests). While this gives some clarity, there remains, unfortunately, considerable confusion. This is both because the decision is itself somewhat unclear, and because the issue continues to be complicated by the various interlocking sources of legal authority listed above.

Controversy also remains as debates continue about whether the consent of mature minors should be considered both necessary and sufficient, or as not sufficient for treatment decisions. Some believe that if the minor understands and appreciates the nature and consequences of the decision to be made (here a refusal of potentially life-sustaining treatment), then it is irrelevant how others view his or her decision.^128^ Others believe that, even if the minor understands the nature and consequences of the refusal, the refusal should only be respected if it can be seen by others (such as her parents, the health care team, or the court) to be in her best interests.^129^

### c. Very Unclear and Very Controversial

A 65-year-old man is in hospital in a persistent vegetative state. His family believes that he would have wanted, and it would be in his best interests, for him to be resuscitated should he have a cardiac arrest. The health care team says to attempt resuscitation would be futile and refuses to do it. The team is going to put a Do Not Resuscitate Order on the chart. Is it legally permissible for the team to do this?

There is a great deal of confusion and controversy regarding the legality of unilateral withholding and withdrawal of potentially life-sustaining treatment. The issue here is whether health care professionals have the legal authority to withhold or withdraw treatment against the wishes (or without the knowledge) of the patient or the patient's substitute decision-maker.

In a few cases, courts have found that a physician did have the authority to unilaterally withhold or withdraw treatment.^130^ More commonly, however, the courts have found that the issue of unilateral withholding and withdrawal is unsettled in law.^131^ In policy statements and academic literature, there are statements to the effect that health care providers do have legal authority to unilaterally withhold or withdraw treatment but there are also statements that say the law is unclear.^132^

There is extraordinary conflict over what the law on this matter should be. Many papers have been published in the ethics^133^ and medical literature;^134^ many columns of ink have been spilled in the popular press.^135^ It has been argued in the literature that, if the health care team believes requests for specific treatments by substitute decision-makers are not in the best interests of the patient, the decision to withhold or withdraw treatment should rest with the health care providers.^136^ Others maintain that the decision should rest with the substitute decision-makers, or that conflicts between the health care team and the substitute decision-makers should be resolved by society (through the courts or some form of specialized tribunals).^137^ Statements from the courts or the legislatures will be needed to resolve the confusion and controversy in this area.

## 3. POTENTIALLY LIFE-SHORTENING SYMPTOM RELIEF

### a. Somewhat Clear and Relatively Uncontroversial

A 75-year-old man is dying of stomach cancer. He is suffering greatly despite being on morphine for a week. His daughter asks the doctor to increase the morphine (frequency and dosage). The physician explains that he cannot be sure that such an increase will not in fact shorten her father's life, because of the potential for depression of her father's respiration. The man's daughter begs the doctor to increase the morphine anyway, saying that she cannot bear to see her father suffering like this and that she knows from conversations with her father that he would have chosen the risk of earlier death over ongoing pain had he been competent.

The question here is whether someone who provides potentially life-shortening symptom relief could be convicted under the *Criminal Code* with criminal negligence causing death (for example, culpable homicide).^138^ No case that is directly on point has reached the Supreme Court of Canada. However, there are some relevant comments to be found in the assisted suicide case involving Sue Rodriguez (Canada's most famous assisted suicide case about which more will be said in a later section of this chapter).^139^ For the majority in *Rodriguez*, the late Justice Sopinka wrote:

[t]he administration of drugs designed for pain control in dosages which the physician knows will hasten death constitutes active contribution to death by any standard. However, the distinction drawn here is one based upon intention – in the case of palliative care the intention is to ease pain, which has the effect of hastening death, while in the case of assisted suicide, the intention is undeniably to cause death …. In my view, distinctions based upon intent are important, and in fact form the basis of our criminal law. While factually the distinction may, at times, be difficult to draw, legally it is clear.^140^

It can be argued, on the basis of this judgment, that the provision of potentially life-shortening symptom relief is legal if the intention is to ease pain.

Despite this, much remains uncertain: How much medication is too much? Are there limits on who can be given such treatment? Must the person be terminally ill? Must suffering be physical or can it be psychological? Are there limits on when such treatment can be given? Must someone be imminently dying? Many questions such as these remain unanswered by the law. Some uncertainty is addressed through guidelines (specifically, for example, the Ontario Chief Coroner's Guidelines and the British Columbia Crown Counsel Guidelines for the exercise of prosecutorial discretion).^141^ However, these are little known, have limited scope, and are still quite vague. Many healthcare providers and members of the public are confused and, as a result, patients may not be receiving appropriate and adequate symptom relief.^142^

## 4. TERMINAL SEDATION

### a. Very Unclear and Potentially Very Controversial

A 55-year-old woman with pancreatic cancer is admitted to hospital with pain that has become unmanageable at home. Her healthcare providers attempt to control her pain through intensive analgesia. However, this is ineffective. Her family approaches the woman's physician and say that they have read about deep and continuous sedation in their newspaper and would like that for her. They say they realize it will reduce her consciousness (perhaps render her completely unconscious), but they are confident that this is what she would want were she competent. They also realize that this would mean she could only receive food and water artificially, but they refuse artificial hydration and nutrition on her behalf; they realize this will shorten her life (with artificial hydration and nutrition she is expected to live approximately three more months). Is it legal for the physician to provide the sedation and withhold the hydration and nutrition?

The legal status of each of the two elements of terminal sedation can be assessed independently but the legal status of terminal sedation can only be determined by combining them.

The first element is deep and continuous sedation. This is considered legally acceptable care for some patients; an example is a patient with terminal bone cancer who is suffering intractable pain that cannot be relieved through any other means. However, the limits on acceptability are not clear. Does it matter whether the suffering is psychological rather than physical? Does it matter if it is in response to physical vs. mental illness? The courts have not addressed either of these questions, nor are they addressed explicitly by legislation. While the general framework for addressing consent to treatment would be applied by the courts, it is not clear what conclusions would be drawn. The second element under consideration here is withholding or withdrawing artificial nutrition and hydration. As noted earlier, this is legally permissible no matter the reason, as long as a competent patient makes a free and informed refusal. When the two elements of terminal sedation are paired, the legality becomes both unclear and controversial. To put a very fine point on it: could a physician legally respect a free and informed request, from a competent person who is not imminently dying but rather has a bipolar disorder and believes that death would be preferable to living with her condition, for deep and continuous sedation in order to create the physical need for artificial hydration and nutrition and a refusal of artificial hydration and nutrition, thus ensuring that she will die within one to two weeks? Would this be treated as just another refusal of treatment case accepted in law or would it be considered a slow form of assisted suicide not accepted in law? The answers to these questions are not clear and would surely be controversial.

## 5. ASSISTED SUICIDE

### a. Very Clear and Very Controversial

A 44-year-old man has advanced Multiple Sclerosis. He is still living at home with supportive care. He is concerned his quality of life will decline to the point at which he will be entirely dependent on others to care for him and he will no longer be able to do any of the things that bring him pleasure in life. He asks his physician to write him a prescription for a barbiturate and to give him instructions for how to use the drugs prescribed to kill himself at the time in the near future when he wants to die. Is it legal for his physician to provide the prescription?

The law on assisted suicide is clear. Under section 241(b) of the *Criminal Code* it is illegal to aid or abet a person to commit suicide (although suicide is legal).^143^ The constitutionality of the *Criminal Code* prohibition on assisted suicide has been tested and, in *Rodriguez* in 1993,^144^ the Supreme Court of Canada ruled that it did not violate the *Charter*. There have been three convictions for assisted suicide after which the defendants were sent to jail.^145^ There have been at least 17 other cases that are known to have come to the attention of the authorities in which a charge was either not laid,^146^ stayed or dropped,^147^ or the defendants were found not guilty^148^ or, where convicted, given suspended or conditional sentences or probation.^149^ In one of these cases, a man took his wife to Switzerland for an assisted suicide with the help of *Dignitas*.^150^ The police investigated the case, but ultimately decided not to lay any charges. This is consistent with the principle that, barring an explicit exception in the law, you cannot be tried for things that you do in another country when they are legal in that country even if they are illegal in Canada. It is not known how many other cases of assisted suicide have come to the attention of the authorities but not the public or have simply happened without the knowledge of the police.

While the law is clear on assisted suicide, there is certainly a great deal of controversy about it. As detailed in Chapter One, some believe that the law is appropriate and no law reform is required.^151^ Others believe that assisted suicide should be permitted in some circumstances and, accordingly, the *Criminal Code* should be revised to allow assisted suicide, under a regulated regime.^152^ There have been a number of attempts to change the *Criminal Code* through bills introduced in federal parliament (one such bill was considered as recently as May 2010), but none have been successful.^153^ As mentioned earlier, in April 2011, three cases were launched in British Columbia challenging the constitutional validity of the provisions of the *Criminal Code* that prohibits assisted suicide.^154^ It remains to be seen whether any or all of them will be successful.

## 6. VOLUNTARY EUTHANASIA

### a. Very Clear and Very Controversial

A woman is suffering from advanced amyotrophic lateral sclerosis (ALS, also known as Lou Gehrig's disease). She is paralyzed from the neck down and can no longer swallow or breathe on her own She has decided that, given her condition, her life is no longer worth living and that she would rather die than continue in this irreversible state of irremediable suffering. Using a method of communication by eye blinking developed by her occupational therapist, she asks her physician to give her a lethal injection. Is it legal for him to do so?

Voluntary euthanasia, like assisted suicide, is quite clearly illegal in Canada. It is prohibited by section 229 of the *Criminal Code*. Motive is irrelevant and consent (either from the individual or a substitute decision-maker) does not provide a defence.^155^ It is unlikely that an attempt to argue the defence of necessity would succeed.^156^ First-degree murder carries with it a mandatory minimum life sentence with no possibility of parole for 25 years. Second-degree murder carries with it a mandatory minimum life sentence with no possibility of parole for ten years.^157^ Someone who commits voluntary euthanasia could be convicted of first or second degree murder. Clearly this is a serious prohibition.

That said, there have been at least eighteen cases in which charges were laid against individuals:^158^ one individual fled the country;^159^ one was not taken past the preliminary hearing;^160^ three were acquitted;^161^ seven were convicted with suspended sentences (plea bargains from murder to administration of a noxious substance or manslaughter);^162^ four were convicted on the lesser charges of manslaughter or administration of a noxious substance (one with two years probation,^163^ one with three years probation,^164^ one with two years in jail,^165^ and one with five years in jail);^166^ and two were convicted of murder, both with life sentences.^167^

As with assisted suicide, while the law is clear on voluntary euthanasia, it is also the subject of great controversy. As detailed in Chapter One, some believe that the law is appropriate and no law reform is required.^168^ Others believe that voluntary euthanasia should remain illegal but a third degree of murder (without the mandatory minimum life sentence) or a statutory defence to a charge of murder in cases of euthanasia should be introduced.^169^ Others still believe that voluntary euthanasia should be permitted in some circumstances and that the *Criminal Code* should be revised so that voluntary euthanasia could, under a regulated regime, take place.^170^ Again, there have been a number of attempts to change the *Criminal Code* through bills introduced in the federal parliament, but none have been successful.^171^ One of the three court challenges mentioned above in the discussion of assisted suicide also explicitly challenges the constitutional validity of the provisions of the *Criminal Code* that prohibit euthanasia.^172^ Again, time will tell whether the provisions can withstand judicial scrutiny.

## 7. CONCLUSIONS

It can be concluded that the legal status of some forms of conduct is clear, such as withholding and withdrawal of potentially life-sustaining treatment at the request of competent adults, assisted suicide, and voluntary euthanasia. Some are unclear, such as unilateral withholding and withdrawal, and terminal sedation. Some are very hotly contested, such as unilateral withholding and withdrawal, assisted suicide, and voluntary euthanasia. The Panel now turns to a consideration of the ethics of the controversial forms of conduct. In the next chapter, the Panel shifts the Report's attention from exploring what the legal status is to an ethical analysis of what kind of normative grounding public policy should have and where that grounding takes us with respect to what the law should be.

# Sub article: CHAPTER THREE: THE ETHICS OF END OF LIFE CARE

**Published:** 2011-11

## 1. INTRODUCTION

The previous chapters offer a clear picture of some of the challenges that lie ahead for Canada's health care system. Canada is undergoing a demographic shift that is changing the health profile of its population. Canadians are living longer and, as a greater proportion of the population reaches ages that only a small fraction of the population reached just a few generations ago, Canada's health system will need to address a broad range of issues that reflect the changing disease profile of its population. These issues do not just deal with the wishes of patients in the last few days of incurable disease, but also with patients suffering from chronic diseases and progressive cognitive impairment. For example, there are urgent questions to be addressed by policy makers regarding the lack of access to quality palliative care and the low rates of completion of valid and useful advance directives.

Chapter Two shows how unclear and unsatisfactory the existing legal framework for a significant range of end-of-life care decisions is. It is imperative that Canadians address the legal uncertainties and controversies that currently make decision-making so difficult for healthcare professionals, patients, and their families. The Canadian legal system's stance on a number of end-of-life issues is unclear because it leaves important questions unanswered. For example, who has the final say over the question of whether treatment should be withdrawn when the physicians want to stop treatment but the family disagrees? And how should the use of terminal sedation be viewed through existing legal categories? It is controversial, as there is a significant disconnect between the opinions of the majority of Canadians and the law as it now stands with respect to assisted suicide and euthanasia. There are also extremely strongly held (and, indeed, often polarized) positions with respect to what the law should be on a number of end-of-life decisions.

How should these issues be addressed? The Panel strongly asserts that they must be addressed in the context of responsible and rigorous ethical reflection.

Canada is a pluralist liberal democracy. Its vigorous defence of its citizens' basic freedoms, including freedoms of conscience, association and expression, give rise to a situation in which Canadians predictably reach a wide range of conclusions about ethical issues. As far as the *source* of ethics is concerned, some Canadians believe that ethics should be grounded in the will of God, while others believe that it should have more secular bases. Canadians citizens reflecting on important ethical issues in a context of freedom of thought and expression also reach quite diverse conclusions as to the *contents* of ethics, of the values that ought to have pride of place. Some believe that it should be about the protection of individual autonomy. Others think that it should ultimately aim to maximize happiness and well-being.

The Panel holds that in the context of such reasonable pluralism, the kind of ethical reflection that it engages in ought to be guided by values over which there is some significant degree of consensus within Canadian society.^173^ It ought to avoid taking sides on the kinds of deep disagreements that divide Canadians as far as the source and contents of ethics is concerned. In order to promote democratic discussion among Canadians, the Panel has sought to identify values over which Canadians broadly agree, and to trace what the implications of those values are for the issues related to end-of-life care.

How do we indentify the ethical consensuses that exist in a democratic society like Canada in the context of deep and durable disagreement as to the ultimate grounds and ends of ethics? The Panel holds that this should be done by looking to the ethical cornerstones of Canada's institutional order as a liberal democracy. A particularly rich fount of such values is our *Charter of Rights and Freedoms*, as well as the almost thirty years of legal and ethical reasoning that it has given rise to.

Of course, ethical reflection must also be informed by publically ascertainable facts (for instance, by epidemiological data such as is canvassed in Chapter One of this Report, and data from countries which have begun to deal with the issues identified above such as will be provided in Chapter Four). Canadians will only arrive at sensible policies and laws when ethicists and philosophers collaborate with empirical researchers, and make careful and responsible use of the data that they produce in order to enrich and to structure public debate. The facts matter; the uses made of them must not be clouded by prior ideological commitments.

To solve the problems identified in earlier chapters, our society's commitments to a range of central values must where possible be connected with the specific issues that arise in the context of end-of-life decision-making. This requires careful philosophical reasoning whereby the Panel tries to tease out, through argument, what the implications of our general ethical commitments are in specific issue areas. These philosophical arguments will, if successful, achieve a certain level of coherence both between general ethical commitments – as expressed, for example, in the language of the *Charter of Rights and Freedoms*– and laws and policies across different policy areas such as that of end-of-life care.

For some issues, these values, combined with the facts, leads us to justifiable conclusions about what the law should be. The Panel holds that there is both sufficient consensus with respect to core values in the Canadian policy context and a sufficient grasp of the relevant facts that justifiable conclusions can be drawn about what the legal status of assisted suicide and voluntary euthanasia should be. Detailed arguments in support of these conclusions are therefore presented in this chapter and recommendations grounded in these arguments are presented in Chapter Five.

For some issues, however, consensus on how to resolve competing ethical commitments or disagreements as to the relevant available facts is not yet available. Indeed, the values that, in the Panel's view, constitute the unquestioned core of Canadian public political culture, do not speak to all moral issues and in particular they do not speak clearly to the debate surrounding assisted death in non-voluntary contexts, that is in cases of individuals who are no longer, or who have never been, able competently to formulate wishes relating to end of life care. The Panel fully acknowledges that the consensus that we identify in Canada's political culture as a liberal democracy, and more specifically in its foundational texts and judicial decisions, occurs against the backdrop of a pluralism of reasonable comprehensive conceptions of the good, some religiously grounded, others secular in nature. The Panel also recognizes that the considerations contained in this report are not exhaustive of the philosophical landscape. Clearly some of these moral frameworks are comprehensive enough to apply to non-voluntary contexts. They are however insufficiently widely shared to constitute the basis for the kind of argument the Panel wishes to develop here, which attempts to ground substantive positions on the issue of assisted death in broad normative consensus.

For these reasons, the Panel does not for example address the conclusions that might be derived from the tradition of consequentialist philosophical theorizing that gives pride of place to the notion of *well-being*. Very generally speaking, such theories are united by a commitment to the idea that actions and policies are justified in as much as they promote aggregate well-being. Now, as has recently been argued very eloquently by the Canadian consequentialist philosopher Wayne Sumner, autonomy-based and well-being-based arguments tend to converge in the case of competent individuals, because they are best situated to know what their level of well-being is, and how it should be promoted.^174^ But one of the implications of well-being-based theories is that they allow the theorists who hold them to extend arguments about assisted death into non-voluntary contexts, that is, into contexts in which individuals are no longer, or have never been, capable of competently formulating their wills.

While the Panel fully recognizes the importance of the contribution made by well-being-based arguments to the philosophical literature on assisted death, it holds the view that such arguments are not sufficiently well grounded in Canadian public culture to allow grounding this analysis in such considerations. Conceptions of well-being are at present too diverse to ground a publically justifiable practice of assisted death in non-voluntary contexts on the basis of third-party assessments of the quality of life of a non-competent individual. Nor does the panel find guidance as to consensuses surrounding the concept of well-being that might exist in Canadian public political culture that might allow the Panel to make the kind of argument in non-voluntary contexts that it feels able to make in voluntary ones. To repeat, the intention of the Panel is to articulate the implications of public values that are deeply ingrained in the Canadian public political culture and institutions.

Rigorous ethical reflection is required in order to achieve coherence between various ethical commitments. Democratic deliberation is required where a society's ethical commitments are unclear.^175^ Decision-making about euthanasia will not always be able to ground itself in the value of individual autonomy, as there will be occasions when decisions will have to be made, for example, for patients in advanced stages of dementia and for patients who are in persistent vegetative states neither of whom have valid and relevant advance directives. It is clear that these decisions cannot always be made by referring to the patient's clearly expressed wishes (whether contemporaneous or prior). It is therefore not yet clear enough what values should guide decision-making about non-voluntary euthanasia in this category of cases which, as the epidemiological data presented in Chapter One have made plain, will be increasingly frequently encountered in the years to come.

Canadians have experimented successfully with democratic deliberative mechanisms on a range of issues such as electoral reform^176^ and many others.^177^ This experience can be usefully drawn upon to organize deliberation on these issues in years to come. The Panel's opinion is that an Expert Panel, such as this one, should not dictate the general compass Canadian society ought to use in addressing contested issues of value. The Panel therefore does not deal with non-voluntary euthanasia as it is an issue that cannot yet be resolved through reference to the kind of consensus that exists to address voluntary contexts and to facts that provide a sufficient base for justifiable conclusions.

This chapter examines the implications of what the Panel takes to be core normative commitments of Canadians as evinced by their constitutional framework with regards to the issue of assisted death. The structure of the chapter is as follows. First (section 2), the chapter identifies the core values that are sufficiently well grounded in Canadian political and legal culture to form the basis of an argument concerning assisted death in the case of competent agents. The Panel holds that respect for individual autonomy and self-determination represents such a core value. The Panel also recognizes the importance that the protection of vulnerable citizens and the promotion of ‘human dignity’ have in our constitutional tradition, and in particular in Supreme Court decisions surrounding end-of-life care. This chapter therefore attempts to interrogate critically and to articulate these core values so as to give rise to a set of recommendations that best coheres with these core moral commitments. Second (section 3), the Panel connects the conception of autonomy at work in the context of debates over assisted death with a conception that is already well entrenched in the theory and the practice of voluntary and informed consent. Third, (section 4), an important distinction between moral and legal rights is introduced, according to which the determination of whether or not there exists a moral right to assisted death establishes only a presumptive, or *prima facie* case for the desirability of establishing a legal right. Fourth (section 5), the chapter sets out the basic argument in favour of decriminalizing assisted death in the case of competent agents on the basis of the value ascribed by Canadian political and constitutional culture to the value of autonomy. In sections 6 and 7, the Panel considers the main arguments against decriminalization. Some of these arguments, considered, in section 6, are *a priori*, in that they contest that there is a *prima facie*, moral right to assisted death. In particular, some arguments grounded in the value of human dignity take this form. As human dignity has been invoked often by the Supreme Court of Canada, the Panel devotes particular attention to this concept.

Other arguments against the decriminalization of the right to assisted death are *a posteriori*, in that they concede the existence of a *prima facie* moral right but hold that other considerations weigh heavily against the recognition of a corresponding legal right. These arguments are considered in section 7.

One of these arguments is grounded in the concern that the decriminalization of assisted death in the case of competent agents might set us down a ‘slippery slope’ that will unavoidably place vulnerable persons at risk. Given the importance that the concern with the protection of the vulnerable rightly possesses in Canadian constitutional culture in general, and in the *Rodriguez* decision of the Supreme Court of Canada, that still forms the backdrop to much discussion of the right to assisted death in Canada, particular attention is devoted to such arguments.

## 2. CORE VALUES

What are the values over which there is broad societal consensus as evinced by Canada's foundational texts and institutions? The Panel holds that the value of individual autonomy or self-determination (we use these two terms interchangeably in the context of this Report) should be seen as paramount, though not as exclusive. Let us begin, then, by establishing the case for the paramount status of the value of individual autonomy.

There are several ways in which to establish the centrality of autonomy to a liberal democratic regime such as Canada. One way would be to advert to the history of political thought to see just how central the value of individual autonomy has been to the ethical and philosophical justification of liberal democracy. Whether one looks to John Stuart Mill's argument according to which one of the main functions of the State is to protect individuals' sovereignty with respect to their ‘self-regarding’ actions, to Immanuel Kant's definition of Enlightenment as ‘man's emergence from his self-incurred immaturity’, where immaturity is defined as ‘the inability to use one's own understanding without the guidance of another’, and where the responsibility of enlightened political institutions is to promote the enlightenment of citizens, or to Rousseau's ascription of a foundational status to autonomy for the ideal political order he envisaged, it has been clear to thinkers of liberal democracy that individual autonomy and liberal democracy are inextricably linked, with autonomy serving both as the normative ground and as the goal of liberal democratic political and legal institutions.^178^

Another way is by reflecting on the important status that individual rights protections have in modern liberal democracies. Though there are debates about whether such goods as health and welfare ought to be protected by rights, there is no debate about certain core civil and political rights that have to do with the protection of the individual's freedom of thought, conscience and association, as well as her bodily integrity, from unauthorized interference by the state, and with the capacity of individuals to exercise self-determination in the social and political arena. Clearly, the importance that liberal democratic thought and practice ascribe to these rights only makes sense if individual self-determination is given similar importance. Individual self-determination accounts for the central place that individual rights occupy in the institutional orders of liberal democracies.

The philosophical and institutional importance of individual autonomy for liberal democracies is clearly reflected in the decisions that have been taken by the *Supreme Court of Canada* since 1982, when a *Charter of Rights and Freedoms* was entrenched in Canada's Constitution, and in the language that the members of the Court have chosen to employ in order to justify their decisions. What is striking for present purposes is just how central they have taken individual self-determination to be, in particular in decisions that relate to the area of health care. Thus, for example, in *Ciarlariello v. Schachter*, the Court affirmed patients' rights to refuse treatment and to have treatment withdrawn even after it has begun, Justice Cory, writing for the Court wrote: ‘It should not be forgotten that every patient has a right to bodily integrity. This encompasses the right to determine what medical procedures will be accepted and the extent to which they will be accepted. Everyone has the right to decide what is to be done to one's own body. This includes the right to be free from medical treatment to which the individual does not consent. *This concept of individual autonomy is fundamental to the common law*’.^179^ The centrality is, importantly, also affirmed in *Rodriguez v. British Columbia (Attorney General)*, the case that reaffirmed the criminal status of physician-assisted death, where Justice Sopinka refers to the importance of ‘control over one's physical and psychological integrity’.^180^

Thus, whether we look at the matter historically, philosophically, or by adverting to the practice of the Canadian Supreme Court, it seems clear that the value of autonomy occupies a paramount place among the values of Canada's constitutional order. To the extent that the *Charter* enjoys broad support among Canadians as a framework within which to work out difficult questions of political morality in a fair and equitable manner, it follows that the cornerstone value of individual autonomy is at the centre of this consensus.

The value of autonomy of course does not stand alone among the values embodied in our constitutional and institutional order. Autonomy is properly conditioned and limited by considerations to do with (to use Justice Dickson's language in *R. v. Big M. Drug Mart Ltd.*), ‘public safety, order, health, or morals or the fundamental rights and freedoms of others’.^181^ Equality is clearly also a core value in our constitutional order, and the language of ‘human dignity’ also pervades the language of many Supreme Court decisions. In particular, it has been viewed by the Court as intrinsically connected to the value of equality. Indeed, the equality provisions of the *Charter* are seen as aiming above all for Canadians to enjoy equal dignity.^182^ (Dignity has also been connected by the Supreme Court to the value of individual self-determination, and to a host of other *Charter* values, a fact that makes it of dubious value in attempting to become clear on the specific ethical stakes that are present in the debates surrounding end-of-life care in general, and assisted dying in particular, a fact to which we shall return below). Thus, the Panel will be led to examining the ways in which these other constitutional values condition and limit autonomy-based arguments.

The task of this chapter is to spell out the implications of the broad normative commitments latent in the institutional culture of Canadian society, an institutional culture around which there is broad and stable societal consensus for the debate surrounding assisted death in voluntary contexts, that is in contexts in which individuals are capable of articulating their wishes competently. We consider, that is, whether a commitment to individual autonomy entails a *prima facie* right to choose assisted death on the part of ‘competent’ individuals (we will take up the concept of ‘competence’ below). If this implication were to hold, we then ask whether any of the other constitutional values that properly condition the value of individual autonomy should be taken as overriding the *prima facie* right.

Before we address these central questions, a pair of preliminary sets of remarks must be made. The first have to do with the nature of the concept of autonomy that we will be employing in the context of this Report. The second have to do with the various ways in which values can be used in arguments grounding and limiting legal rights.

## 3. AUTONOMY

There are many conceptions of autonomy.^183^ Some are purely procedural. These maintain that an individual is autonomous insofar as he is allowed to do what he wishes. This purely formal conception places no constraints on the procedures whereby a person has come to decide what they want. Whim, passing desire, and sober reflection are all protected by a conception of autonomy that merely seeks to protect the individual's ability to do his or her will against outside interference. At the other end of the spectrum lie substantive conceptions of autonomy that only protect individual choice when that choice has been the result of a very specific kind of reasoning. Immanuel Kant, the philosopher whose name is (perhaps misleadingly) most often associated with the concept of autonomy, thought autonomy requires that the rules which govern people's autonomous wills should be ‘universalizable’. Myriad conceptions lie in between these two extremes.

The ethical stakes involved in selecting a particular conception of autonomy are considerable. The more we tend toward the procedural end of the continuum of conceptions, the more we run the risk of claiming, even of a person who is in the grips of a passing whim or a compulsion, that they are autonomous. This is counter-intuitive, because implicit in the idea of ‘self-determination’ is the idea of a *self* who makes decisions about her actions on the basis of some standing set of convictions, plans of life, intentions, and the like. Arguably, we are ‘not ourselves’ when we merely succumb to a passing desire (unless we have deliberately made it into a policy to follow passing desires) or when we act compulsively.

But the more we tend toward the substantive end of the spectrum, the more we risk justifying a great deal of paternalism under the cover of a commitment to autonomy. For example, if we hold that only people who are operating in conditions of full information and ideal rationality are fully autonomous, we risk making autonomy into an unrealizable ideal. We also risk countenancing a great deal of difficulty to justify strong paternalistic intervention into the choices of individuals who do not meet the exacting standards of a highly demanding conception of autonomy.

Clearly, a morally attractive and operationalizable conception of autonomy would need to strike a reasonable middle ground between these two extremes. In deciding what conception of autonomy to make central to the argument of this Report, the Panel was once again guided by the concern that it should connect with values that are already deeply enshrined in central Canadian institutional commitments, and it should avoid imposing values that are not already settled parts of our ethical landscape. In this context, the Panel has chosen to be guided by the doctrine of *informed choice* that constitutes a central pillar of contemporary health ethics and of Canadian health law. Informed choice is grounded in autonomy; it seeks to apply the abstract value of autonomous decision-making to the context of health care. It requires that competent patients must not be subjected to treatment unless they have consented to it.^184^ That consent is subject to three conditions: first, it must be uncoerced; second, it must result from the decision-making capacity of a cognitively competent individual; and third, it must be informed. A conception of autonomy can thus be read in informed choice as the cornerstone of modern medical ethics and Canadian health law. An autonomous person would, according to this conception, be a substantively cognitively competent and uncoerced individual who arrives at his or her decisions after having been offered relevant information about the decision at hand.

Each of the conditions that constitute the modern doctrine of informed choice is subject to controversy. How much information is enough and what information is relevant? At what point does the cognitive decline of an individual become such as to detract from the appropriateness of thinking of him as autonomous? What role does the social construction of needs and wants play in a person's competence? Does the forceful attempt to persuade count as coercion? Does the absence of options constrain choice? All of these matters are subject to intense philosophical discussion that lie beyond the scope of this Report. But for the purposes at hand, the Panel considers it sufficient to link this conception of autonomy with the concept of informed choice that is deeply embedded in Canadian institutions, and to adopt the conception of informed choice that is presently at work in Canadian health law and in Canadian health care institutions. Any conception of autonomy that coheres with the doctrine of informed choice will incorporate a significant cognitive dimension, emphasizing the capacity to understand and appreciate the information at his or her disposal, the provision of information, and the voluntariness of the decision-making. The Panel's understanding of autonomy will not be purely procedural; that is, it will not take just any wish expressed by an individual as a manifestation of his or her autonomous decision-making capacity.

## 4. MORAL AND LEGAL RIGHTS

Should the criminal ban on medically assisted death be lifted? Should individuals possess a right to request of medical professionals that they assist them in dying? A first step in determining whether such a right should exist consists in determining whether or not there is a *moral* right to choose assisted death. The Panel takes a moral right to be a moral entitlement of a kind. For the purpose of determining whether such a moral entitlement exists we look at the moral values at stake, and determine whether, on balance, they justify a moral right.

Determining whether a legal right exists is a separate question. It does not follow from the fact that an individual has a moral right to X that she should also have the legal right thereto, nor does it follow that if she does *not* have the moral right to X, then she should not have the legal right to X. The existence of a moral right establishes a presumption in favour of the establishment of a legal right, but that presumption can be overturned by other considerations.

To see this, consider the argument put forward concerning such matters as recreational drug use and sex work. Some people believe that these activities should be made legal because, first, they hold a moral commitment to autonomy, and to the accompanying idea according to which individuals should be allowed to engage in whatever activities they see fit so long as no third parties are thereby harmed, and second, they hold that there are no countervailing reasons to ground a legal right upon the aforementioned moral right.

Some people may believe however that, first, the commercialization of sex and the use of recreational drugs are morally problematic, and thus should not ground a moral right, but that, second, there are reasons to recognize a duly constrained legal right to buy and sell sexual services or to engage in the purchase, sale and use of recreational drugs. They might affirm this second claim because they believe that certain core values, to do for example with the security of persons, are best realized through a regime of decriminalization and regulation rather than in a situation in which these practices generate harms because they are conducted in the absence of any regulation, and because the laws that criminalize the activities in question are unenforceable.

Opponents of certain legal rights may also argue to their conclusions by following one of two routes. They can claim that there is no moral right to X, and no reasons analogous to those that according to some exist in the case of sex work and recreational drug use, to grant a legal right. Or they can claim that there is a moral right to X, but that there exist countervailing considerations that justify that that right not be given legal expression.

This point is important in the context of the Canadian debate over medically assisted death because all of these positions have defenders in that debate, though the different logical structures of their different arguments are not always distinguished perspicuously. It is particularly important that we identify the nature of one of the central disagreements between the majority and the dissent in the *Rodriguez* case. To wit, they agreed that a prohibition on assisted suicide limited a person's right to autonomy but they disagreed about whether allowing the exercise of that legal right poses a threat to vulnerable persons, that is to persons who do not satisfy the criteria for autonomy described above, or whose request for physician-assisted death is not fully voluntary.

## 5. AUTONOMY AND ASSISTED DEATH

If autonomy is, as we claim that it is, a central constitutional value, then it quite clearly grounds the right to request assistance in dying according to one's considered and stable views about when one's own life is not worth living any longer.

The argument for this conclusion is fairly straightforward. If we believe that one of the roles of the state is to provide people with the institutional framework within which they can lead autonomous lives, that is, lives that reflect their values, convictions, and conceptions of what makes life worth living, it follows that the state should to the degree that it is able to protect citizens against obstacles to their being able to live their lives according to their own lights.

What's more, the state should be particularly vigilant in protecting citizens in this way with respect to the important choices in their lives. It is far more important that the state protect the citizen in her ability to choose a life partner than it is that it protect her in being able to choose certain breakfast cereals rather than others. Who to marry, what religion to profess, if any, whether to have children or not, these are choices that contribute powerfully to an individual's being able to view her life as one that corresponds to her ‘conception of the good life’.

Deciding how one will die clearly belongs to the choices that ought to be protected by the state, given our commitment to individual autonomy. The manner of our dying indeed reflects our sense of what is important just as much as do the other central decisions in our lives. Indeed, it seems contradictory to deny a person the right to live according to the values she thinks most important at the moment of her death after having put in place institutional mechanisms allowing her to follow these values in all other decisions of her life.

The commitment to autonomy, which as we have seen is a cornerstone of our constitutional order, thus quite naturally yields a *prima facie* right to choose the time and conditions of one's death, and thus, as a corollary, to request aid in dying from medical professionals.

## 6. LIMITS TO THE RIGHT TO MEDICALLY ASSISTED DEATH

We need now to address two distinct types of arguments. The first denies the conclusion that has just been argued for, claiming that the *prima facie* moral right that we have just argued for does not exist. The least ambitious such argument attempts to block the inference that many have made between the widely accepted claim that competent adults have the right to refuse medical treatment, or to have medical treatment interrupted once it has been started, even when it seems clear that abstaining from medical treatment will result in death, and the claim that competent individuals should have the right to choose assisted death. Indeed, some have argued that once one accepts the former practice, there is no moral ground for refusing the second. The argument against this view appeals to a pair of related distinctions, between intending a consequence and (merely) foreseeing it, and between doing and allowing. The argument is the least ambitious because while it establishes, if successful, that we cannot merely piggyback the moral acceptability of assisted death on that of the right to refuse treatment or to have it withdrawn, it does not tell us why assisted death is wrong.

Two further families of argument will then be considered that attempt to establish exactly that point. One such argument affirms the importance of autonomy, but argues that there are certain acts that autonomous choosers should never choose. The second holds that there are values that trump autonomy. We will in particular be focussing on the dignity-based argument against the *prima facie right* to choose assisted death, because dignity is a value that is quite regularly cited by the Supreme Court as central to the Canadian constitutional order.

The second set of arguments claims that though the *prima facie* moral right may very well exist, countervailing considerations exist that require that we abstain from giving legal expression to it. These arguments are paradigmatically expressed as ‘slippery slope’ arguments to the effect that if medically assisted death is legalized, it will be impossible for the state to protect its most vulnerable citizens, and thus to realize another important constitutional value, that has to do with the security of its residents.

### a. No Inference from the Right to Refuse Treatment to the Right to Assisted Death

The Panel will be reviewing in what follows a number of influential conceptual frameworks frequently invoked in discussions on end-of-life decision-making. These concepts do not necessarily have a bearing on the autonomy based analysis offered in this Report. Some have been included merely to ensure that the review of influential conceptual frameworks is reasonably comprehensive.

If healthcare professionals not only *can*, but *must,* adhere to the wishes of a patient who no longer wishes to be treated, does it not stand to reason that they should also be permitted to assist that patient in dying? Those who deny this logical implication must drive a hard conceptual and moral line between action and omission. They must claim that it is worse to bring about a person's death than it is to omit an act so as to prevent a person's death. They must also defend the view that there is a moral distinction between killing and simply letting die. Finally, they must hold that an individual is not morally responsible for the bad ends that they merely foresee will result from an action, but that they do not intend to occur. Do these distinctions withstand critical scrutiny?

The Acts and Omissions Doctrine (AOD) holds, essentially, that there is a moral difference between actively killing a patient and omitting to keep a patient alive when one could have acted, at a reasonable cost to oneself, to produce that same outcome. This view holds that omitting to keep, for instance, a terminally ill patient alive who does not wish to be kept alive is sometimes less – or not at all – morally objectionable than actively killing a terminally ill patient who requests active assistance in his or her dying. Robert Young offers a possible rationale for this point of view in more abstract terms:

Those who conceive of morality exclusively, or a least predominantly, within a traditional (deontological) framework claim, that *doing* something harmful is *intrinsically* morally wrong – that is, is morally wrong in itself, regardless of any good consequences it may produce. By contrast, when something similarly harmful is *allowed* to happen, a lesser intrinsic wrong is thought to be involved …. Those who think acts of killing are intrinsically worse [than letting die] can agree that a killing *typically* has extrinsic features that are lacking in an instance of letting die – malice, violation of the victim's rights, violence and so on – and that the presence of these features generally makes a killing far more reprehensible. What they insist, however, is that even in medical settings, where such extrinsic features are not normally present, it is intrinsically worse to do something harmful than to allow something harmful to occur.^185^

This latter view has been criticized as conceptually indefensible by numerous scholars. Jonathan Glover argues that the AOD relies on accepting the claim ‘that there is a moral difference between acts and omissions with the same total consequences.’^186^ He offers a number of possible rationales for such a view, including: the idea that acts are more likely to translate into certain outcomes than omissions; the view that actions more clearly result in identifiable victims than do omissions; that someone who acts is usually more causally connected to a particular outcome than someone who omits to act; and that someone who acts usually acts intentionally, while someone who omits to act does not.^187^ Are these kinds of reasons persuasive? Arguably not. If one were to compare actions and omissions using the same criteria, it becomes apparent that none of these arguments succeed. For instance, it is not necessarily the case that an omission to act is less likely than an action to guarantee a particular outcome. Similarly, it is not necessarily true that someone who omits to act is less causally connected to a particular outcome than someone who acted to achieve that same outcome. All of these suppositions depend upon the particularities of each case. There may very well be situations in which these differences are reversed, for example that an omission will more readily contribute to a particular outcome than an action. Imagine, on the one hand, that an individual omits to remove a baby from a shallow pond in which he or she may drown. In this case, that individual's omission has a clearly identifiable victim. Imagine, on the other hand, that an individual throws water balloons into a crowded room, knowing that they will damage the clothes of some people in the room, but not knowing which one. Indeed, for any reason provided by Glover to indicate why people might think that there are features about acting that impart greater responsibility than omitting to act, it is possible to construct cases with the opposite conclusion.

The distinction between action and omission falls apart. Both can be brought about by the same intention: that of bringing about a state of the world in which a patient will be dead rather than alive. Omissions can sometimes result not from intention, but from negligence. Though there are cases of people who have neglected to do something less responsible than had they intended and planned to do that thing (less responsible, but not completely exempt from responsibility), this is not the case when the omission is deliberate, as in the case where one passes by the baby drowning in the shallow pond, deliberates about whether or not to rescue it, and decides not to. Intending to omit to do something, with the intention of bringing about a consequence, seems not to have any of the features that would make an individual less likely to ascribe moral responsibility to certain omissions than to actions.

It is because intending to omit in order to bring about a result does not seem, morally, very different from intending to act in order to bring about that same result, that the AOD does not seem to have much relevance to end-of-life decision-making in clinical contexts. Indeed, though negligence does occur in clinical contexts, the types of cases considered in this Report are ones in which healthcare professionals omit to treat their patients in full knowledge that doing so might hasten their deaths (after having consulted with their patients about the course of action that they desire). The Panel analyzed whether, when both intention and outcome are held constant, there is something morally relevant that distinguishes action and inaction. The Panel asserts that there is not, and that attribution of responsibility must occur on a case-by-case basis, rather than on the basis of a conceptual distinction between *doing* and *allowing*, or between *killing* and *letting die*.

Another avenue that has been proposed to account for both the moral acceptability of current medical practice (for example, withholding treatment, use of potentially life-shortening analgesics and sedatives) and the moral condemnation of medically-assisted death, is the invocation of the *doctrine of double effect* (DDE) and the associated *intention-foresight distinction* (IFD).

Before analysing the tenability of the DDE, the Panel notes that in daily medical practice there is usually no need to invoke this doctrine to justify the use of palliative measures by a physician. In most cases, the dosages of drugs used are carefully modulated in such a way that no life-shortening effect needs to be assumed. Moreover, patients in severe pain can tolerate drug dosages that would quickly kill people who are not suffering pain. And even the reverse could happen as drug administration for pain control can itself prolong life rather than hasten death. In all such cases, physicians are not hastening the death of their patient so there is no need to invoke DDE to justify such treatment.

However, things are different when patients do die quickly after the administration of drugs and certainly when physicians administer sharply increasing dosages of pain medication with the clear knowledge that this administration is likely or even certain to hasten the patient's death. Here the DDE is sometimes invoked to justify the life-shortening effect of the intervention by the physician. The Panel analyses now whether the doctrine can be successfully used for that purpose.

The DDE and the IFD distinguish between the intended outcome of predictable (or foreseeable) actions and outcomes and those actions and outcomes that are not intended. Consider a healthcare professional who decides to prescribe a life-shortening amount of pain-killing medicine; a sanctity-of-life doctrine-supporting observer subscribing to the DDE would want to know whether the healthcare professional was intent on shortening the patient's life or whether he or she was intent on relieving the patient's suffering. The same act leading to the same outcome could easily translate into diametrically opposing ethical evaluations, depending on the doctor's intentions. It is somewhat doubtful that this argument can be successful. After all, a person is dead and that death was caused unequivocally by the doctor's decision to give a certain amount of pain-killing drugs. To claim that there is an important moral difference between doing ‘x’ in order to bring about ‘y’, and doing ‘x’ in order to do ‘z’– but in the full knowledge that it will bring about ‘y’– is to split hairs much more finely than is realistic in the context of real-life decision-making.

The DDE, historically, goes back to an argument advanced by St. Thomas Aquinas. He used the example of permissible homicide in self-defence. Aquinas imagined a situation where someone's intention is to fend off an attacker. However, while he or she succeeds in fending off the attacker, the attacker dies as a result of the defender's actions. The attacker's death was unintentional, and, according to Aquinas, is permissible ‘because self-preservation is a legitimate aim and a person who is unjustly attacked has a greater duty to preserve his own life than he has to preserve the life of the attacker.’^188^ The morally important claim here is that the person who is attacked permits the death of the attacker to occur, but does not intend for it to happen. The implicit understanding is that the death of the attacker is something that is undesirable and ought to be avoided. One assumption here is that the victim – the attacked person – is acting from good motives; or, as Suzanne Uniacke puts it, the ‘acts of double-effect are morally well-motivated.’^189^ The argument from the DDE relies on accepting a further concept, namely the IFD. This distinction upholds the view that there is a moral difference between intending a patient's death and foreseeing that it might happen, but not intending for it to happen. It is logically possible to maintain that, while one knows that to give a certain amount of pain-killers will lead to the death of the patient, one does not intend to kill the patient, and that one merely intends to alleviate the patient's suffering.

Views on the morality or immorality of what amounts practically to assisted dying are necessarily affected by arguments from the DDE and the IFD. Consequentialists will maintain that both the DDE and the IFD are irrelevant considerations in our evaluation of the morality of assisted dying. Either it is a morally good thing to offer assistance in dying to some people or it is not. Whether an individual foresees or intends for their action to assist a patient to die is morally uninteresting. What matters, on the background of the autonomy based ethical rationale laid out in this Report, is whether the result of the occurring assistance, namely the death of the patient, is what the patient desires.

Looking at matters more closely, the IFD – that is critical to the success of the DDE – poses problems for even its proponents. There are at least two reasons why this is the case. First, while the strict distinction between intended consequence and foreseen consequence may be plausible in theory, it is, in practice, extremely difficult to identify people's intentions with that level of precision. A person's exact motives are often somewhat opaque even to that individual; it would be wrong to ascribe complete reliability to the agent's capacity to know with complete certainty whether something that he or she knew would happen as a result of his or her action formed no part of the reason for which he or she did it. This problem is all the more significant when ascribing motives to others. A view of moral responsibility (grounded in a distinction that, in most ordinary cases, would render the attribution of responsibility impossible) should be rejected even by a deontologist. There is every reason to reject theories that are premised on exaggerated claims about the epistemic abilities of the people or institutions that will end up having to judge the rightness or wrongness of an action.

The second reason has to do with the undesirable consequences that an insistence on the IFD might have on a sense of moral responsibility. Do we want agents to feel as morally detached from the foreseeable consequences of their actions as the IFD would seem to represent? In general, people need to care about the foreseeable consequences of their actions. It is not desirable that they should invoke the fact that ‘they didn't do it on purpose’ as a way of getting out of moral liability. To the extent that people are expected to be morally responsible for the foreseeable consequences of their actions, it is strange to invoke a doctrine that does exactly the opposite in order to account both for the moral permissibility of widely accepted medical practices (use of sedatives and analgesics, withdrawal of treatment), and the putative unacceptability of actions that are done with the intention of killing (assisted suicide, voluntary euthanasia).

### b. A Priori Arguments: Suicide is not Choice-worthy

Is there a case, based solely on self-regarding obligations, for claiming that suicide constitutes harm to oneself sufficient to negate one's autonomy-based *prima facie* right to choose assisted suicide and voluntary euthanasia. Do people have an obligation to themselves not to end their lives? Such a case seems difficult to discern because, in the types of cases that interest this Report, the competent, informed, and uncoerced individual who constitutes our paradigm case does not view death as harm given her assessment that her life is no longer worth living for her.

The influential German enlightenment philosopher Immanuel Kant thought that there is an obligation to self not to commit suicide because suicide is a denial of one's rational nature. Kant's argument for this conclusion is not usually thought of as meriting too much attention, resting as it does on a notoriously tortured use of the idea that one should only act on maxims, or rules of conduct, that can be universalized. Part of Kant's point is, however, worth attending to: Kant believed that by committing suicide, an individual is somehow giving in to human inclinations (the inclination an individual might feel, for example, to turn their back on a difficult situation they could confront more forthrightly). To use modern parlance, Kant's view is that the person committing suicide takes the easy way out, where reason would dictate that he or she face their problems. It is in this respect that Kant thought that committing suicide turns against rational nature.

It is possible that some suicides conform to the Kantian picture. But again, in considering the situation of a person who has rationally reflected upon her situation and arrived at the conclusion that he or she wanted to end their life, the decision to commit suicide can be seen as a paradigmatic manifestation of rational agency rather than as its denial. If, as suggested above, the manner of one's dying is among the decisions that can reflect an individual's deepest values and commitments, then it would seem that Kant was wrong that in deciding to commit suicide, an individual was, in all cases, simply giving up and surrendering to inclination.

What of the person who does decide to commit suicide, or to request of others that they aid him in committing suicide, for reasons that might appear as frivolous? Do we not want to protect people against their self-destructively bad ideas? An obvious objection to the Panel's view according to which autonomous individuals should be permitted to determine for themselves when their life is no longer worth continuing, is that autonomous people sometimes make bad choices. It is conceivable, so the objection might continue, that a person might request assistance in dying for what could be ill-considered or frivolous reasons. Surely, the objection would conclude, such demands should not be met by an obliging health-care professional, nor should they be permitted by law. After all, permitting such bad choices would result in the destruction of someone's long-term ability to make autonomous choices in order to boast – one last time – her occurrent autonomy. If we value autonomy, so the argument might continue, we should aim to maximize it. Permitting someone to sacrifice their ability to make autonomous choices in the long-term in order to make one autonomous, but ill-advised, choice in the short-term does not seem to be sensible public policy.

This line of argument can be met in at least two ways. First, while this is the type of example philosophers are wont to invent to highlight purely logical problems with a philosophical position, it does not seem particularly plausible or indeed likely that competent people would request assistance in dying for no sound reason at all. Indeed, the empirical evidence available from jurisdictions in which a permissive regime exists (as described in Chapter Four) provides no basis for believing that individuals seek assistance for ill-considered or frivolous reasons. Remember that our account of autonomy is not purely procedural. That is, it does not require that we respect just any choice that individuals make while in the throes of whim or compulsion. The justification of assisted death in this Report concerns individuals who have been provided sufficient information to understand the nature and consequences of their decisions, and who possess the various cognitive abilities that constitute competence.

What of the theoretically possible though unlikely case of an uncoerced competent person who with sufficient information nonetheless decides to die for frivolous reasons. Our commitment to autonomy requires that we bite this theoretical bullet. Respect for autonomy requires respect for what others might consider to be bad decisions as long as those decisions are free and informed and made by competent individuals. This is reflected in bioethical and legal analyses of potentially fatal decisions ranging from refusals of potentially life-sustaining treatment, to participation in risky behaviour (including cosmetic surgery and climbing Mount Everest), to suicide.

We must now consider a third kind of *a priori* argument aiming at denying that there exists a presumptive right to choose assisted suicide. This argument is to the effect that such a presumptive right would be contrary to human dignity. Given the historical prominence of this argument in the debate surrounding euthanasia, we have chosen to consider it at particularly great length.

### c. A Priori Arguments: Suicide Offends against Human Dignity

This section evaluates the suitability of arguments grounded in human dignity as a means of meaningfully addressing normative issues that affect end-of-life decision-making. The Panel's conclusion is that while the language of *human dignity* is seemingly universal, there is currently no consensus on the moral basis or on the precise meaning of human dignity. It is unclear whether it should best be understood as a basic, or primitive, term of moral language or whether it might reasonably be derived from a moral theory of mainstream appeal. Unsurprisingly, given its vagueness human dignity is currently being deployed in ethical, political, and even legal contexts in support of diametrically opposing points of view. In this section, the Panel concludes that the concept of human dignity is an unsuitable tool for settling normative questions pertaining to end-of-life decision-making.

The Panel approaches the task at hand by sketching the notion's historical use as well as its significance in a recent landmark Canadian Supreme Court ruling. The last part of this section provides an ethical analysis of different influential philosophical approaches to human dignity. In all three instances examined here, dignity is used to encompass a variety of quite distinct normative considerations. The section ends with a call for arguments to be made on the basis of these normative considerations, rather than in terms of dignity, which too often functions as a rhetorical tool to gain assent to normative positions that would otherwise seem controversial and unacceptable to some or many Canadians. The length of this section is a direct reflection of the significant role that the concept of dignity plays in debates about voluntary euthanasia and assisted suicide and the need to get past it if we are to make any progress in breaking the apparent deadlock that grips discussion of public policy on these issues.

The trope of human dignity pervades debates about end-of-life decision-making and is present in many other spheres of social life. David A. Hyman notes:

… in every generation, philosophers, ethicists, religious figures, politicians, and professional worrywarts have cited human dignity as a reason to restrict innovation or prohibit it outright. Consider a few examples. Galileo was forced to recant his heliocentric views because the Roman Catholic Church had already embraced the Ptolemaic system as more consistent with Biblical revelation and with man's dignity as God's creation. Indoor plumbing, the printing press, skyscrapers, the suburbs, automobiles, television, the Sony Walkman™, and the franchise for women were all met with the objection that they were inconsistent with human dignity. The Industrial Revolution, which laid the foundation for the modern world, was criticized because machines were expected to destroy human dignity.^190^

In the context of end-of-life decision-making, arguments about the question of whether particular tools and mechanisms designed to bring about the death of a patient are dignity-violating continue unabated.^191^,^192^ Sensen adds that ‘human dignity is currently presented as *the* justification for human rights.’^193^ In the medical context, human dignity holds a prominent place. The World Medical Association's *Declaration of Geneva* demands that doctors treat their patients with ‘compassion and respect for human dignity’.^194^ This requirement was eventually extended to cover biomedical researchers' treatment of participants in clinical trials.^195^ Similar pronouncements have been made by the World Health Organization.^196^ In Canada, the Tri-Council's Policy Statement notes that respect for human dignity ‘has been an underlying value of the Tri-Council Policy Statement: Ethics Conduct for Research Involving Humans (TCPS or the Policy) since its inception.’^197^

Van der Graaf and colleagues, among others, have attempted to categorize different historical understandings of dignity.^198^,^199^ Their contributions show that the language of ‘human dignity’ is used in the healthcare context from Roman antiquity to today. Throughout history, human dignity has been used in a variety of different, and often overlapping, contexts. From antiquity to the Middle Ages, through the Renaissance and, arguably, to Enlightenment thinkers such as Kant, *human dignity* was used to emphasize human beings' special place in the universe. The criteria drawn on to make this case were typically the capacity to reason, the ability to make use of freedoms and the ability to give intellectual direction and order to one's life. This, so it was argued, distinguishes humans from non-human animals. This, most obviously, leaves open the question of whether humans without these dispositions do not possess human dignity. Indeed, philosophers of very different backgrounds – utilitarian and Kantian respectively – have concluded that this rationale for a different moral status of non-human animals is anything but convincing.^200^ The traditional paradigm of human dignity seems to have little in common with today's, however vague, understanding. For instance, dignity in the traditional understanding does *not* serve as a moral basis of rights' claims. Dignity, according to the traditional understanding, is more concerned with ensuring that humans live up to a standard of ethical living that is required by an inherent dignity within.

Today dignity is frequently understood as some kind of intrinsic, morally relevant value that places a moral obligation on the individual (and on others) to respect someone by virtue of their dignity.^201^ Sensen points out that human dignity, in this contemporary understanding, forms the moral ground of human rights in UN documents. For instance, in the 1948 *Universal Declaration of Human Rights*, human rights are grounded in ‘the recognition of the inherent dignity […] of all members of the human family.’^202^ Sensen sees a problem in the UN approach on this issue:

[i]n documents like these [UN declarations, covenants] key terms are deliberately kept vague, since one can only secure an agreement among so many parties at the price of a certain ambiguity. If one were to specify the meaning and grounding force of human dignity, it might be at odds with some parties' deeply entrenched opinions and beliefs. In this case the whole project might fail. Accordingly, there is no explicit attempt to clarify or justify human dignity in these documents.^203^

A good example of this, in the field of bioethics, is the UNESCO Declaration on Bioethics and Human Rights. It deploys human dignity to ground the substantive policy guidance contained in this document.^204^ David Benatar agrees with Sensen's take on the popularity of vague language in this dignity-centred declaration. He argues, ‘[t]he other way to gloss over disagreement is to choose formulations that are sufficiently vague that each person can interpret them consistently with his or her own views.’^205^ The Panel returns to this problem throughout this section as it has significant ramifications for the subject matter of this Report.

It is evident, from this brief survey, that there is no consensus among experts on what the moral basis of human dignity is, if any, and what its specific meaning in the health care context should be taken to be. It is thus not surprising to find the term invoked on both sides of the debate over assisted dying. The Panel's survey of the meanings of this term – both its current and historical usages – suggests there is no way to point to a canonical, or ideal, conceptualization; it cannot be proven that either side of the assisted dying debate is properly applying the concept. Indeed, it is uncertain that there is unequivocally a concept to begin with.

On one side of the ledger, certain religious organizations and institutions have used the language of *human dignity* as a means to reject any suggestion that assisted dying should be decriminalized. For instance, the Roman Catholic Church considers assisted suicide and euthanasia to be a ‘violation of the divine law, an offence against the dignity of the human person, a crime against life, and an attack on humanity.’^206^ Recently Margaret Somerville insisted that, ‘the concept of dignity must be used to maintain respect for the life of each person, and for human life and for the essence of our humanness, in general. The current danger is that in the euthanasia debate it could be used to realize precisely the opposite outcomes.’^207^ Accordingly, Somerville believes the decriminalization of assisted suicide and voluntary euthanasia would be such undesirable outcomes.

On the other side of the argument, Raphael Cohen-Almagor, to name but one example, has published *The Right to Die with Dignity*, a monograph proposing the decriminalization of assisted suicide on the basis of considerations of dignity.^208^ What's more, organizations campaigning in favour of the decriminalization of assisted dying (in some shape or form) do not hesitate to campaign in the name of ‘human dignity’. Indeed, some, like the well-known Swiss organization *Dignitas* offer assisted-dying services to terminally ill patients.^209^ Joel Feinberg offers some justification for this view when he writes, ‘human dignity is not possible without the acknowledgment of personal sovereignty.’^210^ This strategic positioning and the attempt at taking ideological ownership of the term *human dignity* is unsurprising, given its near-universal appeal. However, as will become evident from currently available analyses of the term, neither proponents nor opponents of assisted dying will be able to resort to language of ‘human dignity’ as some kind of trump in support of their views.

To determine whether things are any clearer in the usage that is made of the term in Canadian jurisprudence the Panel examined the Supreme Court of Canada decision in the case of Sue Rodriguez, in which the term was used frequently in both the majority and in the minority opinions.

In September 1993, the Supreme Court of Canada decided on the petition by Sue Rodriguez – a terminally ill patient in advanced stages of amyotrophic lateral sclerosis (ALS) – to declare invalid section 241 (b) of the *Criminal Code* which criminalizes assisting people to commit suicide.^211^ Ms. Rodriguez argued that section 241(b) of the *Criminal Code* violated her rights under the *Canadian Charter of Rights and Freedoms*. Specifically, Ms Rodriguez argued that denying her access to assisted suicide denied her constitutional rights as granted under sub-sections 7, 12 and 15 (that is the right not to be deprived of the rights to life, liberty and security except in accordance with the principles of fundamental justice, the right not to be subjected to cruel and unusual punishment, and the right to equal treatment under the law).^212^ The Supreme Court of Canada, in a five to four majority decision, denied the appeal.^213^

Surprisingly judges in both the majority and minority made reference to human dignity to justify their respective decisions. The majority decision reads, in part:

[a]s members of a society based upon respect for the intrinsic value of human life and *on the inherent dignity of every human being*, can we incorporate within the Constitution which embodies our most fundamental values a right to terminate one's own life in any circumstances? [emphasis added]^214^

The majority of Supreme Court of Canada judges' answered this question with an unequivocal no.

The judges who wrote to grant Sue Rodriguez's appeal also framed their arguments partly in terms of dignity. Justice Cory expressed his stance this way:

… the Canadian Charter of Rights and Freedoms has granted the constitutional right to Canadians to life, liberty and the security of the person. It [s.7] is a provision which emphasizes *the innate dignity of human existence*[emphasis added] …. [It] follows that the right to die with dignity should be as well protected as any other aspect of the right to life.^215^

Justice Beverley McLachlin (as she then was) wrote:

Security of the person has an element of personal autonomy, *protecting the dignity* and privacy of individuals with respect to decisions concerning their own body. It is part of the persona and dignity of the human being that he or she have the autonomy to decide what is best for his or her body [emphasis added].^216^

The judges of the Supreme Court of Canada availed themselves of the language of human dignity as a means to support diametrically opposed results, namely the continuing criminalization of assisted suicide on the one hand, and the decriminalization of assisted suicide on the other. The majority of judges on the Supreme Court of Canada expressed a particular understanding of the dignity of human beings. Pullman argues that they were concerned that ‘a liberal assessment of individual cases eventually will erode society's perception of the intrinsic worth of human life in general.’^217^ One of the dissenting opinions of the court saw individual dignity as disrespected when the individual's right to self-determination is ignored. Justice McLachlin (as she then was) asked at the time: ‘What value is there, in life without the choice to do what one wants with one's life?’^218^

Thus, just as no conceptual clarity on the concept of dignity is gained by looking at historical sources, it is also impossible to move forward by examining the use to which the concept of dignity has been put in the main Canadian Supreme Court decision addressing the issue of assisted suicide.

Might an examination of contemporary moral theory address this conceptual quagmire? It is possible, after all, that one of the opposing sides claiming human dignity as its own is just wrong in its use of the term, and that moral philosophy will provide contours of the concept that will enable a decision on which of the two camps is closer to what human dignity really denotes.

It is worth approaching the analysis of the concept of human dignity in the context of assisted dying by asking, first, whether it is either a primitive (or self-evident) term of moral language or whether it is derived from a substantive moral theory with significant general appeal. Once the moral foundation of human dignity is understood, the Panel can address the question of the validity of its guidance.

Robert Goodin argues, in an influential account of human dignity, that it is impossible to ground human dignity in a moral theory. However, he insists that we should accept ‘human dignity’ as a logical primitive, ‘a fundamental axiom in our individualistic ethical system’.^219^ This, of course, is begging the question. Goodin, after conceding that dignity cannot sensibly be derived from an ethical framework, suggests that we should accept it as a primitive term of moral language. The problem is, unfortunately, that the meaning of the term is not self-evident to begin with and so, in that sense, the question is left unanswered.

Broadly speaking, in addition to the contemporary, intuitionist account of human dignity, there are arguments in moral philosophy about whether human dignity is a species-related concept that is applicable to all human beings *as* human beings regardless of a given member of our species' dispositional capabilities,^220^ or whether human dignity applies only to a person capable of making rational choices.^221^

Historically, species-related dignity claims have been derived from a religious idea, as expressed in the Book of Genesis, suggesting that God made the human species the apex of earthly creation. This metaphysical claim rests on ‘the Judeo-Christian appeal to the *imago dei*[image of God] as the basis of human dignity.’^222^ It has been suggested that this traditional understanding of human dignity was first explicated by Pope Leo I. He reportedly said, “[r]ealize, o Christian, your dignity. Once made a ‘partaker in the divine nature’, do not return to your former baseness by a life unworthy [of that dignity] …. Recall that you have been made ‘according to the image of God’.”^223^ On a policy level, such claims translate into stances such as this, taken by the Canadian Salvation Army:

Human life is a sacred gift from God. The Salvation Army believes, as a consequence, that euthanasia and assisted suicide are morally wrong …. Euthanasia undermines, rather than enhances, human dignity …. Assisted suicide undermines, rather than enhances, human dignity.^224^

Ethics has at least two primary functions: to guide our actions, and to provide justification for the guidance given. This approach to human dignity seems to meet the guidance criterion. Most of those appealing to this understanding of dignity reject any kind of assisted suicide or euthanasia. The reason frequently provided is that the God in question is the sole arbiter of life and death and that mere mortals are not permitted to interfere with God's master plan. God is understood to have given humans life, and humanity is not entitled to take this gift away on its own accord.

The underlying premise – namely that all humans are possessed of dignity in virtue of a special relationship to a God – is, however, incapable of being used as a basis of public policy proven in the context of a democratic, multicultural and multi-faith society that must cleave to the strictures of public reason in ethical deliberation. In the absence of a societal consensus in favour of, or incontrovertible proof of the existence of the God in question, and, therefore, the absence of overwhelming societal support for the metaphysical claims underlying this grounding of dignity, this account of human dignity cannot be relied upon to justify normative guidance on assisted dying on a societal level. Religious people might choose to avail themselves of the guidance provided by their respective religions, but it is unreasonable to enforce normative views, derived from claims about a God, uniformly on a societal level given the multicultural and multi-faith nature of Canada in the 21st century.

The same holds true for secular accounts arguing against the decriminalization of assisted dying on species-membership accounts. Leon Kass' account is probably the best-known of these approaches. He famously argued that ‘the deepest ethical principle restraining the physician's power is not the autonomy or freedom of the patient; neither is it his own compassion or good intention. Rather, it is the dignity and mysterious power of life itself.’^225^ This approach to human dignity is again metaphysical in nature. It asserts that species-wide dignity is a kind of ethical primitive that imposes limits on all other ethical theories, as Richard E. Ashcroft has suggested. He notes that

this approach is set out in direct challenge to the dominant ‘personhood’ account, which starts from what is morally valuable in paradigm cases of humans and then generalizes to other entities with similar features, while restricting concern (to varying degrees) to entities lacking some or all of the features of personhood. The dignity approach rejects this strategy, in part because it undermines the moral status of ‘marginal’ humans (such as embryos and people in a persistent vegetative state), and in part because it holds that dignity (or humanity) is ‘primitive’ in that it cannot be analyzed further into contributory components. Personhood is not ‘primitive’ in this sense, and the arguments about who does or does not possess it are widely criticized by non-bioethicists as attacks on vulnerable people. On the other hand, Kass' metaphysical dignity is equally obscure, precisely because it is held to be a primitive term.^226^

The challenge for non-theological, species-wide conceptions of dignity is that once the argument about having been made by and in the image of God is taken away, it seems impossible to point to some trait possessed by all humans, and only by humans, that grounds the attribution of dignity to them. Species-wide conceptions of dignity are thus, it would seem, questionable.

Kurt Bayertz rightly points out that such appeals to human dignity and to the sanctity of human life have, for all practical intents and purposes, become efficient stop signs both in philosophical discourse and public policy debates. These appeals are aimed at pre-empting any further debate on the matter.^227^ Attempts at linking appeals to dignity and the sanctity of human life have been widely criticized by philosophers from various, often even competing, philosophical traditions during the last few decades. In a similar vein, court judgments have repeatedly and explicitly rejected a fundamental assumption inherent in claims about human dignity and the sanctity of human life, namely that continued existence is always of benefit to the person in question.^228^

Immanuel Kant developed not a species-specific, but a person-specific account of human dignity. Respect for dignity, according to his understanding, is owed to us not as a result of our membership of our species but rather, to our the human capacity for living a life based on rational choices as a dignified existence requiring moral respect. It is that which distinguishes us from non-human animals because it permits us to overcome natural necessity. In Kant's ethics, all persons with the disposition to reason are ends in themselves; they have intrinsic, infinite value. One implication of this view is that persons must never be used as mere means by others, and that they must never treat themselves as mere means either. The latter point, particularly, could have serious consequences for the debate on assisted suicide and voluntary euthanasia. The point of this view is that humans ought to respect persons' dignity because of the value ascribed to their capacity to make rational, moral choices, and to respect them for who they are as self-conscious beings.

But if this is what grounds dignity, then it is unclear why it should (as Kant thought it did) be viewed as part of an argument against the right to determine the circumstances of one's dying. Indeed, if people's ability to reason is respected, then their ability to reason about the ends of life, and about the end of life, must be respected as well. Why is the decision to request aid in dying in the face of a subjective conclusion that one's life is no longer worth living (and in full cognizance of the facts about one's circumstances) a denial, rather than an affirmation, of dignity?

Kant clearly thought that an autonomous person – a person who made use of his or her rational capacities in order to determine his or her course of action – would never choose to commit suicide. But this, as has been noted, was based on his somewhat tortured reasoning according to which, necessarily, a person who chooses to die in order to put an end to suffering is treating him or herself as a means rather than as an end:

If he destroys himself in order to escape from a difficult situation, then he is making use of his person merely as a means so as to maintain a tolerable condition until the end of his life. However, a human is not a thing and hence is not something to be used merely as a means; one must in all one's actions always be regarded as an end in itself. Therefore, I cannot dispose of a human being in my own person by mutilating, damaging, or killing him.^229^

J. David Velleman, a current-day Kantian agrees. According to his analysis, we ought to value the person in a particular special way that does not, by definition, permit us to balance dignity against other values (like respect for a patient's autonomous choices – a choice Frances M. Kamm considers to be protected). Velleman summarizes his argument thus:

The question is whether the self-interested choice of suicide can really be a ‘reasoned choice’– this question being foundational, in Kantian ethics, to the question whether such a choice is morally permitted. The answer is that the self-interested choice of suicide cannot be an exercise of rationality, because it entails treating oneself as an instrument of one's interests, which is incoherent. That's why this choice is not morally protected. One's value as a rational being cannot require that others defer to one's irrational disregard for that same value.^230^

Velleman similarly maintains that pain and suffering are not sufficient dignity-related reasons for legalizing assisted dying. His argument follows these lines of thinking: dignity of persons is abused if the person is eliminated in order to end her pain and suffering, that is if it is used purely as a means to achieve another end. The intention to act in such a manner is not only disrespectful to an individual's own dignity as a person, but also to the dignity of similar persons. In that sense, the intention is not merely self-regarding but also other-regarding.

That this is not an obvious implication of Kant's commitment to autonomy is evidenced by the fact that some influential contemporary Kantian moral philosophers are critical of this conclusion.^231^ Kamm argues that when ‘life involves such unbearable pain that one's whole life is focused on that pain,’ dignity, as a person, is, arguably, lost. Her well-known analysis begins with the observation that persons have a right to life, and, as a corollary, a right not to be killed. However, persons are entitled to waive their right to life. Waiving this right, according to Kamm ‘releases others from a duty not to kill him’.^232^ She continues,

[s]uppose life involves such unbearable pain that one's whole life is focused on that pain. In such circumstances, one could, I believe, decline the honour of being a person. […] We might acknowledge the great (and normally overriding) value of being a person [… and yet] allow that some bad conditions may overshadow its very great value.’^233^

Kamm subscribes to the view that respect for self-regarding decisions as moral agents is what is required by human dignity, whereas Velleman holds that dignity is incompatible with making certain decisions, the decision to commit suicide is such a decision.^234^

One plausible diagnosis of this apparent failure among Kantians to fix on a roughly similar conception of dignity is that they have different things in mind. They are invoking their favoured moral considerations and cloaking them in the (rhetorically compelling) language of dignity. Kamm tightly ties dignity to the ability to exercise personal autonomy. This tendency among Kantians has prompted medical ethicist Ruth Macklin to suggest that dignity collapses altogether into respect for personal autonomy, and, for that same reason, notions of dignity should be discarded and replaced with respect for personal autonomy.^235^

It is clear that Kantians like Velleman have not moved their analysis significantly beyond Kass in simply pointing to a dignity that inheres within us and that limits the decisions we can take. The problem is that, in the absence of a theological grounding for this claim, it is metaphysical in the pejorative sense of the term and is rooted in neither argument nor observation.

It is evident that the influential Kantian approach to ethics does not provide an unequivocal ethical guidance and justification on the issue of assisted dying. Society holds diverse and often contradictory views on what constitutes a life worth living, and, more importantly, on what constitutes dignified or undignified death. Not unexpectedly, among Kantians there is no consensus on whether or not reference to human dignity is a suitable tool to settle the question of whether or not assisted dying could be ethical.

To summarize thus far, the Panel has examined historical sources, Canadian Supreme Court decisions, and recent moral philosophy. The Panel has observed the same pattern running through all three, namely a tendency to make dignity mean one thing and its opposite, and to cloak potentially controversial moral consideration – individual autonomy on the one hand, and some quality inherent within agents, in virtue of their being human on the other – in the pleasing language of dignity. In light of this conclusion, the Panel asserts it is best that debate about moral issues, such as assisted death, absent discussions of *human dignity*; rather, the values that lie behind this concept, on both sides of the debate, be explicitly considered.

More pointedly, there is a challenge for those theorists and activists who would limit the individual's right to make informed and rational decisions on the conditions of his or her death in the name of some normative consideration that inheres in individuals as either rational beings or as members of the human species. This challenge is to specify what that normative consideration is without invoking the language of dignity and without invoking considerations that are either implicitly or explicitly theological, and, thus, are unacceptable given the canons of public reason that are essential for public debate in multicultural and multi-faith societies such as Canada. While the Panel does not foreclose the possibility that such an argumentative hurdle can be cleared, the Panel does not at present see that any contribution to the debate has actually done so.

## 7. ARGUMENTS AGAINST THE *LEGAL* RIGHT TO ASSISTED DEATH

The Panel takes itself to have established in the previous section that extant *a priori* arguments against the *prima facie* moral right to assisted death are unpersuasive. Note that this conclusion is weaker than would be the claim that it is in principle impossible to articulate a publically acceptable argument against even a *prima facie* right. Given the importance of autonomy in our constitutional order, the burden of proof is quite substantial.

We must now consider arguments to the effect that though there may very well exist a *prima facie* right to assisted death, this right is defeated by countervailing considerations, such as the rights of third parties.

How might such an argument be mounted? Clearly, one's suicide affects others in ways that are often quite tragic. A person who chooses to die leaves behind loved ones whose grief might immense. Might this kind of interest which third parties have in an individual not being permitted to exercise a *prima facie* right to assisted death be the kind of third-party interest that might block the recognition of a legal right?

We can dispense of this kind of argument quite readily. Though there is no denying the suffering that suicide can cause among a person's family and friends, we have chosen not to let such considerations constitute a legal break upon individuals being permitted to act on their autonomous choices, especially with respect to life's most important decisions. In addition, we allow individuals to leave their families even in cases where their departure causes great grief or hardship. There is no reason to treat the case of assisted suicide any differently.

More to the point is the impact that allowing assisted suicide might have upon vulnerable others. A concern voiced at least as often as the concern, considered above, that suicide offends against human dignity, is that by allowing assisted suicide in the case of competent individuals, we will set in motion a ‘slippery slope’ that will lead us inexorably to employing euthanasia unacceptably in non-voluntary contexts. This line of argument warrants our attention because it invokes a value that clearly has the same kind of foundational status that autonomy has in our constitutional moral order. That value is that of the safety and security of the Canadian population, and in particular of its most vulnerable members.

Another line of argument that might block the move from the moral right to suicide to the legal right to *assisted* suicide has to do with the rights and interests of medical professionals. What we are trying to determine is not whether there exists a right to suicide but rather whether there is a legal right to be aided by medical professionals in ending one's life. The first right may very well exist, but it only gives rise to the second in the case there is no legitimate claim that we might make to the effect that medical personnel ought not to aid their patients to avail themselves of their right to choose suicide.

The following section will be devoted to the consideration of the claim that, whatever the status of the right to suicide, there is no right to choose *assisted* suicide because medical professionals are under an obligation not to assist their patients in dying. We will then consider arguments to the effect that the recognition of the legal right to choose assisted suicide would set up a *slippery slope* that would end up sacrificing the important value of safety and security of our most vulnerable fellow citizens.

### a. Medical Professionals

If the arguments canvassed so far are at all plausible, then it follows that respect for individual autonomy grounds a moral right not to be interfered with in requesting voluntary euthanasia or assisted suicide, and that this moral right is not defeated by obligations that the individual making the request might have toward himself, or by considerations of human dignity.

The next task is to determine what the status of healthcare professionals is with respect to this right. Ought they to provide assistance with suicide or voluntary euthanasia? Are they, on the contrary, duty-bound not to accede to requests for assistance with suicide or voluntary euthanasia in light of independent moral considerations? The Panel defends and takes an intermediate position in this section: healthcare professionals may accede to the request made by an autonomous individual for assistance with suicide or voluntary euthanasia, though they are not professionally duty-bound to do so. For reasons which will be explained below, their right not to accede to the request is limited in the following way: if they choose not to assist a competent and fully informed patient who has made an uncoerced request for assisted suicide or voluntary euthanasia, they are duty-bound to refer them in a timely fashion to a healthcare professional who will.

It has been argued that, although patients may formulate a morally legitimate request for assisted suicide or voluntary euthanasia, healthcare professionals have the obligation not to accede to their wish. It is claimed, for example, that assisting another person in dying is incompatible with the professional ethical obligations of a healthcare professional, who traditionally is understood to be morally obligated to heal rather than to kill. It is also claimed that an erosion of trust between the general population and the medical establishment would ensue if health care professionals came to assist their patients in dying as a matter of routine.^236^ Rather than trying to determine whether there is some deep incompatibility within the ethics guiding healthcare professionals' conduct, it is helpful to proceed by examining whether assisted suicide and voluntary euthanasia are compatible with acts that healthcare professionals already perform as a matter of course, and which are widely accepted both in Canadian law and in ethics.

The practice of medicine has evolved in such a manner as to legitimate healthcare professionals providing their patients with assistance in dying. Patients and healthcare professionals already discuss whether a patient's best interests are promoted by continued living when questions of termination of treatment arise. The patient's rights most decidedly include the right to refuse treatment and the right to interrupt treatment even after it has been initiated. Decisions to terminate treatment are routinely taken by patients following discussions with their doctors and nurses, who attempt to paint as clear a picture as they can of their patient's prognosis in order to allow for an informed decision. When the patient decides that the time has come to suspend treatment, healthcare professionals are duty-bound to accede to their wishes, even when it is clear that termination of treatment will hasten death. It is uncontroversial then that it is accepted in the healthcare professions today that the healthcare professional's role is not limited to providing therapy.

The Panel concludes that there is both a moral right on the part of informed and competent patients who do not consider their lives worth living any longer, to non-interference in assisted suicide and voluntary euthanasia, and a moral permission on the part of healthcare professionals to provide assistance with suicide or voluntary euthanasia. It is worth noting in this context that the Panel has deliberately chosen to analyse the involvement of *healthcare professionals* as opposed to only the involvement of medical doctors.

A question may arise as to whether the Panel considers there to appropriately be a *permission* rather than an *obligation* on the part of healthcare professionals to provide assistance with suicide or voluntary euthanasia to those who have decided to die. Society has a significant interest that if the practices of assisted suicide and voluntary euthanasia are carried out at all, they must be carried out securely, and with appropriate mechanisms of oversight, protection, and control. Where does this leave healthcare professionals? An obligation on healthcare professionals would obviously bring their professional as well as their personal autonomy into play. A question may also arise as to whether the Panel considers there to appropriately be a *restriction* on healthcare professionals in the provision of assistance with suicide or voluntary euthanasia. These two questions are interrelated, as they both implicate the interests of others beyond the individual seeking assisted suicide or voluntary euthanasia and the answer to one affects the answer to the other. If healthcare professionals are obligated to provide assistance, then their autonomy is evidently limited. It is worth noting, however, that obligations imposed on professionals are not as such morally questionable, indeed they are not unusual. Limitations of professional autonomy are the traditional societal response aimed at regulating the professions. The question is whether any given obligation placed on professionals is justifiable.

It arguably would constitute a limitation of healthcare professionals' liberty or freedom of conscience and religion if they were required to accede to the voluntary request for assistance with suicide or the request for voluntary euthanasia, even if the request was formulated by a competent and informed patient.^237^ Today's procedural solution to this problem is, in Canada as well as many other jurisdictions, that healthcare professionals may provide certain reproductive health services that some religious healthcare professionals object to on conscientious grounds, however, they do not have to provide those services, in case the provision of those services would violate their conscience. Such objecting healthcare professionals are required to transfer an assistance seeking person on to other healthcare professionals who will provide the required services in a timely manner. The underlying rationale for this procedural solution lies in this kind of reasoning: If *only* healthcare professionals are permitted to provide assistance but they are not obligated to do so, then their autonomy is not limited but the autonomy of those seeking assistance could potentially be unfairly limited. Hence the requirement on conscientious objectors to refer assistance seekers to colleagues who are prepared to oblige them. If individuals other than healthcare professionals are permitted to provide the assistance, then the autonomy of healthcare professionals and those seeking assistance is not limited. However, there could be legitimate concerns over how society could regulate the actions of non-healthcare professionals in this context in order to limit the risk of abuse.

The best way to balance these particular competing rights and interests is not yet clear. Conclusions about the best way to balance rest not so much on an unresolved conflict of values but more so on many logistical factors that cannot be addressed in the context of this Report. For example, is it the case that only healthcare professionals are competent to ensure that the conditions for permissible assisted suicide or voluntary euthanasia are met? Can the balancing be achieved through requiring some involvement of healthcare professionals (e.g. in cognitive capacity assessments, providing information, and prescription writing) but not excluding others from also playing some role (e.g. delivering a lethal drug mixed in orange juice for the individual to drink as a means of committing suicide)? Can the oversight necessary to ensure the conditions for a morally justifiable legal regime are met be achieved only through limiting those who can provide assistance to suicide or voluntary euthanasia to healthcare professionals? Would individuals who desire assistance be able to gain access to assistance if the permission was restricted to healthcare professionals and they were not required to provide assistance when asked? These and related questions require further information gathering and analysis of possible models of a regulated permissive regime.

The framework set out in the preceding sections is not sufficient to support a position on the issues of restricting the permission to provide assistance in suicide or voluntary euthanasia to healthcare professionals. The framework supports the conclusion that health care professionals should be permitted to provide assistance but requires further information-gathering and deliberation to take place before adopting a position on whether the permission to provide assistance should be limited to healthcare professionals.

### b. Slippery Slopes and the Protection of the Vulnerable

The arguments canvassed so far show that and why people have the right to take actions to end their lives when their lives are no longer worth living to them. The analysis demonstrates that the moral standard implicit in current medical practices implies that health care professionals may accede to the requests of their patients who, in these circumstances, decide that they wish to end their lives.

But, as we have seen, the morality of assisted dying does not settle the question of whether it should be decriminalized. The main reason that might block the passage from morality to legality has to do with the concern that, while it is possible to isolate precise conditions under which assisted dying might be morally justifiable, it would be difficult or impossible to design institutional mechanisms overseeing the decriminalized practice that would cleave precisely to those conditions. The fear is that assisted suicide and voluntary euthanasia will occur in circumstances that fall outside the morally acceptable range. For example, a fear might be that assistance with suicide or voluntary euthanasia will be administered to less than fully competent patients. A fear might be that the practice of assisting those who voluntarily choose to die might give rise to a situation in which people who fail to satisfy the voluntariness condition are put to death.^238^ In other words, some people may fear that decriminalizing morally permissible cases of assisted suicide and euthanasia will create a slippery slope that could lead to the practice being abused, and to assisted suicide and voluntary euthanasia occurring in morally impermissible circumstances.

Along with arguments surrounding the concept of *human dignity*, arguments invoking *slippery slopes* are among the most ubiquitous in debates about assisted suicide and euthanasia. Arguments concerning dignity attempt to show that assisted suicide and euthanasia are wrong in and of themselves, and independently of the consequences that they might have. Slippery slope arguments tacitly concede that certain cases of assisted suicide and euthanasia are morally permissible, but cast doubt on our ability to institutionalize them without producing catastrophic consequences.

Slippery slope arguments are ubiquitous in public debate. Hardly a day goes by without some radio talk show pundit, intent upon convincing listeners that a policy he opposes should not be adopted, argues that if we allow the policy in question, then another, far more noxious one, will inexorably follow in its train. This Report canvasses empirical evidence on this issue in Chapter 4. Academics arguing about controversial moral and policy issues are not immune to the lure of such arguments. For example, opponents of genetic testing and screening say that there is no way to control the slippery slope from therapeutic uses of these new techniques to eugenic ones.^239^ Similarly, opponents of assisted suicide argue that the decriminalization of this practice will elicit a slide into involuntary euthanasia.^240^

The ubiquity of such arguments, especially among academics, is surprising. After all, they are, in almost all cases, *logically invalid arguments*. When slippery slope arguments are invoked, it is almost always to change the subject. Rather than providing grounds for thinking that a proposed policy or principle is morally unacceptable, these arguments trade on the widely acknowledged inappropriateness of some other policy or principle, and then tar the matter under discussion with the acknowledged problems of the latter. These arguments do so by drawing some empirical or causal connection between the two. But as the subsequent analysis shows, these connections are almost impossible to vindicate.

Literature on the logic of argumentation distinguishes two basic forms of slippery slope argument. Both types are present in the assisted suicide and voluntary euthanasia debate. Some slippery slopes are *conceptual*. They claim the concepts used to set up criteria governing a practice are fuzzy, and that this conceptual vagueness will lead to the practice being abused. Others are *causal*. They claim that if a certain decision or policy is implemented, that could in and of itself be morally acceptable, causal mechanisms will be put in motion that will unavoidably lead to making other, much more morally dubious, decisions.

The first task at hand is to examine specifically conceptual slippery slope arguments against assisted suicide and euthanasia. According to such arguments, many of the concepts employed in order to create guidelines and criteria to limit the practice to morally acceptable cases are vague. A good example is the concept of *competence*. The argument of this Report proposes that the practice should be limited to competent individuals. Philosophical literature on the subject and clinical practice show the concept of competence is notoriously difficult to pin down. The line between competence and incompetence is ambiguous at best.

The conceptual slippery slope argument against assisted suicide and voluntary euthanasia takes the ambiguity of the concept as the premise of an argument that practicing assisted dying on incompetent people is unavoidable. The argument takes the form of what in philosophy is called a *sorites paradox*: for every competent person, there will be one just slightly less competent, where the difference between the two hardly seems significant enough to ground the claim that one is competent whereas the other is not. But then, there will be a person just slightly less competent than the second, and then another just slightly less competent than the third, and quickly, medically assisted dying is being practised on patients of whom it would be very difficult indeed to claim that they are competent. Frequently the spectre of the Nazis' murder of intellectually disabled people is invoked in order to indicate where this slippery slope would inexorably lead any society that decriminalized assisted dying in some form or shape.

The conceptual slippery slope argument against assisted suicide and voluntary euthanasia points to a real problem. But it is a problem that is ubiquitous across the full range of areas in which public policy and laws are enacted. Seeing it as a reason to rescind from enacting such laws and policies would lead to stasis. Consider a much less dramatic area of policy such as the determination of the age at which individuals can obtain a driver's license. There is no bright conceptual line that separates the competence and reliability of a person of 15 years and 364 days and a person of 16 years. The gain in competence from one day to the next is infinitesimally small.

Since it is not acceptable, as a matter of policy, not to grant people drivers' licences because of our inability to determine thresholds of competence with precision, the law establishes a line that is to some degree arbitrary. By fixing the minimal age requirement at 16, society attempts to do as well as possible in ensuring that only competent people get on the road, accepting a certain number of false negatives and false positives as an acceptable cost for allowing people to be able to drive.

The exponent of the slippery slope argument against assisted suicide and voluntary euthanasia will naturally disagree with the analogizing of cases of public policy (such as those just annulled with cases in which moral principles are in play). Whereas the former are amenable to cost/benefit reasoning, the latter, he or she will claim, are not. Thus, the argument might run, when the placing of an arbitrary line at one point rather than another, along a continuum, risks placing the defence of a principle on the wrong side of the line, we should avoid drawing lines altogether, and prescind from the impugned practice. This is a moral problem, no matter what the benefit of drawing the line somewhere. Thus, for example, if it is settled that stringency test ‘X’ accommodates requests for assisted dying, and that there exists a more stringent test ‘X + 1’, the space between ‘X’ and ‘X + 1’ can be cashed out in terms of lives lost that *ought* not to have been lost. Thus the most stringent test there is should be chosen: namely outright, or almost outright, prohibition.

This line of argument can be resisted in a number of ways. First, prohibition will not lead to the elimination of lives lost through assisted dying. It will rather mean that the practice will continue as it does in all jurisdictions where it is prohibited in the absence of *any* principle or institutional safeguard. Second, moral costs must be reckoned that flow both from permission and from prohibition – the moral costs of the latter are needless suffering and thwarting the wills of autonomous individuals.^241^ The exponent of the slippery slope argument against euthanasia and assisted suicide cannot, in other words, avoid assessing the costs of *not* drawing a line somewhere.

Finally, the vagueness of concepts can only be of limited use to the partisan of slippery slope arguments. For, though a concept like that of competence is ambiguous, it cannot be reasonably inferred that there are not clear, paradigm cases of competence and, correspondingly, that there are not paradigm cases of incompetence. The fallacy of the *sorites paradox* upon which the conceptual slippery slope is grounded claims there will not come a point when the succession of imperceptibles gives rise to cases in which it is known that it is no longer competent individuals being dealt with.

Causal slippery slopes, being based on empirical premises, are not amenable to logical refutation. Unless their empirical premises run counter to the laws of physics, they invoke real possibilities. It is, rather, that human decisions will give rise to other human decisions, and that, whereas the first set of decisions were morally acceptable – or only contestably morally unacceptable – the second set of decisions (unavoidable according to the slippery slope theorist once the first have been made) are clearly unacceptable. The inevitability of the second set of decisions is seen in this way to impugn the first.

The causal mechanisms invoked to make such arguments plausible are of a very different kind from those invoked in straight consequentialist reasoning. The argument based on such mechanisms is, the Panel submits, much more difficult to make good because these arguments imply that such mechanisms will hold sway, even when the possibilities are laid bare and steps are taken to counteract them. Consider two cases: the first is one in which a person has no moral qualms about the principles and decisions which may flow from an initial decision. She thinks that both are morally justified. When she adopts the second, it is not as a result of having fallen prey to the slippery slope. Rather, she is simply expressing her support for both ‘Decision 1’ and ‘Decision 2’, and for whatever principles underpin the two decisions.

In the second, an agent supports ‘Decision 1’, but has serious moral qualms about ‘Decision 2’. He is aware of the fact that there are empirical (psychological, social, institutional, etc.) mechanisms that may make it more likely that ‘Decision 2’ will come to seem more plausible to some, once ‘Decision 1’ has been taken. This person is aware of the risk of a slippery slope, but intent as he is to resist it, he will be at pains to put safeguards in place – both psychological and institutional – to make it less likely than it might otherwise have been that policy or ‘Decision 2’ will come to be adopted as a result of policy or ‘Decision 1’ having been adopted.

The supporter of slippery slope arguments offers a very difficult argument to respond to the agent in the second case. He will have to claim, not only that a slippery slope might be set in motion by the adoption of ‘Decision 1’, but that it will overpower whatever – legal, institutional, psychological, moral – resistances and safeguards responsible citizens and politicians, aware of the risks, are intent on putting in place in order to avoid the morally problematic decision being made. The partisan of the slippery slope thus has a formidable burden of argument to take up. Indeed, slippery slopes might fail to eventuate not only because the gradient of the slope might not be quite as steep as some think (the causal mechanisms linking a morally acceptable decision or policy to a morally problematic one that are invoked by partisans of slippery slope arguments may not be as reliable as hypothesized), but also – and this is the important point in the context of this argument – because there will be various kinds of obstacles built along the path of the slope that are the result of deliberate human intent.

In continuing to strike the theme of slippery slopes in the face of these considerations, the slippery slope theorist must take care not to overreach. Much already existing public policy takes precisely the form just described. Measures are taken, and watchdog institutions are put in place to guard against abuse. Under-discussed but crucial functions within liberal democracies such as auditors general and ombudsmen are just two such offices. There is no reason to think that this could not also be done in the case of assisted death.

Causal slippery slope arguments can play an important role in this context. Rather than being key elements in the *refutation* of moral/political positions, they can serve to *alert* decision-makers as to the kinds of safeguards that will have to be put in place, given the particular risks associated with a given decision. But this does not amount to the kind of role that practitioners and theorists who routinely employ slippery slope arguments consistently fill, which is to refute a position rather than contributing to designing safeguards that might maximize the benefits of its adoption while minimizing its costs. In designing the regulatory structure that would govern the practices of voluntary euthanasia and assisted suicide, some of the arguments of opponents of these practices should be attended to, not because they are convincing as refutations of the practices in question, but because they do point to potential risks against which prudent policy-makers will want to take steps to counteract.

Some slippery-slope arguments that can be found in the literature about assisted dying are good examples of fear-mongering rather than of a realistic assessment of the risks that might accompany the decriminalization of assisted dying. For instance, there is no evidentiary basis for the fear that the decriminalization of assisted dying would relax the inhibitions that medical professionals presently feel for resorting to assisted dying in all but the most extreme of contexts.

Other slippery-slope arguments suggest real risks against which institutional safeguards must be erected. The following risks deserve attention from prudent policy-makers and institutional designers.

First, the argument in this Report limits the justification of assisted dying to competent persons. Safeguards must be put in place that avoid ‘false positives’ to as great a degree as possible. That is, ways must be found to ensure that assisted dying is only provided to competent agents.

Second, opponents of the decriminalization of assisted dying often formulate the fear that it would lead to a reduction in resources for such practices as palliative care, and for adapting various social contexts and institutions to the (often quite costly) needs of persons with disabilities. The appetite for cost-cutting that characterizes most modern states, especially in the context of health-care, suggests that this fear is not unfounded. But again, the point is not to conclude from the observation that certain risks might possibly eventuate that the State ought to prohibit the practice of assisted death altogether. Rather, institutional safeguards must be put in place that would be effective in offsetting the risk in question. Thus, for example, it is conceivable that decriminalization of assisted death would be accompanied by legislation requiring that funding of programs such as palliative care, as well as policies aimed at addressing the needs of persons with chronic diseases and disabilities be maintained at certain levels. Such legislation could be accompanied by the creation of a watchdog body tasked with ensuring that funding commitments be respected, and more generally that the overall situation of palliative care, as well as of programs devoted to the needs of persons with disabilities and chronic diseases, not be worsened by the decriminalization of assisted dying. This body might for example take on a public education mandate, to ensure that the decriminalization not be accompanied in an erosion in public support for such programs, and in a coarsening of public attitudes with respect to the needs and interests of such persons.

The logic of slippery slope arguments, most charitably construed, is that there are certain risks that might accompany a policy, and that those risks are so grave, and/or society's capacity reliably to counteract them so limited, that it would be better not to enact the policy. This logic is however premised on a faulty assumption, namely that the status quo is itself without costs or risks, and that the only costs and risks to be factored into our deliberations concerning the desirability of moving away from the status quo are the ones that accompany the move away from the status quo. But this is never the case. Indeed, the problematic policy would not have been proposed had there not been a perception among policy-makers that the status quo was fraught with costs and harms. In the case of the issues before the Panel, that is most certainly the case. As has been pointed out in Chapter 2, the practice of assisted death presently occupies a shady area in which it is both prohibited, and the prohibitions against it sometimes unenforced. The result is that assisted dying presently goes on in various medical contexts in Canada, but it is governed not by transparent, reliable norms but by the private convictions of individuals. The present dispensation is fraught with all of the anxiety, uncertainty and needless suffering that attends any policy area governed by arbitrariness and lack of clarity rather than transparent, democratically enacted norms. The evidence adduced in Chapter 4 strongly suggests that jurisdictions that have liberalized laws concerning assisted death have not succumbed to the slippery slopes pointed to by opponents of liberalization. Opponents of decriminalization have not adequately taken into account the very real costs and harms that the present situation regarding assisted dying in Canada involves.

The Panel concludes that two important arguments that would, if successful, block the legal recognition of the *prima facie* moral right to assisted suicide, fail. There is no blanket moral objection to physicians assisting their patients in dying. Nor is the slippery slope that could lead from the decriminalization of assisted death in voluntary contexts to the acceptance of euthanasia in non-voluntary contexts as decisive as its proponents pretend. Rather than constituting a refutation of the argument in favour of a legal right to choose assisted death, they helpfully point us toward safeguards that must accompany that decriminalization, lest the safety and security of vulnerable Canadians be imperilled.

With this conclusion, the argument in favour of a legal right to choose assisted death is complete. We have shown that there is a strong autonomy-based argument in favour of the right that is not defeated by other constitutional values to do with safety and security, dignity, or the rights of third parties.

## 8. CONCLUSIONS

The Panel concludes:

1. That there is a moral right, grounded in autonomy, for competent and informed individuals who have decided after careful consideration of the relevant facts, that their continuing life is not worth living, to non-interference with requests for assistance with suicide or voluntary euthanasia.
2. That none of the grounds for denying individuals the enjoyment of their moral rights applies in the case of assisted suicide and voluntary euthanasia. There are no third-party interests, self-regarding duties, or duties toward objective goods that warrant denying people the right to assisted suicide and voluntary euthanasia. Prophesied undesirable social consequences are not sufficient to negate the right to choose assisted suicide and voluntary euthanasia. Rather, they should be taken into account in constructing the regulatory environment within which this right can be exercised.
3. That health care professionals are not duty-bound to accede to the request of competent and informed individuals who have formulated the uncoerced wish to die, but they may do so. If their religious or moral conscience prevents them from doing so, they are duty bound to refer their patients to a health care professional who will.

# Sub article: CHAPTER FOUR: INTERNATIONAL EXPERIENCE WITH LAWS ON ASSISTED DYING

**Published:** 2011-11

## 1. INTRODUCTION

This Panel has reached the conclusion that there are persuasive arguments in support of the development of a legally permissive regime with respect to assisted dying in Canada. Since Canada would not be the first country to embark on a path to a permissive regime, it is useful to examine and learn from the experiences of other nations. While the majority of countries throughout the world continue to regard assisted suicide and voluntary euthanasia as criminal offences, a small but significant number of jurisdictions now accommodate assisted suicide and/or voluntary euthanasia in certain circumstances. In this chapter, the Panel analyzes the experience of other countries in two ways.

First, the ways in which different countries have approached the issue of assisted dying are described.

Second, the Panel turns to the practical experience in these countries or states – in so far as there are available data – to see what happens in practice when assisted dying is, in some circumstances, allowed.

## 2. MECHANISMS FOR CHANGE TO LAW AND/OR PRACTICE

When discussing different legal mechanisms for changing law and practice with respect to assisted dying, it is important to remember that not all jurisdictions (countries or states) share a similar legal background. The starting point obviously dictates the range of potential routes to reform. Switzerland, for example, was able to develop a permissive regime because assisted suicide, under certain conditions, was not criminalized in the first place. The Netherlands, on the other hand, had to institute laws on both voluntary euthanasia and assisted suicide because both were, and still are, mentioned in the Dutch *Criminal Code.* Against this contextual backdrop, four major mechanisms have been used internationally to effect change: judicial decisions; prosecutorial charging guidelines; new or revised laws; and evolution of practice without legal change.

### a. Judicial Decisions

The courts have been used to effect change in two jurisdictions through imposing reduced sentences and recognizing defences to charges of voluntary euthanasia or assisted suicide.

In a 1973 case Dutch court recognized that a physician could lawfully be allowed to prevent serious and irremediable suffering, even if this meant shortening the patient's life. At that time, voluntary euthanasia and assisted suicide were punishable in all circumstances. In this particular case, the physician was found guilty of breaking the law but the court only meted out a more or less symbolic punishment. The decision provoked a wide-ranging legal discussion regarding the acceptability of active physician involvement in death. The underlying legal reasoning for accepting this involvement remained unclear until 1984, when the first voluntary euthanasia case reached the Dutch Supreme Court. It reasoned as follows:

1. As a general rule, voluntary euthanasia and assisted suicide are punishable since the Penal Code defines both activities as a crime.
2. However, when a physician is confronted with a conflict of duties he or she may invoke the so-called *defence of necessity*. A conflict of duties occurs when honouring a patient's request to die with dignity is the only available means to end unbearable and irremediable suffering.
3. The criteria for accepting this *defence of necessity* are to be derived from professional and medical ethical opinions formulated by the medical profession.^242^

This line of legal reasoning served as the foundation of the practice of voluntary euthanasia in the Netherlands until the country's 2002 Act came into force^243^ (this Act will be discussed in detail later in this chapter under the heading ‘New or Revised Laws’). The concept of euthanasia as a kind of medical exception was rejected by the Supreme Court. It reasoned that voluntary euthanasia could not be regarded as a normal medical procedure, like surgery. Thus, from 1973 to 2002, the Dutch *Criminal Code* remained unchanged, but the courts, through a series of decisions, established the parameters for the use of the defence of necessity in cases of voluntary euthanasia and assisted suicide.

The consequence of this court-based approach is that the *Criminal Code* remained unchanged, including the general prohibition of voluntary euthanasia and assisted suicide, but cases could go unpunished if they met the conditions stipulated in the court rulings. The result was a tough law compassionately applied. The downside of this policy was that its exact content remained undetermined, as only case rulings and no general guidelines were published. This resulted in an unsatisfactory situation whereby assisted dying was both accepted and prohibited, creating uncertainty and vagueness for both patients and physicians.

Another example of a courts-based approach is found in the US American state of Montana. On December 5, 2008, Justice Dorothy McCarter ruled that:

The Montana constitutional rights of individual privacy and human dignity, taken together, encompass the right of a competent terminally ill patient to die with dignity. That is to say, the patient may use the assistance of his physician to obtain a prescription for a lethal dose of medication that the patient may take on his own if and when he decides to terminate his life. The patient's right to die with dignity includes protection of the patient's physician from liability under the State's homicide statutes [the assisted suicide prohibition falls within the homicide statutes].

The Court recognizes compelling State interests in protecting patients and their loved ones from abuses, in protecting life in general, and in protecting the integrity and ethics of the medical profession. However, those interests can be protected [relying explicitly on the Oregon experience] while preserving a patient's right to die with dignity.^244^

As a result, the homicide statutes were declared unconstitutional as they applied to the plaintiffs and the application of those statutes to the plaintiffs was enjoined.

Justice McCarter's decision was appealed and, on December 31, 2009, the Supreme Court of Montana issued its ruling.^245^ The majority vacated the lower court's constitutional ruling (but not the result) on the basis that it was not necessary to make a constitutional ruling when the case could be decided on the basis of statutory analysis (in this case the Montana statutory consent defence). The majority of the Court held that:

[W]e find nothing in Montana Supreme Court precedents or Montana statutes indicating that physician aid in dying is against public policy. The ‘against public policy’ exception to consent was interpreted by this Court as applicable to violent breaches of the public peace. Physician aid in dying does not satisfy that definition. We also find nothing in the plain language of Montana statutes indicating that physician aid in dying is against public policy. In physician aid in dying, the patient – not the physician – commits the final death-causing act by self-administering a lethal dose of medicine.

Furthermore, the Montana Rights of the Terminally Ill Act indicates legislative respect for a patient's autonomous right to decide if and how he will receive medical treatment at the end of his life. The Terminally Ill Act explicitly shields physicians from liability for acting in accordance with a patient's end-of-life wishes, even if the physician must actively pull the plug on a patient's ventilator or withhold treatment that will keep him alive. There is no statutory indication that lesser end-of-life physician involvement, in which the patient himself commits the final act, is against public policy. We therefore hold that under §45-2-211, MCA, a terminally ill patient's consent to physician aid in dying constitutes a statutory defence to a charge of homicide against the aiding physician when no other consent exceptions apply.^246^

As this case involved interpretation of the Montana Constitution and criminal law (which falls under state jurisdiction), it cannot be appealed to any other court and, unless and until there is legislative reform, it stands as the law in Montana. Although opponents of the decision have asked the Montana legislature to ban assisted suicide,^247^ a recent poll indicated that 63% of Montana voters supported the Supreme Court decision and only 25% felt that the state legislature should ‘overturn the Supreme Court decision, making doctor-assisted suicide a crime.’^248^ Two competing bills were introduced in 2011 by Montana legislators (one seeking to prohibit physician-assisted suicide and the other seeking to establish a system within which assisted suicide would be permitted) but both died in the Standing Committee and so are ‘probably dead’.^249^

The experience of Montana points to another disadvantage of law reform through case law; there is no mechanism by which the state can monitor the results of the change. It has been reported in the press that there has been at least one physician-assisted suicide in Montana in 2010 and the President of Compassion and Choices was quoted by the Associated Press as saying that ‘[t]here are physicians in Montana who are implementing the law,’^250^ but declined to provide numbers or details, probably to protect the physicians and the individuals they have helped.

### b. Prosecutorial Charging Guidelines

The next mechanism has some similarities to the previous one, in that the *Criminal Code* remains unchanged, but a way is found to adopt a more accepting stance in at least some cases. The mechanism in question shifts the possibility of acceptance earlier in the process (at the stage of charging) through guidelines for the exercise of prosecutorial discretion in cases of voluntary euthanasia and assisted suicide. This approach has recently been taken in parts of the United Kingdom with respect to assisted suicide. The law as it operates in England and Wales will be discussed first and then the law as it operates in Scotland will be explained. It should be noted here that the Netherlands did make use of prosecutorial charging guidelines between 1994 and 2002. However, these were never the mechanism of change, rather they merely reflected the change that came about through case law. Furthermore, they have been superseded by the 2001 legislation and therefore will not be discussed in this section.

The law with respect to assisted suicide in England and Wales is contained in the Suicide Act 1961, section 2 (1), which says that ‘[a] person who aids, abets, counsels or procures the suicide of another, or an attempt by another to commit suicide, shall be liable on conviction on indictment to imprisonment for a term not exceeding fourteen years.’ The 1961 Act was primarily designed to decriminalize suicide itself. In so doing, the specific crime of assisting suicide was created, although the Act also indicates that no prosecution should take place without the agreement of the Director of Public Prosecutions (DPP).^251^ Much has changed in the legal landscape since these somewhat elderly provisions were enacted, not least the incorporation of the European Convention of Human Rights into UK law by means of the Human Rights Act of 1998. The passing of this act allows UK citizens to take full advantage of the rights contained in the Convention, many of which appear to have relevance to the assisted dying debate. More particularly, it allows citizens to challenge the compatibility of existing (and future) legislation with the Convention. As Michael Freeman has noted, it was ‘inevitable with the incorporation of the European Convention on Human Rights into English law that the ban on assisted suicide would be challenged.’^252^

The first such challenge arose in the case of Diane Pretty.^253^ Diane Pretty lost her case, but Freeman nonetheless argued ‘the time has come for a rethink, certainly of assisted suicide, and probably of all “end of life” decisions.’^254^ The opportunity to do so arrived some years later in the case of Debbie Purdy, which reached the House of Lords in 2009.^255^

Debbie Purdy suffers from multiple sclerosis (MS) and is currently wheelchair bound. She sought clarification from the DPP as to what they might decide in terms of prosecution should her husband travel with her – it is presumed to Switzerland – in order that she might have an assisted death. Assisted suicide is not a crime in Switzerland providing certain conditions prevail. Two questions were raised by her case. First, was the question of whether or not her husband would commit a crime by travelling with her to facilitate an act that is legal in that jurisdiction. Second, a question was raised about the clarity of the English prosecution's policies.

On the first question, while it might seem odd that travelling with someone to another country could be a constituent element of a crime, the House of Lords was in no doubt that it could be categorized as such.^256^ The second question was more complex. Although a Code of Practice for prosecutors already existed, the issue was whether or not it was sufficiently clear to satisfy the requirements of the European Convention on Human Rights and, in particular, article 8 (2).

As Lord Hope explained:

The Convention principle of legality requires the court to address itself to three distinct questions. The first is whether there is a legal basis in domestic law for the restriction. The second is whether the law or rule in question is sufficiently accessible to the individual who is affected by the restriction, and sufficiently precise to enable him to understand its scope and foresee the consequences of his actions so that he can regulate his conduct without breaking the law. The third is whether, assuming that these two requirements are satisfied, it is nevertheless open to the criticism that it is being applied in a way that is arbitrary because, for example, it has been resorted to in bad faith or in a way that is not proportionate.^257^

For Lord Brown, ‘with the best will in the world, it is simply impossible to find in the Code itself enough to satisfy the article 8(2) requirements of accessibility and foreseeability in assessing how prosecutorial discretion is likely to be exercised in section 2(1) cases.’^258^ The outcome of this case was a direction to the Director of Public Prosecutions that he should clarify and publicize the criteria that would be taken into consideration when deciding on whether or not to exercise his statutory prosecutorial discretion. Interim guidelines were produced in September 2009 and final guidance was issued in February 2010. The guidelines follow.

The sixteen public interest factors in favour of prosecution are:

1. The victim was under 18 years of age.
2. The victim did not have the capacity (as defined by the Mental Capacity Act 2005) to reach an informed decision to commit suicide.
3. The victim had not reached a voluntary, clear, settled and informed decision to commit suicide.
4. The victim had not clearly and unequivocally communicated his or her decision to commit suicide to the suspect.
5. The victim did not seek the encouragement or assistance of the suspect personally or on his or her own initiative.
6. The suspect was not wholly motivated by compassion; for example, the suspect was motivated by the prospect that he or she or a person closely connected to him or her stood to gain in some way from the death of the victim.
7. The suspect pressured the victim to commit suicide.
8. The suspect did not take reasonable steps to ensure that any other person had not pressured the victim to commit suicide.
9. The suspect had a history of violence or abuse against the victim.
10. The victim was physically able to undertake the act that constituted the assistance himself or herself.
11. The suspect was unknown to the victim and encouraged or assisted the victim to commit or attempt to commit suicide by providing specific information via, for example, a website or publication.
12. The suspect gave encouragement or assistance to more than one victim not known to each other.
13. The suspect was paid (by the victim, or those close to the victim) for his or her encouragement and/or assistance.
14. The suspect was acting in his or her capacity as a medical doctor, nurse, other healthcare professional, a professional care-giver (whether for payment or not), or as a person in authority, such as a prison officer, and the victim was in his or her care.
15. The suspect was aware that the victim intended to commit suicide in a public place where it was reasonable to think that members of the public might be present.
16. The suspect was acting in his or her capacity as a person involved in the management or as an employee (whether for payment or not) of an organization or group, a purpose of which is to provide a physical environment (whether for payment or not) in which to allow another to commit suicide.

The six public interest factors against prosecution are:

1. The victim had reached a voluntary, clear, settled and informed decision to commit suicide.
2. The suspect was wholly motivated by compassion.
3. The actions of the suspect, although sufficient to come within the definition of the crime, were of only minor encouragement or assistance.
4. The suspect had sought to dissuade the victim from the course of action that resulted in his or her suicide.
5. The actions of the suspect may be characterized as reluctant encouragement or assistance in the face of a determined wish on the part of the victim to commit suicide.
6. The suspect reported the victim's suicide to the police and fully assisted them in their enquiries into the circumstances of the suicide or the attempt and his or her part in providing encouragement or assistance.^259^

Debbie Purdy's case has been widely heralded as a victory for right-to-die campaigners. At least one newspaper was happy to categorise it as such, calling for law reform and arguing that ‘a significant blow has been dealt to the 1961 Act that makes an offence of “complicity” in suicide and so criminalises deeds that might otherwise be judged merciful.’^260^ The true importance of this case, however, actually lies in its potential to bring additional clarity to the law in England and Wales.

In Scotland, suicide has never been a crime; no specific crime of assisted suicide exists. This is not to say, however, that assisting a suicide would not fall under criminal law. The law of murder or culpable homicide (the Scottish equivalent of manslaughter) is the catch all for such behaviour.^261^ However, a major difference between the jurisdictions under consideration is that there is likely to be no crime committed in Scottish law should an individual accompany someone to another country – for example, Switzerland – where they then undertake an act that is lawful in that country. However, there is a dearth of case law in Scotland; this conclusion is derived from general principles rather than actual jurisprudence.

It is important to emphasize here that the charging guidelines do not apply to voluntary euthanasia (as elsewhere, in assisted suicide, the third party merely supplies the means for the individual to kill him- or herself, in the case of voluntary euthanasia, the third party directly acts to kill). Euthanasia is prohibited throughout the United Kingdom, not through statute, but rather through the common law. The common law makes it clear that consent is no defence against criminal charges, save in the case of rape where consent (or rather its absence) is central to the offence itself. An individual who kills another can, then, be prosecuted for the crime of murder. That said, despite the fact that there is a dearth of Scottish cases, it can be concluded that a murder charge, while possible, is unlikely in Scotland when the individual is motivated by compassion; the most likely charge would be culpable homicide, the Scottish equivalent of manslaughter (in Scotland, murder is not a form of culpable homicide, whereas in Canada murder and manslaughter are both forms of culpable homicide).

### c. New or Revised Laws

The most far-reaching mechanism for changing a regime with respect to assisted death is to decriminalize euthanasia and/or assisted suicide. The Netherlands, Belgium, Luxemburg and the US states of Oregon and Washington have chosen this route. In this section, the Dutch law – and what brought the Dutch to this law – is first described. There then follows a consideration of the legislative regimes in Belgium and Luxemburg, comparing them with the Dutch situation. Finally, the laws in Oregon and Washington are discussed.

It is worth noting that the Netherlands was not the first country to engage in the debate on euthanasia (although it was the first country to reform its law). This debate started in England, Germany and the USA somewhere in the middle of the 19th century when drugs became available to physicians that allowed them to influence the way people die. Interestingly, the first known proposal to decriminalize voluntary euthanasia was made in 1906 in the US state of Ohio.^262^ The Northern Territory of Australia was the first jurisdiction to decriminalize euthanasia but as it was overruled by the federal government it will not be considered further here.

#### i. The Netherlands

In the second half of the last century, Dutch society had rapidly become more secular and less divided along religious lines, framing the societal debate about life and death decisions in a different context. Responsibility for one's life, once safely in the hands of the church or the medical profession, has shifted back to the individual. Many Dutch people believe that they must be free to make their own decisions about their lives, including when and how their life should end. A large majority of the population is of the opinion that assistance in death should be available.^263^

The Dutch position on euthanasia and assisted suicide is the result of a long discussion both in society and in parliament on the acceptability of voluntary euthanasia and assisted suicide and the mechanisms for social control of these practices. As stated above, the shape of policy at the beginning of this discussion was more or less determined by case law, but the exact content of the policy remained undetermined. This resulted in an unsatisfactory situation whereby assisted dying appeared to be both accepted and prohibited at the same time. The Dutch government tried to end this ambiguity by implementing a uniform notification procedure in 1990, hoping to persuade physicians to bring voluntary euthanasia cases to the attention of the authorities.^264^ This notification procedure helped to raise the notification rate from 18% in 1990 to 41% in 1995. A 1995 study into end-of-life decision-making revealed that doctors who did not report cases of voluntary euthanasia had usually acted according to the established criteria.^265^ Why then did they fail to report their actions to the public prosecutor? The main reason seems to have been that – despite the fact that they had exercised due care – they felt they were being treated as criminals precisely *because* they had to report to the Public Prosecution Service, and then faced long periods of uncertainty, during which time they were formally murder suspects.

The government therefore tried to lower the number of unreported cases further by developing a new notification procedure, in which much of the assessment of the physician's behaviour was undertaken outside of the legal system.^266^ In 1998, five regional multidisciplinary assessment committees were created to assess all reported cases of voluntary euthanasia and assisted suicide. These committees consisted of a lawyer, a physician and an ethicist. The outcome of their assessment was to be regarded merely as advice to the prosecutor. The effect of this change in procedure on the notification rate was a rise in the reporting percentage to 54% in 2001. This was better than 41% in 1995, but far from satisfactory. The continued relatively low numbers of reporting led the government to change the role of the assessment committees. Following the passage of the Termination of Life on Request and Assisted Suicide (Review Procedures Act) (the Dutch Act) in 2001 (and coming into force in 2002)^267^ the ruling of the committee is no longer just advice to the prosecutor, but is the final judgement in all cases in which the criteria are met. Only if the criteria are not met is the report handed over to the prosecutor. After this change in the law, physicians reported in 80% of cases a considerable improvement.^268^

The Dutch government also wanted to create a system of assessment that did not inevitably involve the prosecutor. The coalition parties in the government, therefore, developed a proposal giving immunity from prosecution to those physicians whose cases had come before a review committee and where the committee had decided that the physician had acted with due care. This immunity from prosecution was regulated in the Dutch Act. The Dutch Act states that, although voluntary euthanasia and assisted suicide remain, in principle, criminal offences, physicians will not be prosecuted if they have reported to the committee and the committee finds that they have acted with due care.^269^ The Public Prosecution Service in these cases is no longer notified and the physician cannot be prosecuted. Only if the review committee finds that they have not acted with due care, will it then contact the Public Prosecution Service which, in turn, will decide whether or not to press charges.

The ‘due care criteria’ that a physician has to observe when performing voluntary euthanasia or assisted suicide, as they are set out in the Dutch Act, are as follows:

The attending physician must:

1. be satisfied that the patient has made a voluntary and carefully considered request;
2. be satisfied that the patient's suffering was unbearable, and that there was no prospect of improvement;
3. have informed the patient about his situation and his prospects;
4. have come to the conclusion, together with the patient, that there is no reasonable alternative in the light of the patient's situation;
5. have consulted at least one other, independent physician, who must have seen the patient and given a written opinion on the due care criteria referred to in 1–4 above; and
6. have terminated the patient's life or provided assistance with suicide with due medical care and attention.

Physicians have to decide for themselves whether they can meet the due care criteria in a specific situation. The patient's decision must be genuinely voluntary; doctors therefore have to be sure that the patient's request is not the result, for example, of family pressure. Whether the suffering is unbearable or not is, of course, a subjective judgement. Every individual has their own limits in terms of how much pain and suffering and loss of self they can bear. The prospect of improvement, however, can be assessed more objectively in medical terms. Recent advances in palliative care have made it even more relevant to discuss alternative options with the patient. The obligation to consult a second independent physician is an essential part of the review system. The second physician has to see the patient in person and submit his or her opinion to the review committee in writing. Subsequently, the committee examines whether the attending physician exercised due care in reaching their decision and in their actions.

It should be added that, under the Dutch Act, no physician is obligated to fulfill the request of a patient, even when the criteria are met, because neither voluntary euthanasia nor assisted suicide is regarded as a normal medical procedure. If a physician is a conscientious objector to assistance in dying, their refusal to comply with the request will be respected. What is expected, however, is that the physician assists the patient in finding another physician who has a different view of voluntary euthanasia or assisted suicide. It goes without saying that such a referral should not be left until the very last phase of the patient's life. It is also expected that the objecting physician will discuss his or her views on voluntary euthanasia and assisted suicide with the patient at an appropriate time, preferably early in the palliative phase.

Importantly, terminal illness has explicitly been rejected as a condition necessary for granting a request for voluntary euthanasia or assisted suicide. The reason for this has always been that unbearable suffering without the prospect of improvement is not restricted to the terminal phase of a disease.

While Dutch law has made some physician-assisted dying permissible, it has also created new boundaries. It is clear from the criteria that the physician plays a crucial role. The patient has to make a voluntary and well-considered decision, and the physician must consult the patient at every step. Ultimately, it is the physician's actions that are under scrutiny and they bear final responsibility. This sets limits on a patient's right to choose. Patients have no right to voluntary euthanasia in the Netherlands and physicians are not obligated to grant a request for voluntary euthanasia. The result of this is the medicalization of end-of-life decisions, since whether or not physician-assisted death is justifiable, in whatever form, becomes largely a matter of medical judgement. For example, requests from elderly people who are tired of life and for whom life has lost all meaning, but who do not suffer from any serious illnesses, cannot be complied with under the present framework. This was confirmed in the 2002 ruling of the Supreme Court in the Brongersma case.^270^ In addition, physicians remain reluctant to follow advance directives containing a written request for euthanasia in the case of incompetent (for example, demented) patients.^271^ By emphasizing the role of the physician in the regulatory framework for voluntary euthanasia and assisted suicide, requests that are exclusively grounded in the patient's evaluation of his or her life (vs. the physician's assessment as well) may not be accommodated. Alternative systems that put greater emphasis on patient autonomy might be better equipped to satisfy such requests.

#### ii. Belgium

In Belgium, the legal conditions for the lawful practice of voluntary euthanasia can be found in the Belgian Act on Euthanasia of May 28, 2002 that came into effect on September 23, 2002 (Belgian Act).^272^ It is important to note that, while the Dutch Act aimed to codify existing practices, the Belgian Act primarily aimed to modify the behavior of physicians.^273^ It was hoped that Belgian physicians would abandon their practice of actively ending the lives of patients without request (LAWER), as was shown to be happening by the first empirical data reported out of Flanders.^274^

Both the Dutch and the Belgian Acts regulate the practice of voluntary euthanasia, defined as the intentional termination of one person's life, at their request, by another person. Whereas the Dutch Act treats voluntary euthanasia and assisted suicide in the same way and regulates both practices, the Belgian Act does not consider assisted suicide at all. While the Dutch *Criminal Code* criminalizes assisted suicide (in section 294), the Belgian *Criminal Code* does not. Therefore, at first there was some lack of clarity with respect to the status of assisted suicide in Belgium.^275^ This has since been clarified by the Federal Control and Evaluation Commission which ‘has accepted cases of assisted suicide as falling under the law’.^276^ One striking similarity between the Dutch and Belgian Acts is that both are concerned with assisted deaths brought about solely by physicians. In both countries voluntary euthanasia is accepted if, and only if, performed by a physician and, as shown below, in both countries certain medical criteria must be met for voluntary euthanasia to be lawful. In effect, both countries countenance only medicalized voluntary euthanasia. This is also reflected in the fact that in The Netherlands the physician performing voluntary euthanasia should have an established treatment relationship with the patient,^277^ although this is less clear in the case of Belgium.

Two characteristics are central to the lawful practice of voluntary euthanasia in Belgium: it must follow a competent patient's request; and eligibility is determined by the patient's medical condition. With respect to the first requirement, the Belgian Act is more detailed than the Dutch one, at least at first glance. The Belgian Act requires the request to be voluntary, considered, repeated and made in writing, free from external pressure and of a durable nature,^278^ but the Dutch Act only requires the request to be voluntary and well considered.^279^ However, if one takes into account the way in which the Dutch review committees judge the request of the patient, it is clear that many of the criteria that are specified in the Belgian Act also apply *de facto* if not *de jure* in the Netherlands.

The second important requirement concerns the suffering of the patient who requests euthanasia. The Belgian Act requires the patient to be ‘in a medically hopeless condition of continuous and unbearable physical and mental suffering that cannot be alleviated and that is resulting from a serious and incurable disorder caused by illness or accident.’^280^ The Dutch Act states that the physician who has performed voluntary euthanasia or assisted suicide should be convinced that the patient is suffering unbearably and hopelessly.^281^ It is generally accepted that the first element is a subjective one, whereas the second is more objective. In both countries, a patient's suffering does not have to be physical, but it should originate from a medical condition. As in the Netherlands, terminal illness is not a necessary condition for voluntary euthanasia to be lawful in Belgium.

#### iii. Luxemburg

In March 2009, Luxemburg decriminalized both voluntary euthanasia and assisted suicide.^282^ Unlike the legislation in the Netherlands and Belgium, the legislation was not introduced by the Luxemburg government; rather, it was proposed by two members of Parliament. Again, as in Belgium and the Netherlands, physician-assisted dying is medicalized. Article 1 states that voluntary euthanasia is an act performed by a physician, which intentionally ends the life of a person at the express and voluntary request of that person. The definition of assisted suicide in the same article runs along the same lines, except that the termination of life is undertaken by the patient.

The same conditions apply to voluntary euthanasia and assisted suicide in Luxemburg. The conditions specified resemble the Dutch criteria in many respects. The Luxemburg law stipulates that the request should be made voluntarily and carefully, it should be repeated and should not result from external pressure.^283^ The patient's medical situation must be hopeless and the patient must report constant and unbearable mental or physical suffering with no prospect of improvement.^284^ Again, the patient does not have to be in the terminal phase of an illness.

It is not only within Europe that legislative changes have occurred in this area. Increasing public engagement with the issue – as well as an increasing recognition of the primacy of personal autonomy that continues to dominate both ethics and law– have also led to initiatives to change the law in the United States.

#### iv. Oregon

In November 1994, the Death With Dignity Act was passed by Oregon voters through a ballot initiative.^285^ The Act faced, and ultimately survived, a host of challenges including: court challenges aimed at having the Act declared unconstitutional;^286^ a ballot initiative aimed at repealing the Act;^287^ federal legislative efforts to effectively block the law;^288^ and a federal policy directive aimed at preventing physicians from providing assistance under the Act.^289^ The Act was finally enacted by the Oregon legislature in 1997.

The Death With Dignity Act permits physician-assisted suicide provided a number of substantive and procedural requirements have been met, including:

- The person must be a capable adult (18 years of age or older), a resident of Oregon, and terminally ill (given a prognosis of less than six months to live).
- The decision must be voluntary and informed.
- The diagnosis, as well as the competence and voluntariness of the request, must be confirmed by two physicians.
- The request must be signed and witnessed by two people (one of whom cannot be a relative or in other ways in a position of potential conflict of interest).^290^

#### v. Washington State

In November 2008, the Death with Dignity Act was passed by Washington State voters through a ballot initiative;^291^ the Act came into effect in March 2009.^292^ The Act in Washington State is similar to the Act in Oregon:

An adult who is competent, is a resident of Washington State, has been determined by the attending physician and consulting physician to be suffering from a terminal disease, and who has voluntarily expressed his or her wish to die, may make a written request for medication that the patient may self-administer to end his or her life in a humane and dignified manner in accordance with this chapter.^293^

### d. Evolution of Practice Without Legal Change

For the purposes of this Report it is useful to look at countries where the need for decriminalization was absent as the *Criminal Code* of that country never contained a prohibition of assisted suicide in the first place. In Switzerland, there has been an evolution of practice without legal change.

Unlike other European countries that have decriminalized voluntary euthanasia and/or assisted suicide, Switzerland does not have a specific statute in this area. Rather, the legal position is based on the Swiss *Criminal Code*. While article 114 of the Code makes it a criminal offence to kill someone even ‘upon the latter's earnest and urgent request’, article 115 criminalizes those who assist a suicide unless they act from honourable motives. An individual who assists another to commit suicide must show that they did not act for ‘self serving ends’. In evidence to the UK House of Lords Select Committee on the Assisted Dying for the Terminally Ill Bill,^294^ the Swiss Ministry of Justice explained that ‘self serving ends’ would cover assisting a death ‘to satisfy his own material or emotional needs …. the possibility of eliminating some major problem for the family, or other motives such as gaining an inheritance, relieving himself of the burden of supporting the individual … or eliminating a person he hated’.^295^

Another feature that distinguishes the situation in Switzerland from other jurisdictions which have moved to somewhat permissive regimes with respect to assisted suicide – European and non-European – is that, beyond prescribing the lethal substance, there is no requirement that doctors are involved in the suicide. Indeed, as the UK House of Lords Select Committee noted, ‘the vast majority of assisted suicides which take place in Switzerland are not directly supervised by doctors.’^296^ The arguments offered by clinicians, therefore, and especially those in palliative care^297^ that focus on the allegedly detrimental effect that decriminalization of assisted suicide would have on the doctor/patient relationship, clearly do not apply in Switzerland (nor need they apply in any other jurisdiction that decides to decriminalize assisted suicide). Equally, ‘[b]ecause the Swiss Penal Code does not link assisted suicide specifically with terminal illness or suffering as the result of ill-health, it does not specify any medical conditions under which assistance with suicide may be given.’^298^

Although there is no requirement that doctors be directly involved in assisting a suicide, which is often undertaken by one of the four voluntary organisations that exist in Switzerland, doctors are on occasion asked by their patients to assist in their death. The Swiss Academy of Medical Sciences has, therefore, issued guidelines which declare that physician-assisted suicide is ‘not part of a doctor's task’ yet ‘consideration of the patient's wishes is fundamental for the doctor-patient relationship.’ It concludes that ‘this dilemma requires a personal decision of conscience on the part of the doctor. The decision to provide assistance in suicide must be respected as such.’^299^

The Swiss position is summed up by Guillod and Schmidt who confirm that ‘assisted suicide is a crime only when four elements can be shown: a suicide was committed or attempted; a third party encouraged or helped in the suicide; the third party acted on selfish grounds; the third party acted deliberately (intent)’.^300^ The first two of these criteria are referred to as ‘objective’ and the latter two as ‘subjective’. All suicides, including those that are assisted, must be reported and will be ‘investigated on the spot by the authorities in conjunction with a forensic medical officer.’^301^ Research into one of the voluntary organizations that exist in Switzerland (EXIT Deutsche Schweiz), while concluding that the numbers of assisted suicides was increasing year on year, also found that the indications for assisted suicide had not been relaxed over the years and that reporting rates appeared to be 100%.^302^

Nonetheless, although it is lawful to assist in a suicide within the framework described, it is also important to note that there is no right to assisted suicide in Switzerland. As Guillod and Schmidt comment:

Article 115 of the Penal code is a criminal provision and, therefore, cannot create a right to assisted suicide. It merely recognizes the liberty to request assisted suicide and leaves it to each third party (whether a health-care professional or not) to accept such a request.^303^

This apparently benign picture of Swiss law, however, disguises the debate that has continued in that country for some years. Two particular aspects of the debate are worthy of brief consideration here. First, is the question of eligibility for an assisted death. As the above has shown, Swiss law does not require that the individual be terminally ill to be eligible for assisted suicide, although a recent agreement between EXIT and the Zurich chief prosecutor – the first such agreement – will ‘regulate the particulars of assisted suicide, including the use of deadly sodium pentobarbital,’^304^ and it is reported that Swiss authorities want to limit the availability of assisted suicide to those who are terminally ill.^305^ Moreover, there is apparently concern about the number of foreigners travelling to Switzerland to take advantage of the law.^306^ While it is anticipated that there may be legislative reform in Switzerland, limitations on citizenship or residency do not as yet apply. It is arguably paradoxical that, while Swiss law seems to work well, there is so much current activity concerning possible legal reform. It appears that at least part of the current debate is driven by the accessibility of assisted suicide to those who live outside Switzerland in countries that prohibit the activity. However, should the anticipated legal reform become a reality, it is plausible that it will not simply focus on so-called ‘assisted suicide tourism’, but may also involve a reconsideration of the eligibility criteria.

## 3. ELEMENTS OF REGULATED PERMISSIVE REGIMES

Clearly, the mechanisms discussed above differ not only in legal position and latitude but also in the requirements put on physicians and patients. These differences with respect to the elements of regulated permissive regimes, are summarized in Table 1.

**Table 1:** 

	NL	B	L	CH	Oregon	Washington
Which type of assisted dying is regulated?	VE, AS	VE, [AS]	VE, AS	AS	AS	AS
Who is allowed to aid?	Physician	Physician	Physician	Lay person	Physician	Physician
Provision for euthanasia through advance directives?	yes	yes	yes	no	no	no
Limits on health condition	yes	yes	yes	no	yes	yes
Consultation required?	yes	yes	yes	no	yes	yes

What becomes immediately apparent when looking at this table is that, in almost all jurisdictions (with the exception of Switzerland), assisted dying is accepted only if performed by a physician. This convergence may originate from the fact that in all these jurisdictions limits are put on the health condition of the patient who wants to be assisted in dying. These limits vary from terminal illness to suffering without prospect of being relieved, but since physicians are capable of evaluating such conditions, at least to a certain extent, it would seem obvious that they are the ones best situated to provide assistance. An additional reason for the convergence may be that in all these jurisdictions there are mechanisms in place that only allow the prescription of drugs by physicians. It may also be the case that the societies involved perceive putting aid in dying in the hands of physicians as an additional safeguard against abuse. Whatever is true of these hypotheses, the example of Switzerland highlights that there is no inevitable logic that has led to the current mechanisms. Assistance in dying could also be regulated with the limited involvement of physicians and without putting limits on the health condition of the person requesting the aid.

An additional and very important common feature of regimes in countries that have decriminalized assisted dying is an oversight structure. These structures have different formats and obligations, but a common goal is that, through their implementation, the state and the public should be able to have insight into, and control over, the practice of assisted dying.

A striking similarity between the Dutch, Belgian, and Luxemburg Acts is that reporting of the case to a review committee is an explicit legal condition. The physician who does not respect this obligation commits a criminal offence. In all three countries, review committees are created as separate, independent bodies. Their tasks are not only to keep an inventory of cases but also to evaluate the cases reported to them and to judge whether the requirements for due care were met. The composition of these bodies is therefore multidisciplinary in all countries and, in Belgium they include representatives of, for instance, different religious perspectives.

The role of the bodies tasked with oversight in Oregon and Washington State are slightly different, as they are not charged with judging individual cases. Their task is to collect data and report on the practice. It is not surprising that the position and the composition of these bodies in these states differ from their equivalents in the Netherlands, Belgium, and Luxemburg. In Oregon and Washington State the oversight bodies are part of state administration. As required by Oregon law, data on activity under the Act has been kept since 1998 and is reported publicly on an annual basis.^307^ As in Oregon, data must be collected in Washington State and publicly reported on an annual basis.^308^

## 4. PRACTICAL EXPERIENCE

### a. Data on Voluntary Euthanasia and Assisted Suicide

For the purposes of this Report, it is interesting to see what legal mechanisms have been used with respect to assisted dying in different jurisdictions. It is also helpful to know what has happened in countries when such mechanisms have been introduced. Given the lack of publicly accessible information for some of the countries or states discussed in this document, only data from the Netherlands, Belgium, Switzerland, Oregon, and Washington can be presented, starting with the Netherlands as it is the country most extensively studied to date and most referred to in debates about voluntary euthanasia and assisted suicide.

#### i. Netherlands

In the Netherlands, systematic empirical research did not start as an oversight mechanism after the introduction of a permissive regime. Rather, this research helped to pave the way for the changes in the reporting procedure which eventually led to the Dutch Act. As such, they are a unique feature of the process of decriminalizing voluntary euthanasia in the Netherlands. Large scale, nationwide studies were undertaken in 1990, 1995, 2001, 2005.^309^ A new round began in the fall of 2010.

This process started in 1990 when the government decided that legislation on voluntary euthanasia should be deferred until the findings of a commission appointed to conduct research on the frequency and characteristics of voluntary euthanasia, physician-assisted suicide, and other medical end-of-life decisions in the Netherlands was available.^310^ The method of these studies will be described here in somewhat more detail as it has been used elsewhere.

The frequency and characteristics of end-of-life decisions were studied using death certificate studies in 1990, 1995, 2001, and 2005.^311^ Stratified samples of cases were drawn from the central death registry of Statistics Netherlands, to which all deaths in the Netherlands are reported. Deaths were assigned to strata, according to the likelihood that the death was preceded by an end-of-life decision. Higher sampling proportions were drawn from strata where the likelihood of an end-of-life decision was higher. All physicians attending deaths for which the cause of death did not preclude an end-of-life decision (such as a car accident resulting in instant death) received a written questionnaire. The anonymity of physicians and patients was guaranteed. This approach resulted in high response rates: 76% in 1990, 77% in 1995, 74% in 2001, and 78% in 2005.^312^

In 1990, 1.7% of all deaths were preceded by voluntary euthanasia, as compared to 2.4% in 1995, 2.6% in 2001 (see Table 2). This trend reversed in 2005, when again 1.7% of all deaths were the result of voluntary euthanasia (approximately 2325 cases). Physician-assisted suicide occurred with much lower frequency than voluntary euthanasia in each year; for example, in 2005 it accounted for only 0.1% of all deaths (approximately 100 cases annually). An important factor in explaining the decline in the frequency of voluntary euthanasia and physician-assisted suicide in the period 2000–2005 may be an increased use of other options to relieve the patient's suffering, such as terminal sedation, which increased from 5.6% of all deaths in 2001 to 8.2% in 2005. The table below further shows that the frequency of the ending of life without an explicit patient request (LAWER) decreased from 0.8% of all deaths in 1990 to 0.4% in 2005 (approximately 550 cases). Further analysis of these cases shows that they typically involve patients who are very close to death and are presently incompetent but where there has been an earlier discussion about the hastening of death with them and/or their relatives, and where opioids were used to end life.^313^ The most recent study also showed that about one third of these cases can also be described as terminal sedation: cases in which high dosages of sedatives were given without hydrating the patient.^314^

**Table 2:** 

	1990	1995	2001	2005
Annual number of deaths	128,824	135,675	140,377	136,402
	%	%	%	%
Voluntary euthanasia	1.7	2.4	2.6	1.7
Assisted suicide	0.2	0.2	0.2	0.1
LAWER*	0.8	0.7	0.7	0.4
Total	2.7	3.3	3.5	2.2

* Life-terminating Acts Without Explicit Request of the Patient (LAWER).

The studies show in great detail the kind of doctors and patients involved in the decisions described. In interviews, 57% of doctors said that they had performed voluntary euthanasia or assisted suicide. A further 32% reportedly could conceive of situations in which they would be prepared to do so.^315^ General practitioners are involved in 87% of voluntary euthanasia cases and nearly all cases of assisted suicide. About 9% of voluntary euthanasia cases are performed in hospital.^316^ With respect to the patients involved, the studies showed that in two thirds of cases of voluntary euthanasia or assisted suicide, the patients were in the terminal stages of different illnesses, most often cancer (84%).^317^ The estimated shortening of life was less than one week in 46% of cases; only 8% of patients had a life-expectancy of more than one month.^318^ The number of voluntary euthanasia requests decreased from 9700 in 2001 to 8400 in 2005.^319^ Most requests for euthanasia are not complied with. Given the evidence contained in table 2, requests were met in 2325 cases by performing voluntary euthanasia and in 100 cases by assisting in suicide. Therefore, only approximately three out of ten requests for assistance in dying were granted. In about half of the cases where the patient's request was not followed by voluntary euthanasia or assisted suicide, the patient died before a final decision whether or not to perform voluntary euthanasia or provide assistance with suicide had been made. In the other half, the physician refused to grant the request. The most frequently mentioned reasons for not granting the request were: the patient died before the request could be fulfilled (39%), the request was not well-considered (18%), suffering was not unbearable (16%), and the patient withdrew the request (10%).^320^

#### ii. Belgium

There has now also been a series of studies in Belgium in which, by and large, the same method was applied. The third survey, with data from 2007, showed that the enactment of the Belgian euthanasia law was followed by an increase in all types of end-of-life decisions, with the exception of the use of lethal drugs without the patient's explicit request.^321^ Taking the longer view, between 1998 and 2007, the incidence of voluntary euthanasia went from 1.1% to 1.9% of annual deaths, and of assisted suicide from 0.12% to 0.07%. The incidence of the LAWER cases in 2007 was almost identical to voluntary euthanasia at 1.8%. This constitutes a sharp reduction of LAWER cases, when compared with the situation in 1998 when ending of life without the patient's explicit request occurred reportedly in 3.2% of cases.

#### iii. Switzerland

In Switzerland, between 1990 and 2000, the organization EXIT assisted in 748 suicides among Swiss residents (0.1% of total deaths, 4.8% of total suicides).^322^ Compared to EXIT (E), Dignitas (D) provided more assistance to non-residents (D: 91%; E: 3%), younger persons (mean age in years D: 64.5; E: 76.6), and people suffering from fatal diseases such as multiple sclerosis (MS) and amyotrophic lateral sclerosis (ALS), (D: 79%; E: 67%). The number of women and the proportion of older people suffering from non-fatal diseases among suicides assisted by EXIT Deutsche Schweiz has increased since the 1990s.^323^

#### iv. Oregon

As required by law, data on activity under the Oregon Act have been kept since 1998 and are reported publicly on an annual basis. The following is taken from the most recent official summary of the data:

- As of January 7, 2011, 96 prescriptions for lethal medications had been written under the provisions of the DWDA [Death with Dignity Act] during 2010, compared to 95 during 2009. Of the 96 patients for whom prescriptions were written during 2010, 59 died from ingesting the medications. In addition, six patients with prescriptions written during previous years ingested the medications and died during 2010 for a total of 65 known 2010 DWDA deaths at the time of this report. This corresponds to 20.9 DWDA deaths per 10,000 total deaths.
- Of the 65 patients who died under DWDA in 2010, most (70.8%) were over age 65 years; the median age was 72 years. As in previous years, most were white (100%), well-educated (42.2% had at least a baccalaureate degree), and had cancer (78.5%).
- Most (96.6%) patients died at home; and most (92.6%) were enrolled in hospice care at time of death. Most (96.7%) had some form of health care insurance, although the number of patients who had private insurance (60.0%) was lower in 2010 than in previous years (69.1%), and the number of patients who had only Medicare or Medicaid insurance was higher than in previous years (36.7% compared to 29.6%).
- As in previous years, the most frequently mentioned end-of-life concerns were: loss of autonomy (93.8%), decreasing ability to participate in activities that made life enjoyable (93.8%), and loss of dignity (78.5%).
- Since the law passed in 1997, 525 patients have died from ingesting mediations prescribed under the Death with Dignity Act.^324^

#### v. Washington State

As in Oregon, data must be collected in Washington State and publicly reported on an annual basis.^325^ The official summary of the data on the 2010 calendar year is as follows:

In 2010, medication was dispensed to 87 individuals (defined as 2010 participants):

Prescriptions were written by 68 different physicians
Prescriptions were dispensed by 40 different pharmacists

Of the 87 participants in 2010:

72 individuals have died

51 of these people died after ingesting the medication

15 of these people died without having ingested the medication

For the remaining 6 people who died, ingestion status is unknown

Status is unknown for the remaining 15 people

Of the 72 participants in 2010 who have died, their characteristics and underlying illnesses include:

Age range between 52 and 99 years

94% lived west of the Cascades

78% had cancer

10% had neuro-degenerative disease, including Amyotrophic Lateral Sclerosis (ALS)

12% had heart disease or other illnesses

88% had private, Medicare or Medicaid or a combination of health insurance

Of the 72 participants in 2010 who have died, Death Certificates were received for 61 of these individuals. Their characteristics include:

95% were white, non-Hispanic

51% were married

62% had at least some college education

Of the 72 participants in 2010 who have died, After Death Reporting Forms were received for 67 of these individuals. Their end-of-life concerns include:

90% were concerned about loss of autonomy, 64% about loss of dignity, and 87% about losing the ability to participate in activities that made life enjoyable

Of the 51 participants in 2010 who ingested the medication and died:

90% were at home and 84% were enrolled in hospice care when they ingested the medication.

No complications of ingesting the medication were reported

Emergency Medical Services (EMS) were not called for intervention after ingestion of the medication by any participant.^326^

### b. Slippery Slopes?

Two aspects of the Dutch experience need some further discussion here, as they are frequently referred to as evidence of a descent down a slippery slope, specifically with respect to vulnerable groups such as the elderly, the disabled, and incompetent patients.

With respect to euthanasia and incompetent patients there are two issues to be discussed: LAWER and the so-called ‘Groningen protocol’. The publication of the LAWER cases in the Netherlands described above created a new dimension in the Dutch euthanasia debate. Since the middle of the 1980s, the debate had focused on voluntary euthanasia and assisted suicide with the explicit request of the patient as the central feature. This in part had been a deliberate attempt to narrow the discussion because it was felt that consensus was most likely to be achieved in cases of this sort. The Dutch even changed their definition of euthanasia to include only those cases in which there was an explicit request from the patient. The societal impact of the LAWER cases, however, was to broaden the discussion. In particular, the results may have created the impression that the Dutch started hastening the end of life on request and ended up accepting non-voluntary ending of life; the so-called slippery slope often referred to by opponents of permissive legal regimes.^327^ This, however, is not necessarily true, as it is simply not possible to know whether LAWER cases occurred more or less often in the past. What is known is that the occurrence of such cases decreased in the Netherlands between 1991 and 2005. It is also known that their prevalence was higher in Belgium which did not tolerate voluntary euthanasia for many years.^328^ In 2003, the results of a European study conducted in Belgium, Denmark, Italy, the Netherlands, Sweden and Switzerland were published.^329^ The study design was the same as that used in previous studies in the Netherlands and in Belgium. In countries with a restrictive regime for assisted suicide and euthanasia, the incidence of non-voluntary cases was higher than of voluntary ones, as opposed to countries with permissive regimes. Apparently, therefore, the incidence of non-voluntary cases of assisted death is independent of the permissibility of euthanasia and assisted suicide. It may even be the case that an open and liberal policy leads to a reduction in non-voluntary assisted dying.

As was stated above, after the narrowing of the definition of euthanasia in1985 to active voluntary euthanasia, societal debate in the Netherlands concentrated on competent patients. In 2005, however, the publication of the so-called ‘Groningen protocol’ changed this as this protocol provided for the active ending of life of some newborns.^330^ After a thorough discussion with the Dutch Paediatric Association, the government responded to this local protocol by issuing a ruling that led to a prosecutorial guideline and to the creation of a committee of experts to advise the Prosecutor in individual cases.^331^ This has been cited as evidence of a slide down a slippery slope. However, a number of responses can be made to those who assert a slide. First, the ruling did not change the criminal law; active ending of life without request remains a criminal offense. Second, since the establishment of the committee in March 2007, only one case has been reported.^332^ According to the committee, this is due to a number of developments of which the introduction of prenatal screening in 2006 is the most important one. By means of this screening, foetuses with severe malformations are detected early in pregnancy and often not brought to term. The protocol may simply not be used anymore. Third, the discussion of euthanasia of severely disabled newborns is not new in the Netherlands and did not arise with the Groningen protocol. Indeed, it predated the legislation. Therefore, the legislation cannot be said to have caused any slide down a slope. Finally, it must be remembered that, contrary to the approach advocated by the Panel, the Netherlands has traditionally relied upon two bases for justifying its permissive regime for assisted suicide and euthanasia – autonomy and beneficence. It is beneficence that is used as the foundation for the Groningen Protocol. As this is not a basis relied upon by the Panel, it could not be used to justify any move to non-voluntary euthanasia in the regime proposed by the Panel.

In sum, there is no evidence from the Netherlands supporting the concern that society's vulnerable would be at increased risk of abuse if a more permissive regime were implemented in Canada.

## 5. CONCLUSIONS

In this chapter of the Report, the Panel has sought to describe the law in a number of jurisdictions where the approach to assisted dying (in one form or another) is somewhat permissive. It is evident from this survey that the societies which have acted on this issue are by no means homogeneous, yet they appear to share similar approaches to assisted dying. Whether or not it is voluntary euthanasia or assisted suicide that is involved (or indeed both), each jurisdiction (arguably with the exception of Montana, where the legal *status quo* came about in a very different way, and Switzerland where the provisions are primarily based only on intent) has premised permissibility (or lower likelihood of prosecution) on a number of conditions:

1. the request must be voluntary;
2. the request must be repeated (i.e. consistent over time);
3. the request must be certified by a physician
4. the request must be wholly or at least in part carried out by a physician;
5. the person making the request must be legally competent.

Different, although similar, provisions exist regarding the reporting of assisted deaths and safeguards are built in to try to ensure that the above requirements are verified.

The Panel notes that a policy of permitting at least some assisted dying often leads to ongoing discussions about reasonable limits on its practice. Assistance in dying is not only sought in cases of severe refractory physical suffering; requests may also partly or even predominantly originate from psychosocial or existential problems and in a number of cases the exact rationale for the request may not be identifiable. Legalization of assisted dying therefore inevitably involves further discussion of the boundaries of what is permissible. Should it, for example, be allowed for types of suffering that do not originate from physical diseases per se, such as being tired of life at a very old age? Furthermore, it can be questioned whether lawful assistance in dying should be the exclusive domain of physicians, especially when existential suffering is the problem at hand. When society asks physicians to evaluate existential judgements that they are not trained to evaluate, the result is not only an extraordinary emotional burden for physicians, but also arguably a threat to the credibility of the moral and legal frameworks that govern physician-assisted dying.

Despite the fears of opponents, it is also clear that the much-feared slippery slope has not emerged following decriminalization, at least not in those jurisdictions for which evidence is available. Nor is there evidence to support the claim that permitting doctors to participate in bringing about the death of a patient has harmed the doctor/patient relationship. What has emerged is evidence that the law is capable of managing the decriminalization of assisted dying and that state policies on this issue can reassure citizens of their safety and well-being.

# Sub article: CHAPTER FIVE: PROPOSALS FOR REFORM

**Published:** 2011-11

## 1. INTRODUCTION

The previous chapters have set the stage for this final chapter. There is a need and there are persuasive ethical arguments for reforms across the spectrum of end-of-life law and policy discussed to this point in this Report. As with the overview of the current legal status, the discussion of the proposed reforms will be organized in terms of the following categories: withholding and withdrawal of potentially life-sustaining treatment; potentially life-shortening symptom relief; terminal sedation; assisted suicide; and voluntary euthanasia. The Panel also offers recommendations with respect to palliative care as this is a critical component in any system designed to provide end-of-life care.

## 2. WITHHOLDING AND WITHDRAWAL OF POTENTIALLY LIFE-SUSTAINING TREATMENT

### a. Valid Refusals by Competent Adults (or Legally Authorized Substitute Decision-makers)

As noted in Chapter Two, there is some confusion in the minds of healthcare providers and members of the public as to the legal rights and responsibilities that arise in the case of the withholding and withdrawal of potentially life-sustaining treatment. This confusion may result in some legally valid refusals of treatment not being respected and some scarce medical resources being wasted on unwanted care. In order to avoid these undesirable consequences and as there is no controversy over what the law should be in this area (nor any reason for controversy from an ethical perspective), the Panel recommends that:

- The federal government should revise the *Criminal Code* to make it clear that the withholding and withdrawal of potentially life-sustaining treatment for which there has been a legally valid refusal does not constitute criminal negligence and will not attract criminal liability. This could be achieved, for example, by the federal government: adding the following to s.215 of the *Criminal Code*‘215(4)(e) Necessaries of life do not include medical treatment for which consent has been withheld or rescinded according to the relevant provincial or territorial law’; and adding the following to s.217 of the *Criminal Code*‘except where consent for the act has been withdrawn after the undertaking was made.’
- Unless or until the *Criminal Code* is revised as described above, prosecution guidelines should be drafted to make it clear that the withholding or withdrawal of potential life-sustaining treatment consistent with the law as described in Chapter Two, will not be subject to criminal prosecution.
- Healthcare professional educational institutions and regulators should ensure that their trainees and members understand their legal obligation to respect refusals of potentially life-sustaining treatment so that unwanted health services are not provided (i.e. legally valid refusals of treatment are not respected) out of misplaced fear of liability.
- The federal government in collaboration with provincial/territorial governments should educate the public regarding the legal status of the withholding and withdrawal of potentially life-sustaining treatment so that they can better advocate for themselves and their loved ones.

### b. Mature Minors

The issue of the possible limits on respect for the decisions of mature minors outlined in Chapter Two plays out across all of health care (for all treatment decisions including, for example, contraception, abortion, drug and alcohol addiction treatment, and mental health). A position on the issue can therefore only be adopted following a thorough review of the implications across the spectrum of potential applications. Since such a review would fall outside the time limits, mandate, and expertise of the Panel, the Panel determined that it would be inappropriate for it to make specific recommendations with respect to how the controversy identified in Chapter Two should be resolved. Rather, the Panel notes the importance of clarity with respect to the mature minor rule for end-of-life decision-making and recommends that this issue be taken up by provincial/territorial Departments of Health and Community Services (or equivalent governmental departments) as it can best be dealt with in a proactive way, through their consent and child protection legislation and through the relevant departments clarifying the consent law for mature minors in their jurisdiction (in whatever direction is deemed appropriate following a public consultation and legal and ethical analysis). The law on mature minors, as drafted in the various jurisdictions, should then apply to all aspects of end-of-life decision-making.

### c. Unilateral Withholding and Withdrawal

As noted in Chapter Two, there is a great deal of confusion and controversy over what the law should be with respect to unilateral withholding and withdrawal of potentially life-sustaining treatment. The current approach of leaving the resolution of the confusion and controversy to the courts does not serve anyone well. Family members of dying patients and healthcare providers find themselves locked in conflict; one side often feeling that they are fighting for their loved one's wishes and interests and the other side often feeling that they are fighting for the patient's interests and their own professional integrity. Litigation often seems the only route for conflict resolution and yet it is deeply corrosive of important relationships and distracts the participants from spending time with and caring for the patient. Furthermore, given the physical condition of most patients involved in such cases and given the time required for a case to work its way through the court system (especially for a matter of unsettled law), the results of litigation are often deeply unsatisfying for all involved.

As with the issue of mature minors, however, the issue of unilateral withholding and withdrawal plays out across many areas of health care and is not restricted to potentially life-sustaining treatment. There are many kinds of treatment that a patient or her substitute decision-maker might seek that a healthcare provider might wish, or feel compelled, to deny (because he believes that there are insufficient healthcare resources to meet the demand, it is not in the patient's best interests, or it does not meet the standard of care for medical practice).

A position on the unilateral issue can therefore only be adopted following a thorough review of the implications across the spectrum of potential applications. Again, since such a review would fall outside the time limits, mandate, and expertise of the Panel, the Panel determined that it would be inappropriate for it to make specific recommendations with respect to how the controversy identified in Chapter Two should be resolved. However, the Panel concluded that action is necessary to prevent a continuation of the cycle of damaging and inconclusive litigation.

In order to eliminate confusion and reduce the conflict and controversy surrounding unilateral withholding and withdrawal of potentially life-sustaining treatment, the Panel recommends the following:

1. Provincial/territorial governments should ensure that their consent legislation and healthcare professional regulators should ensure that their policies make it clear when, if ever, healthcare professionals have the legal authority to unilaterally withhold or withdraw potentially life-sustaining treatment.^333^
2. Health care professional educational institutions and regulators should ensure that their trainees and members understand their legal obligations with respect to unilateral withholding or withdrawal of potentially life-sustaining treatment.
3. The provincial/territorial governments should educate the public regarding the legal status of unilateral withholding and withdrawal of potentially life-sustaining treatment so that they can better advocate for themselves and their loved ones and better communicate with health care providers.

## 3. ADVANCE DIRECTIVES

The main problems with respect to advance directives and end-of-life care have less to do with the content of the law than with its implementation. As noted in Chapter Two, very few Canadians have completed advance directives and very little advance care planning is done. There is clear evidence of communication failures between patients, family members and healthcare providers. End-of-life issues remain topics avoided in routine clinical care despite an abundance of literature indicating that it is a topic many patients and families want to discuss. This is a matter of concern partly because it reflects a failure to engage in autonomy-enhancing care but also because it may result in some individuals receiving care that they do not want and some scarce medical resources being wasted on unwanted care. The Panel therefore recommends the following:

1. More research should be funded and conducted into how best to facilitate the completion of valid and useful advance directives and to engage in advance care planning.
2. Better education of healthcare providers and the public should be provided. If the public understands how to complete advance directives and the benefits of doing so, the completion rates and the validity and utility of the completed advance directives may increase with corresponding benefits for both the individuals and the health care system. If health care providers develop knowledge and skills with respect to communicating about end-of-life care and advance care planning, individuals' wishes may be more accessible to guide care.
3. More resources should be directed to encouraging and facilitating discussions of advance directives and advance care planning. For example, such discussions could be billable to provincial health care plans and individuals particularly skilled in such conversations should be available to patients in healthcare institutions.
4. More effective administrative mechanisms should be developed to ensure that the results of discussions of advance directives and advance care planning are made evident in a variety of contexts of care. For example, it should be (but often is not) possible for an advance directive and advance care plan to follow an individual seamlessly from an acute care setting to a long-term care facility and back again.

## 4. PALLIATIVE CARE

As described in Chapter One, the evidence is clear that most Canadians still die in institutions and also that the majority of Canadians lack access to quality end-of-life care. Many remediable gaps in care exist and much can be done to improve this situation. Indeed, a comprehensive set of recommendations with regard to the provision of palliative care services was recently articulated in a report authored by Senator Sharon Carstairs. The Panel found these recommendations to be consistent with the findings of its review of the literature on end-of-life care in Canada, as presented in Chapter Two, as well as the philosophical foundations for reform presented in Chapter Three. The Panel therefore endorses the recommendations contained within *Raising the Bar: A Roadmap for the Future of Palliative Care in Canada*.^334^ To these recommendations, the Panel adds the following:

1. Governments, health care institutions, and health care providers should work together to ensure that resources that could be better used for wanted palliative care are not diverted to unwanted acute care.
2. Palliative care specialists should continue to expand their scope beyond cancer and specialists in areas other than cancer care should continue to expand their understanding and use of palliative care.

Although the Panel strongly endorses all efforts to improve the quality of and access to palliative care in Canada it is of the opinion that this does not constitute a reason for not taking a more permissive approach to assisted suicide and euthanasia. There are two reasons for this. Data from the Netherlands, Belgium, and Oregon show that the need for assisted death is only partly dependent on the quality and availability of palliative care. Patients ask for assisted suicide or euthanasia not only because they are in pain but also because they evaluate their own situation as undignified.^335^ Patients ask for assisted suicide for reasons relating to autonomy and their own perceived quality of life.^336^ Palliative care would be able to respond to such concerns in some but certainly not in all cases. And even if it is the case that better palliative care would take away some of the need for assisted dying, it does not follow that a society should wait to have a policy on assisted dying until palliative care is optimized. It would constitute a reason for working on these improvements, but not a valid reason for telling people who are suffering right here and now to wait until these improvements have been realized.^337^

## 5. POTENTIALLY LIFE-SHORTENING SYMPTOM RELIEF

Given the uncertainty that exists about the legal status of potentially life-shortening symptom relief, the Panel is concerned that some patients are not receiving adequate symptom relief due to a misguided fear of liability. The Panel therefore recommends:

1. Healthcare providers, regulators, and prosecutors should collaborate on the development of guidelines with respect to what they consider to constitute the *Criminal Code* standards of ‘reasonable knowledge, skill and care’ and ‘wanton or reckless disregard’ in the context of the provision of potentially life-shortening symptom relief (each would promulgate their own – i.e. clinical practice guidelines for physicians and nurses and prosecutorial charging guidelines for prosecutors, but they would each benefit from collaboration with the other in the development phase).
2. Healthcare providers, institutions, and regulators and prosecution services should collaborate on the development and delivery of programs to educate the public and health care providers, regarding the fact that healthcare providers must provide symptom relief that accords with the guidelines and that they are protected from liability if they do.

## 6. TERMINAL SEDATION

In order to ensure that patients receive needed and wanted terminal sedation and that patients who seek terminal sedation in the absence of a need for it to relieve physical suffering receive all of the protections afforded in cases of voluntary euthanasia under the new proposed regime (see below), the Panel recommends the following:

- The federal government should revise the *Criminal Code* to make it clear that terminal sedation in circumstances where it is not required to alleviate physical suffering should be considered euthanasia and be subject to the same procedural conditions and requirements as other forms of euthanasia (see below).
- Healthcare providers, regulators, and prosecutors should collaborate on the development of guidelines with respect to what they consider to constitute the *Criminal Code* standards of ‘reasonable knowledge, skill and care’ and ‘wanton or reckless disregard’ in the context of the provision of terminal sedation (each would promulgate their own clinical practice guidelines for physicians and nurses and prosecutorial charging guidelines for prosecutors, but they would each benefit from collaboration with the other).
- The federal government in collaboration with the provincial/territorial governments should develop and deliver programs to educate the public and healthcare providers, regarding the fact that healthcare providers must provide terminal sedation that accords with the guidelines and that they are protected from liability if they do.

## 7. ASSISTED SUICIDE AND VOLUNTARY EUTHANASIA

For the reasons articulated in Chapter Three, the Panel was persuaded that the law in Canada as described in Chapter Two should be changed to allow some form of assisted suicide and voluntary euthanasia. Putting the philosophical analysis together with the lessons learned from Chapter Four's review of the paths taken in other jurisdictions that have moved to more permissive regimes, the Panel considered the options for the design of a permissive regime and suggests the following legal mechanisms for achieving the reform and the core elements of the proposed reform. The Panel begins by discussing the different options in rank order of preference (in other words, the Panel recommends reform of the *Criminal Code* and, only if that is not taken up, development of prosecutorial charging guidelines and, only if that is not taken up, development of diversion programs).

### a. Legal Mechanisms

#### i. Criminal Code Reform

The *Criminal Code* falls within the jurisdiction of the federal government and so only the Parliament of Canada can proactively reform the *Criminal Code*.^338^ To date, all bills aimed at decriminalizing assisted suicide and/or voluntary euthanasia have been private members' bills.^339^ None have been passed by the House. However, with a bill drawn up to avoid the weaknesses of the previous attempts in this area^340^ and if the public presses Parliament for change, legislative reform might be possible.

The Panel concludes that revision of the *Criminal Code* is the best available mechanism for legal reform for a number of reasons. First, as it flows from the federal Parliament, it provides for the greatest consistency of approach across all of the provinces and territories. There is merit in treating an issue of such extraordinary social significance grounded in constitutional values as consistently across the country as possible. Second, again, as it flows from the federal Parliament, it allows for the creation of a national oversight body that could ensure an accurate and comprehensive picture of what is happening in the area of assisted suicide and voluntary euthanasia across the country. This would be efficient, protective, and reassuring as well as in line with best international practice. Third, it avoids leaving the burden of changing the law on the backs of individuals who wish to access assisted suicide or voluntary euthanasia. Without leadership from Parliament, it is left to individuals to challenge the constitutionality of the law through the emotionally, physically, and financially draining court system. Finally, it provides the greatest level of certainty to Canadians with respect to their rights and responsibilities. The other options also recommended by the Panel (see below) have their strengths, but they involve a measure of discretion that leaves the consequences of a particular act of assisted suicide or voluntary euthanasia less certain. This means that some cases of what the Panel would deem morally acceptable voluntary euthanasia or assisted suicide might not happen for fear of liability or, if they do happen, it may be under the unsettling shadow of that fear.

Therefore, for these and for the reasons articulated in Chapter Three, the Panel recommends that the prohibitions on assisted suicide and voluntary euthanasia in the *Criminal Code* be modified such that, in carefully circumscribed and monitored circumstances, they are legally permissible (see a later section of this chapter for an explication of the proposed circumscription and monitoring).

#### ii. Prosecutorial Charging Guidelines

A second-best path to reform lies in the revision of prosecution services policy manuals. Guidelines for the exercise of prosecutorial discretion could spell out the circumstances in which cases of assisted suicide or voluntary euthanasia will not result in charges of assisted suicide or murder being laid.

The Crown, through prosecutors, has discretion with respect to the pursuit and withdrawal of charges under the *Criminal Code*.^341^ This is known as ‘the prerogative of prosecutorial discretion’ or simply ‘prosecutorial discretion.’ Assistance, guidance, and sometimes direction on the exercise of prosecutorial discretion is provided to individual prosecutors through guidelines found in Crown Policy Manuals under the authority of the federal or provincial Attorney General.^342^ Generally, charges should only be laid where there is a substantial (or what could be alternately worded as, for example, a realistic or reasonable) likelihood of conviction and where prosecution is in the public interest.

The exercise of prosecutorial discretion can be manifest through charging with a lesser offence (for example, manslaughter instead of murder) or withdrawing the charges. This has obvious significant implications as, for example, murder carries with it a mandatory minimum life sentence while manslaughter has no mandatory minimum sentence. The exercise of prosecutorial discretion can mean the difference between no criminal record, a conviction but no jail time, or a conviction and mandatory life sentence in jail.

It is within the mandate of provincial/territorial Attorneys General and prosecution services to establish policies and processes that, while not decriminalizing assisted suicide and voluntary euthanasia (only the federal government can do that), shape how the *Criminal Code* provisions are administered within their jurisdiction. The Panel recommends that, unless or until the *Criminal Code* is reformed as recommended above, those with authority over prosecutorial policies in all provinces and territories introduce such policies to provide guidance with respect to the exercise of prosecutorial discretion and to make clear the circumstances within which a prosecution for assisted suicide or voluntary euthanasia would not be in order (these circumstances are spelled out in a later section of this chapter).^343^

#### iii. Diversion Programs

Where programs have been established by the province or territory, before or even during a prosecution, prosecutors have the discretion to divert a case away from the traditional court system to alternative pathways (e.g. community service, restitution, reconciliation, education).^344^ This discretionary authority has been shaped by various statutes, regulations, and policies resulting in a variety of diversion programs.

Judges also have significant discretion with respect to diverting individuals charged under the *Criminal Code* at the sentencing stage of the process. While the independence of the bench is very strong, it is limited insofar as judges are bound by the rule of law (e.g. mandatory minimum sentences, *Criminal Code* definitions of the elements of a criminal offence). Judicial discretion can be exercised in relation to determinations of how the law applies to the specific facts of a case, the interpretation of laws, and the authority to seek input and recommendations from third parties and to issue all different manner of sentences which accord with the sentencing principles in the *Criminal Code* (e.g. prison terms, community service, and unconditional discharge). As an alternative to the traditional adversarial trial process, a judge can suggest that a defendant take part in an alternative process and then use information from that process to inform the sentencing decision (e.g. an unconditional or conditional discharge instead of jail time).

The Panel was struck by the idea of introducing a restorative justice option^345^ as a diversion program available to judges and prosecutors in cases of assisted suicide and voluntary euthanasia. As restorative justice will be an unfamiliar concept and restorative justice programs an unfamiliar phenomenon to most readers, a brief description is in order here, particularly in relating it to assisted suicide and voluntary euthanasia.^346^

Restorative justice processes are fundamentally oriented to recognizing, understanding, characterizing, and responding to harms. Restorative justice focuses on harms not only to individuals but also to the relationships between and among the parties involved and, even more broadly, to social values (as these are, at least in part, what bind us together). It also focuses on harms caused not only by individual conduct but also by institutional practices and social structures.

Restorative justice processes involve a variety of individuals in their attempts to understand and respond to harms. In contrast to the traditional criminal justice processes, restorative processes provide a forum in which all of those with a stake in the outcome of the situation (narrowly and broadly understood) can come together to discuss the issues and consider appropriate responses. Such processes provide a public space in which to reflect upon all of the issues and interests raised by the cases. This makes them particularly adept at determining the public interest. By bringing into the process those more broadly engaged and affected by a situation, they facilitate the recognition of the community and public stake in the issues and their resolution and the significance of public participation and accountability in and through these processes. From this, such processes are able to affirm, challenge and even begin to reform public values and policies.

When they come together, the stakeholders explore the individual and social impact on the relationships between themselves and others. They also work to hold individuals and institutions accountable for the harm they caused and to facilitate planning amongst the parties aimed at addressing the resulting and related harm and creating the conditions to prevent future harms. Restorative processes are not only attentive to the facts of what happened, but are committed to exploring their contexts and causes in order to consider a future focused response. They can consider and respond to the full range of legal, social and ethical issues raised and the roles and responsibilities of everyone involved.

As a result of their broader orientation and engagements, restorative justice processes are able to arrive at a richer understanding of what led to the harms (individual conduct, institutional practices, and a wide variety of background conditions), the interests and values that are at stake, and a broader range of responses that are seen as needed and appropriate. Despite a common misconception to the contrary, restorative justice processes do more than simply ‘restore’ the pre-harm status quo.

Restorative justice processes thus understood seem particularly well-suited for service as diversion programs for cases involving assisted suicide or voluntary euthanasia (assuming that the public policy decision, that this Panel has rejected, is made to continue to treat assisted suicide and voluntary euthanasia as crimes). There is good evidence that these processes work particularly well for conduct characterized as serious crimes and in cases involving relational harms.^347^

Relational harms are very much at issue in cases of assisted suicide and voluntary euthanasia not only at the micro level in terms of the relationships between particular parties (healthcare professional, patient, family members, etc.), but also at a macro level in terms of the potential harm to broader social values (respect for autonomy, life, etc.).

Bringing together a variety of stakeholders could be particularly fruitful in the context of assisted suicide and voluntary euthanasia cases. For example, those charged under the *Criminal Code* could be brought together with others directly affected by their actions (other family members, for example) together with health professionals, ethicists, justice officials, community members, etc. In this space, unlike in the criminal justice system, the parties would not be restricted in their focus to only the facts of the case and the acts of the particular offender charged.

In the case of assisted suicide and voluntary euthanasia, it can be particularly useful to be able to take into account avoidable institutional, systemic, or societal factors that may have played into or structured the decision to seek assisted suicide or voluntary euthanasia (e.g. where a lack of resources with respect to adequate pain management contributed to the decision; in such a case the decision might be understandable and justifiable but nevertheless warrant attention and reflection from the system actors to determine whether changes need to or ought to be made in order to provide different options about which autonomous decisions can be made). Restorative processes are able to lift up and respond to such background contexts and causes.

In light of this understanding of restorative justice processes, the Panel recommends that, unless or until the *Criminal Code* is reformed or prosecutorial charging guidelines are implemented as recommended above, provinces and territories consider implementing a restorative justice process for assisted suicide and voluntary euthanasia cases. Such processes can be turned to by prosecutors in the exercise of their discretion over which charges to pursue, if any, and judges, in the exercise of their discretion in respect to sentencing in cases of assisted death. With respect to judicial discretion, for all but voluntary euthanasia charged as murder, participation in a restorative justice program could lead to a full range of sentences (including discharge). For voluntary euthanasia charged as murder, participation could affect how high above the mandatory minimum sentence the judge would go as well as the length of parole decisions (again above the mandatory minima). The Panel notes that Nova Scotia is particularly well-situated to pilot such a process as it has a uniquely well-developed restorative justice program for youth, which is currently, on a pilot basis, being expanded to include some adults and so has the expertise and infrastructure necessary for such an initiative.

### b. Core Elements

Once the legal mechanism(s) for achieving law reform have been settled upon, designers of any permissive regime must take and defend positions on a set of core elements. The following represents the Panel's considered positions on the core elements of a permissive regime. They flow from the ethical analysis set out in Chapter Three. Where the reasons for the position taken may not be immediately obvious from the content of Chapter Three, the Panel here provides additional commentary.

#### i. Assisted Suicide and/or Voluntary Euthanasia

Both assisted suicide and voluntary euthanasia must be available. The Panel found no morally significant difference between these two activities and, furthermore, found that the arguments canvassed in Chapter Three and the evidence presented in Chapters Two and Four undercut prohibiting either and support permitting both.

#### ii. Features of the Person

The person making the request for assisted suicide or euthanasia must be competent or, while competent, must have expressed the wish for voluntary euthanasia through a valid advance directive. Great care must be taken to ensure that, at the time of the decision, the person is able to understand and appreciate the nature and consequences of the decision. However, this level of care is not unique to assisted suicide and euthanasia. Many healthcare decisions (e.g. risky surgery and cessation of treatment) bring with them the possibility or even certainty of death and many require the ability to understand more complex information than is required to decide whether to commit suicide. For the same reason, there is also no justification for requiring unique skills in competency assessment of the health care providers in the context of assisted suicide or euthanasia. Of course, as with any assessment of competence for the purposes of health care decision-making, if an individual physician is uncertain about the competence of the person making a request, she must take all necessary steps to resolve this uncertainty (e.g. consulting with a colleague with greater experience or expertise).

Any age restrictions for access to assisted suicide or voluntary euthanasia should flow from the mature minor law in the particular jurisdiction.

#### iii. Features of the Decision

The decision must be voluntary and informed. Again, great care must be taken to ensure that these conditions are met.

#### iv. Features of the Person's Condition

The Panel recommends against using ‘terminal illness’ as a prerequisite for requesting assistance. The term is too vague and would leave the statute or policy open to a *Charter* challenge. There is no precise science to providing a prognosis of a terminal illness in terms of a specific length of time. Healthcare providers cannot be accurate enough, and if the statute or policy does not include a time restriction then the condition ‘terminal illness’ becomes too broad. For example, a person with Guillain-Barré syndrome will die from her disease, but lives in the average three years after diagnosis. Further, if the term ‘terminal illness’ is made a necessary condition in the statute, then it would be under-inclusive; there are many individuals whose lives, to them, are no longer worth living, who have not been diagnosed with a terminal illness. They may be suffering greatly and permanently, but are not imminently dying. There is no principled basis for excluding them from assisted suicide or voluntary euthanasia.

#### v. Features of the Request for Assistance

Written or otherwise recorded requests are preferable (as they can be more reliable as evidence that a request was made and what the request was for), but verbal requests are sufficient, if properly documented.

The time required to elapse between the initial request and the granting of the assistance will depend on the time required to ensure that the person's request is voluntary and informed and that the individual is competent (or was competent at the time of making an advance directive). Depending on the circumstances, this may take a short or quite a long time. Once all of the other conditions have been met, there must be a short (for example, twenty-four hours) pause before the assistance is provided to allow confidence that all of the conditions and procedural requirements have been met. Beyond that, the Panel does not recommend any delay requirements.

#### vi. Features of the Provider

The Panel recommends that healthcare professionals be permitted to provide assistance with suicide or voluntary euthanasia. For the reasons expressed in Chapter Three, the Panel concludes that health care professionals are not obligated to provide assistance. However, should they decide not to provide assistance they are obligated to pass the person requesting assistance on to a professional who will provide such assistance. An open question is whether only health care professionals should be permitted to provide assistance. These two issues are interrelated and dependent upon the mechanisms available to protect the patients while minimizing the limits on the autonomy of healthcare professionals and maximizing respect for the autonomy of those seeking assistance. The availability of various potential mechanisms and the balancing that must follow rests upon empirical evidence that was not available to the Panel. The Panel recommends that permission to provide assistance be granted only to those: who have the knowledge and skills necessary to ensure that the conditions for access laid out earlier in this section (e.g. competence, voluntariness, conveyance of information) have been met; and with whom the oversight system can meaningfully function. Furthermore, the more restrictive the list of those who are permitted to provide assistance, the less that group should be permitted to refuse to provide assistance.

#### vii. Oversight and Control

As was shown in Chapter 4, different approaches are taken with respect to oversight in different jurisdictions. There is variability in the legal position of these oversight entities, their constitution, authority, and objectives. The Dutch, Belgian and Luxemburg systems provide for a case-by-case evaluation both to keep an inventory and also to judge whether the requirements established by law were met in individual cases. The Oregon and Washington State bodies do not judge individual cases but rather are tasked with collecting information and reporting at an aggregate level. Interestingly, none of the existing permissive regimes use prospective rather than retrospective oversight.

The Panel recommends that a national oversight commission be established to monitor and report annually and publicly on assisted suicide and voluntary euthanasia in Canada.^348^ The Panel sees two roles for this oversight body. The first role is maintenance of public trust in the system. The second is the prevention of mistaken or intentional violations of the new law. The means of realizing the first objective is the collection of data and the reporting of the data in aggregated form. The means of realizing the second is expert assessment of specific cases with appropriate follow-up, which could be engagement with specific individual providers or more general education programs for health care providers or the general public. Obviously, the coroners, police and prosecution services will continue to have the authority and responsibility to investigate and prosecute potential violations of the law.

The Panel recommends that requirements for assessments, declarations of request, statements of reasons for requests, and document filing be set out in statute and be designed to minimize the intrusion on the person seeking assistance, but also be sufficient to make possible effective oversight so as to ensure that only requests for assistance that fit within the autonomy-based analysis are respected and the public trust is maintained.

## Footnotes

333 For example, provincial consent legislation could include the following statement and definitions (a modified version of the provisions in the Ontario *Health Care Consent Act, 1996* S.O. 1996, c.2, Sch.A., ss.2 and 10): “A health practitioner who proposes a treatment for a person shall not proceed with the treatment, and shall take reasonable steps to ensure that it is not administered, unless, (a) he or she is of the opinion that the person is capable with respect to the treatment, and the person has given consent; or (b) he or she is of the opinion that the person is incapable with respect to the treatment, and the person's substitute decision-maker has given consent on the person's behalf in accordance with this Act. “Treatment” means anything that is done for a therapeutic, preventive, palliative, diagnostic, cosmetic, or other health-related purpose. “Treatment plan” means a plan that: (a) is developed by one or more health practitioners; (b) deals with one or more of the health problems that a person has and may, in addition, deal with one or more of the health problems that the person is likely to have in the future given the person's current health condition; and (c) provides for the administration to the person of various treatments or courses of treatment and may, in addition, provide for the withholding or withdrawal of treatment in light of the person's current health condition. A sample policy statement for a health care regulator is The Manitoba College of Physicians and Surgeons Position Statement on Withholding and Withdrawal of Life-Sustaining Treatment. An alternate policy statement for a health care regulator is found in DownieJMcEwenKThe Manitoba College of Physicians and Surgeons Position Statement on Withholding and Withdrawal of Life-Sustaining Treatment (2008): Three Problems and a SolutionHealth Law Journal20101711513820608317.

334 Available at <http://sen.parl.gc.ca/scarstairs/PalliativeCare/PalliativeCare_e.asp>. [Accessed 26 July 2011].

335 Onwuteaka-Philipsen BD et al. *Evaluatie wet toetsing levensbeëindiging op verzoek en hulp bij zelfdoding*. Den Haag: ZONMW; 2007.

336 GanziniLGoyERDobschaSKOregonians’ reasons for requesting physician aid in dyingArchives of Internal Medicine2009169548992Mar 91927377910.1001/archinternmed.2008.579. Erratum in: Arch Intern Med. 2009 March 23;169(6):571.

337 See also Delden JJM van, Battin MP. Euthanasia: not just for rich countries. In: Green R, Donovan A (eds). Global bioethics. Oxford: Oxford University Press: 243–261.

338 The Supreme Court of Canada can, in effect, change the *Criminal Code* as it can strike down or read in content to ensure that the *Criminal Code* is consistent with the *Charter*. This is, however, reactive as a case must come before the Court before it can take such steps.

339 The legislative history is reviewed in Marlisa Tiedemann and Dominique Valiquet in a Library of Parliament Report “Euthanasia and Assisted Suicide in Canada” 91–9E (revised 17 July 2008). Available online at http://www2.parl.gc.ca/content/lop/researchpublications/919-e.htm#parliamentary. [Accessed 26 July 2011].

340 An example of such a bill can be found in DownieJBernS“*Rodriguez Redux*”Health Law Journal200816275419536977.

341 Prosecutorial discretion is discussed by the Supreme Court of Canada in, for example, *R. v Lyons. Supreme Court Reports*. 1987;2:309. *R. v Beare*. *Supreme Court Reports.* 1988;2:387. *R. v Power*. *Supreme Court Reports*. 1994;1:601. and *Fraser v Public Service Staff Relations Board*. *Supreme Court of Canada*. 1985;2:455.

342 While some jurisdictions have created independent public prosecution services (e.g., Nova Scotia under the *Public Prosecutions Act*, S.N. 1990, c.21, all have retained a policy power for the Attorney General.

343 Examples of guidelines for the exercise of prosecutorial discretion in relation to some aspects of end of life law, policy, and practice include: Criminal Justice Branch, Ministry of Attorney General. Crown Counsel Policy Manual – Euthanasia and Assisted Suicide. March 15, 2004. Available at http://www.ag.gov.bc.ca/prosecution-service/policy-man/pdf/EUT1-EuthanasiaAndAssistedSuicide-15Mar2004.pdf; [Accessed 26 July 2011]; The Crown Prosecution Service. Policy for Prosecutors in Respect of Cases of Encouraging or Assisting Suicide. February 2010. Available at http://www.cps.gov.uk/publications/prosecution/assisted_suicide_policy.html. [Accessed 26 July 2011].

344 For example, see “Alternative Measures – Adult Diversion”“approved by the Attorney General of Nova Scotia, pursuant to s.717 of the Criminal Code of Canada”. Available at http://www.gov.ns.ca/pps/publications/ca_manual/ProsecutionPolicies/Restorativejustice.pdf. [Accessed 26 July 2011].

345 For an example of a restorative justice program (for youth aged 12–17 charged with a particular set of criminal offences), see the description of the restorative justice program in Nova Scotia. Available at http://gov.ns.ca/just/rj. For more information and resources on restorative justice, see the Nova Scotia Restorative Justice Community University Research Alliance. Available at <http://www.nsrj-cura.ca> [Accessed 26 July 2011].

346 For a detailed discussion of the use of restorative justice in the context of assisted suicide and euthanasia, see Llewellyn J and Downie J. “Restorative Justice, Euthanasia, and Assisted Suicide A New Arena for Restorative Justice and A New Path for End of Life Law and Policy in Canada”*Alberta Law Review*, Special Issue on Restorative Justice, 2011: 48:4.

347 Sherman LW, and Strang H. 2007. *Restorative Justice: The Evidence*. London: Smith Institute.

348 An example of draft legislation to describe and establish these oversight functions and a national oversight commission is included in DownieJ.BernS“*Rodriguez Redux*”Health Law Journal200816275419536977.
